# Homogeneous Catalysis for Sustainable Energy: Hydrogen and Methanol Economies, Fuels from Biomass, and Related Topics

**DOI:** 10.1021/acs.chemrev.1c00412

**Published:** 2021-11-02

**Authors:** Amit Kumar, Prosenjit Daw, David Milstein

**Affiliations:** † School of Chemistry, University of St. Andrews, North Haugh, Fife, U.K., KY16 9ST; ‡ Department of Chemical Sciences, Indian Institute of Science Education and Research Berhampur, Govt. ITI (transit Campus), Berhampur 760010, India; § Department of Molecular Chemistry and Materials Science, Weizmann Institute of Science, Rehovot 76100, Israel

## Abstract

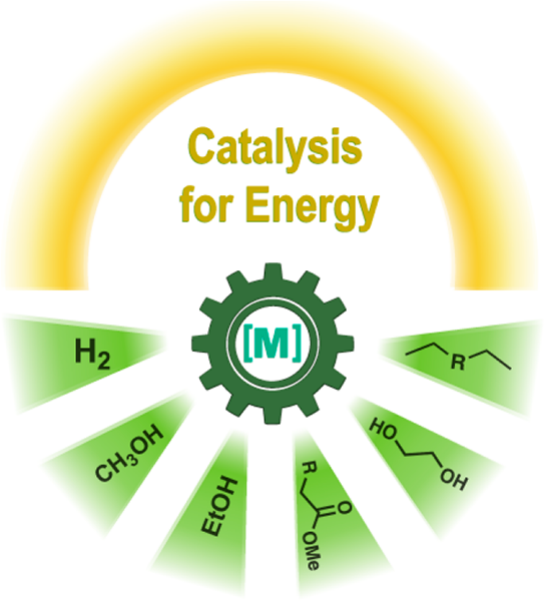

As the world pledges to significantly cut carbon emissions, the demand for sustainable and clean energy has now become more important than ever. This includes both production and storage of energy carriers, a majority of which involve catalytic reactions. This article reviews recent developments of homogeneous catalysts in emerging applications of sustainable energy. The most important focus has been on hydrogen storage as several efficient homogeneous catalysts have been reported recently for (de)hydrogenative transformations promising to the hydrogen economy. Another direction that has been extensively covered in this review is that of the methanol economy. Homogeneous catalysts investigated for the production of methanol from CO_2_, CO, and HCOOH have been discussed in detail. Moreover, catalytic processes for the production of conventional fuels (higher alkanes such as diesel, wax) from biomass or lower alkanes have also been discussed. A section has also been dedicated to the production of ethylene glycol from CO and H_2_ using homogeneous catalysts. Well-defined transition metal complexes, in particular, pincer complexes, have been discussed in more detail due to their high activity and well-studied mechanisms.

## 1 Introduction

Energy lies at the core of a nation’s economy, and for the past two centuries, the amount of the energy consumption of society has increased in lockstep with the amount of wealth created. Previous data indicate a correlation between energy consumption and gross domestic product (GDP) implicating increased demand for energy globally, especially for the developing economy.^1^ On the basis of the recent data, the world’s primary energy source constitutes petroleum (34%), coal (27%), and natural gas (24%), making 85% of the energy sources to be fossil fuels.^2^ Heavy consumption of fossil fuels is not sustainable as they take millions of years to form and the limited supply of fossil fuels is being depleted at a much faster rate than they are being produced. Additionally, the consumption of fossil fuels raises serious environmental and health concerns. For example, a vast majority of deaths due to air pollution are caused due to fossil fuel consumption and more than 3 million lives would be saved every year if cleaner energy were used instead.^3^ Unsurprisingly, a direct correlation between GDP and the amount of CO_2_ being produced also exists.^4,5^ Thus, there is an urgent need to develop sustainable and clean energy carriers.

Several alternate energy sources such as solar, wind, ocean (tidal, wave, thermal), biomass, nuclear, and geothermal have been well-studied in the past, where each one has its own limitations, and for a practical scenario in the near future, a combination of multiple renewable energy sources together with fossil fuels will be needed to keep the planet sustainable and green.^6−8^ Pursuit of a suitable carrier for the production, storage, and use of energy in a clean, economical, and sustainable way has created the need for the development of efficient catalysts to advance this goal. In general, catalytic technologies play crucial roles in the following sections of the energy sector: (a) energy production reactions, (b) safe and long-term energy storage and transportation, and (c) high efficiency of energy use. Multiple review articles have been reported recently on the application of heterogeneous catalysts,^9−13^ photocatalysts,^14,15^ and electrocatalysts^16−21^ for the production and storage of energy, and these subdisciplines of catalysis will not be discussed here.

Homogeneous catalysis allows the processes to occur under relatively mild conditions and at the same time advances our understanding of reaction mechanisms at the molecular level, thus providing remarkable opportunities to improve the catalytic processes.^22^ Here, we review reports on homogeneous catalysis based on transition metal complexes for their applications in the development of clean and sustainable energy carriers. Emphasis has been given to complexes based on pincer^23^ type ligands as they have led this area and let some remarkable discoveries happen. Pincer ligands are defined as chelating ligands that bind through three adjacent donor sites in a meridional geometry.^24^ The choice of topics as detailed below is based on energy production or storage systems in which homogeneous transition metal catalysis has played a prominent role.

H_2_, long considered a “fuel of the future”, in the past decade has climbed its way up to the stage where a hydrogen economy^25^ is looking promising in the foreseeable future. Several well-defined transition metal catalysts have been investigated in the past to impact the hydrogen economy, especially to discover new hydrogen storage systems. Section 2 of this review discusses various hydrogen storage systems and the development of metal complexes as catalysts for the charge and discharge of H_2_. Aqueous reforming of methanol (CH_3_OH + H_2_O = 3H_2_ + CO_2_) using both precious metal and earth abundant metal based catalysts have been discussed in detail because of the potential application of this reaction to impact both the hydrogen economy and the methanol economy (section 2.1).^26^ Recent developments in the direction of production of H_2_ from formaldehyde/paraformaldehyde have also been discussed (section 2.2). The reaction is catalyzed by ruthenium or iridium complexes and produces CO_2_ or carbonate salts as byproducts. Of many inorganic materials explored for the purpose of hydrogen storage, amine-boranes have been the most studied by the community of organometallic chemists. Catalytic dehydrogenation of amine-boranes has only been briefly discussed here as multiple review articles have been reported in the past few years on this topic (section 2.3).^27−36^ Despite significant developments in the dehydrogenation of various amine-boranes, a practical technology for the regeneration of “charged fuel” (amine-boranes) from “spent fuel” (e.g., borazine) is yet to be developed. A perspective on the regeneration of amine-borane “charged fuel” and developments in the area of hybrid cyclic amine-boranes has also been detailed (section 2.3.3). The quest of developing reversible hydrogen storage materials has led to the development of “liquid organic hydrogen carriers” (LOHCs). Various LOHCs have been developed in the past; some of them have also been commercialized.^37^ Recent advances in the development of various LOHCs using organometallic catalysts have been discussed in detail (section 2.4). A brief section on the production of H_2_ from biomass and water splitting has also been included here (section 2.5).

The use of renewable feedstock or biomass to produce valuable chemicals and energy carriers lies at the heart of the circular economy model.^38−40^ Fuels produced from biomass or “biofuels” can be categorized into three types. The first type, called the “first-generation biofuels”, mainly involving bioethanol and biodiesel, is produced from edible biomass such as sugars, grains, or seeds.^41^ Both bioethanol and biodiesels can be used to substitute petrol and diesel respectively, most commonly as blends with conventional fuels. Bioethanol is produced through the fermentation of sugar via enzymatic catalysis,^42,43^ whereas biodiesel is produced through the transesterification of vegetable oils or fats for which several types of catalysts, e.g., acid/base catalysts,^44^ enzymes,^45^ and heterogeneous catalysts,^46,47^ have been reported. Recent advances have demonstrated that ethanol can be converted to butanol and other higher alcohols which have multiple advantages (e.g., high energy density, noncorrosive nature, and immiscibility with water) over ethanol, making them closer to the conventional gasoline fuels. We have reviewed here recent progress made in the direction of transforming ethanol to butanol and higher alcohols using organometallic catalysts (section 3.1). Additionally, we have also discussed reports on the catalytic hydrogenation of the unsaturated C=C bonds in the fatty acid methyl esters to upgrade biodiesels by improving their multiple properties, e.g., stability, and lubricity (section 3.2). Technologies for the production of first-generation biofuels are well-established and are operating around the globe. However, it is important to note that the first-generation biofuels are produced from edible food crops that make their production process compete with the land and water used for food and it has been claimed that they have driven up the cost of food and animal feeds.^48,49^

The second type of biofuel, called “second-generation biofuels”, is sourced from nonedible biomass, e.g., lignocellulose (e.g., cereal straw, sugar cane bagasse, and organic waste). This avoids the complexity of the societal implication of using food to produce fuels. Technologies to transform lignocellulose to fuels, especially alternative jet fuels, are being evaluated.^50,51^ These processes involve catalytic pyrolysis or gasification and Fischer–Tropsch reactions followed by hydrotreatment.^52,53^ The main limitation of “second-generation biofuel” is the requirement of sophisticated processing and production equipment as the complex 3-D structure of lignocellulose makes it difficult to depolymerize. Several heterogeneous and acid/base catalysts have been used to depolymerize lignin under harsh conditions as reported in the past.^54−62^ Homogeneous transition metal catalysis has also been employed to break model compounds of lignin as discussed in this review (section 3.3).

The third type of biofuel, called “third-generation biofuels”, involves fuels derived from microorganisms, e.g., algae. Despite some important advantages such as high ignition points, and biodegradability, the high cost associated with the production of fuels from algae has limited its scope for commercial purposes.^63^

Another area that has attracted significant interest in the past decade is that of the methanol economy. Methanol is an important chemical feedstock for plastics, glue, paints, building materials, and solvents and is produced on a scale of more than 75 million tons annually worldwide. Its additional application as a fuel (or fuel additive) has sparked further interest in this area. A sustainable, cost-effective, and large-scale production of methanol from captured CO_2_ would be highly useful to enable a “circular economy”^38^ as also proposed by Olah and Prakash as the concept of “methanol economy”.^64^ Beyond the concept, this vision has been realized by an Iceland-based company called Carbon Recycling International (CRI), where the CO_2_ captured from industrial emissions is hydrogenated to methanol using renewable H_2_. The renewable H_2_ can come from the electrolysis of water using renewable electricity (making renewable e-methanol or Vulcanol) or from the byproduct or waste gas (making low carbon methanol). Since 2021, Vulcanol has been commercially sold in Europe and China, and CRI aims to produce up to 110 000 tons of recycled carbon methanol annually from 2021.^65^ Another company that makes renewable methanol on an industrial scale is the Canada-based company called Enerkem,^66^ where methanol is produced from municipal solid waste via thermochemical gasification of organic waste to produce syngas followed by the catalytic conversion of syngas to methanol. The Netherland-based company called BioMCN^67^ produces renewable methanol from biogas sourced from waste digestion plants. In 2017, BioMCN produced and sold around 60 000 tons of renewable methanol. It is clear that the production of renewable methanol is still much lower than its demand, which is partly due to the lack of an abundant supply of inexpensive renewable feedstocks or renewable energy in the local region. Considering that the production and release of CO_2_ in the atmosphere is inevitable, its capture and transformation to methanol in a cost-effective manner is perhaps the most sustainable approach to produce renewable methanol. This area has been extensively studied using organometallic catalysts where CO_2_ can be trapped using capturing agents such as alcohols, amines, silanes, and boranes followed by its hydrogenation or hydrolysis to produce methanol. We have reviewed here examples of the indirect transformation of CO_2_ to methanol using homogeneous catalysts (section 4.1). A brief discussion on the production of methanol from CO (section 4.2), HCOOH (section 4.3), and CH_4_ (section 4.4) catalyzed by transition metal complexes has also been reported.

The above-described topics investigate the pursuit of alternative energy sources such as H_2_, methanol, and biofuels. Another approach to manifest sustainable energy can be to develop methods to convert renewable feedstock to conventional fossil fuels. For example, lower alkanes, e.g., methane, can be produced from biomass or CO_2_. Thus, its transformation to produce higher hydrocarbons (e.g., C8–C19) can allow us to attain a sustainable energy-based economy without a need to change our infrastructure to accommodate a new energy source. This is particularly important for aviation fuels, where density requirements and stringent specifications dictate that jet fuel (C8–C16 hydrocarbons) will be the industry norm in the near future.^68^ The area of alkane upgradation has made slow progress in the past couple of decades due to the inertness and inactivity of alkane C–H bonds. Two approaches using homogeneous catalysts have been utilized for alkane upgradation and demonstrate promise in this direction. We have reviewed here these two approaches that are based on alkane metathesis (section 5.1) and alkane–alkene coupling (section 5.2). A brief section on the use of ethylene glycol as a fuel and its production using homogeneous catalysts has also been presented (section 6). Finally, a summary and perspective on the current challenges and prospects of the reviewed areas have been described (section 7).

### 1.1 General Mechanistic Consideration

A drive to study homogeneous catalysis is the opportunity to understand the mechanisms of catalytic processes which eventually allows us to develop new and more efficient catalysts. As we review a plethora of homogeneous catalysts for the production and storage of energy carriers, in this section, we present a general overview of their mechanism of operation. Many such catalysts operate via redox innocence metal–ligand cooperation (MLC) featuring a basic or nucleophilic site on the ligand, and a coordinatively unsaturated metal center that can act as an electrophile. This allows heterolytic bond activation of polar (e.g., O–H, N–H) and nonpolar (e.g., H_2_) molecules across the electrophilic metal center and the nucleophilic ligand site. Overall, the oxidation state of the metal remains the same, unlike the classical mode of bond activation by oxidative addition and reductive elimination, where the oxidation state of the metal changes by two units. This has allowed the utilization of this concept in bond activation and catalysis to various elements (including main-group elements such as boron^69^ and zinc^70^) across the periodic table. Overall, most of the catalysts discussed in this article can be broadly categorized into the following types depending on their mode of operation.

**Scheme 1:**
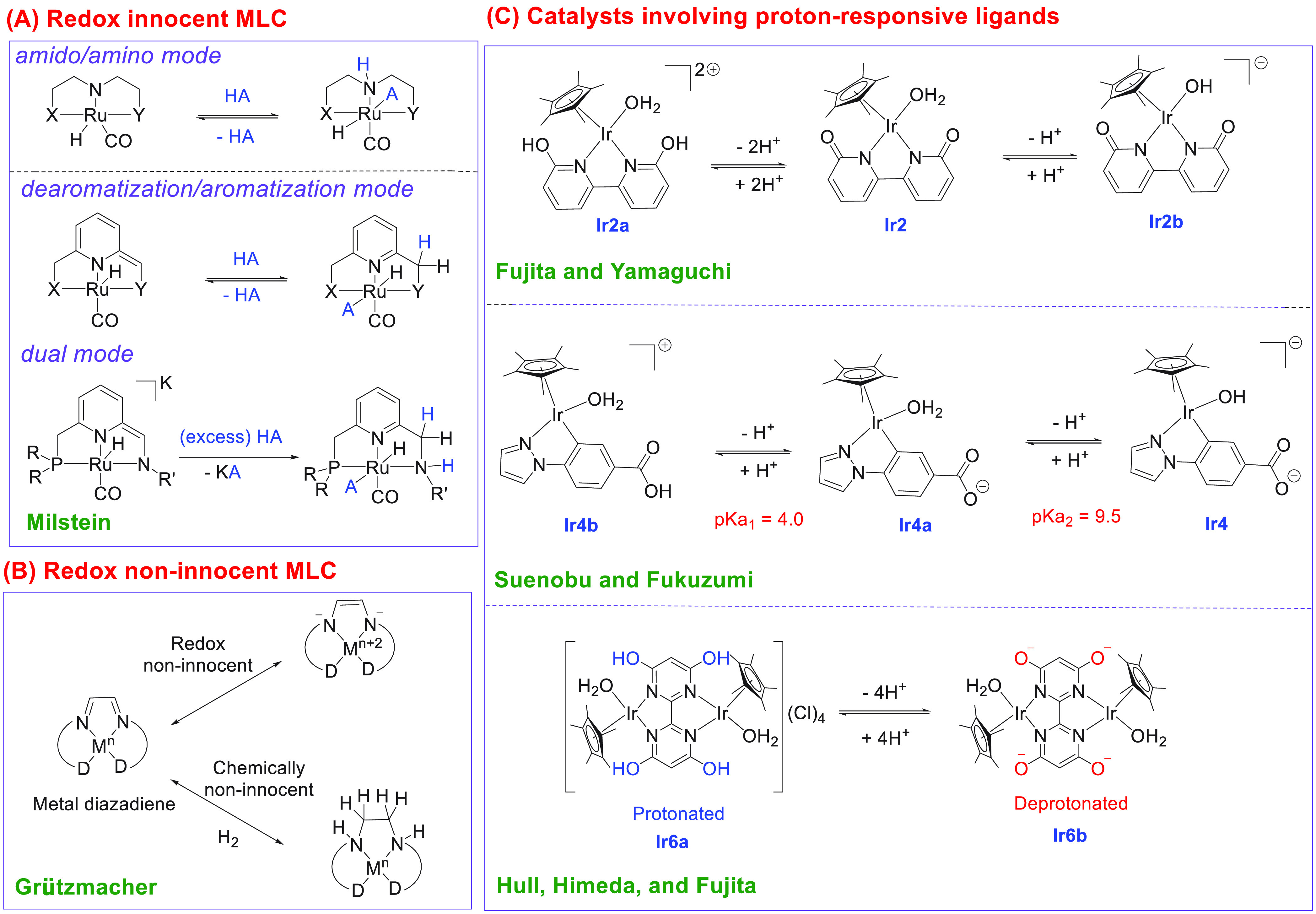
General Classes of Catalysts Discussed in This Review X and Y are neutral ligands, e.g., phosphine or amine derivatives. HA represents bonds such as H–H, H–C, H–O, H–N, H–B, and H–Si. R = ^*t*^Bu, Ph; R′ = benzyl, ^*t*^Bu, and ^*i*^Pr. D = neutral two-electron donor site.

**(a) MLC via amido/amino mode.** Application of MLC via amido/amino mode was first demonstrated by Noyori for catalytic hydrogenation reactions. Since then several examples of transition metal complexes exhibiting MLC via amido/amino have been studied as reviewed in the recent past.^71^ An example of this mode operation is shown in Scheme 1A using a pincer complex containing a MACHO-type ligand. Ruthenium MACHO complexes exhibit tolerance and robustness toward harsh catalytic conditions such as temperature (up to 150 °C), because of which they have been extensively employed for a variety of green homogeneous catalysis, in particular (de)hydrogenative transformations. Examples of such catalysts can be seen throughout this review using complexes of Ru, Ir, Mn, and Fe.

**(b) MLC via dearomatization/aromatization mode.** A new mode of metal–ligand cooperation based on dearomatization and aromatization of pincer complexes was reported by Milstein. As shown in Scheme 1A, such complexes involve pincer ligands containing lutidine backbone and bond activation occurs between the side arm CH proton and the metal center. The dearomatization step is driven by the formation of a stronger bond between the formed amido ligand and metal, whereas the aromatization process is driven by stability gained due to aromatization of the pyridine ring and formation of a more stable coordinatively saturated complex. Extensive investigations by both computation and experiments have been carried to understand the mechanism in detail. A recent article by Khaskin and Gusev reports that the bipyridine complex Ru-BipyPNN can undergo hydrogenation of the bipyridine central ring under reducing conditions.^72^ A review on the application of catalysts exhibiting metal–ligand cooperation via dearomatization/aromatization mode has recently been reported by Fujita.^73^ Another related complex is based on acridine-type PNP ligands where a dearomatized acridine complex is found to be active in catalysis as discussed in this review (e.g., Scheme 24).

**(c) Dual mode of MLC.** The Milstein group has recently reported a new family of pincer complexes capable of exhibiting a dual mode of MLC via an amido/amino mode and a dearomatization/aromatization mode (Scheme 1A).^74^ Such catalysts of ruthenium are highly active for (de)hydrogenative catalysis and can hydrogenate esters and amides^75^ near room temperature. Such catalysts have also been applied for the hydrogenation of commercial resins of nylons and polyurethanes.^76^

**(d) Redox-active MLC.** Grützmacher has developed metal complexes containing the 1,4-bis(5*H*-dibenzo[*a*,*d*]cyclohepten-5-yl)-1,4-diazabuta-1,3-diene ligand that have demonstrated promising activities for the production of H_2_ from aqueous methanol^77^ or formaldehyde^78^ as discussed in this review. Such complexes exhibit both redox noninnocence and chemical noninnocence as shown in Scheme 1B.

**(e) Catalysts based on proton responsive ligands.** Complexes bearing proton responsive ligands that can be deprotonated and protonated on change of pH have been utilized for (de)hydrogenative transformation reactions by multiple research groups (Scheme 1C).^79,80^ The deprotonated and protonated complexes can catalyze different reactions. For example, the deprotonated catalyst can catalyze the hydrogenation of CO_2_ to formate at higher pH whereas the protonated catalyst can catalyze the dehydrogenation of HCOOH at a lower pH.^80^ Such catalysts have been utilized for several (de)hydrogenation reactions such as aqueous methanol reforming (section 2.1.2), dehydrogenation of aqueous formaldehyde (section 2.2), and dehydrogenation of HCOOH and the reverse reaction—hydrogenation of CO_2_ (section 2.4.4).

**(f) Catalysts involving triphos ligands.** Another important class of catalysts employed in (de)hydrogenative catalysis involves triphos ligands (e.g., Scheme 53, *vide infra*). These catalysts are activated using an acid such as HNTf_2_, unlike catalysts exhibiting MLC via the amido/amino or dearomatization/aromatization mode that operate under either basic or neutral conditions. This complements the two types of catalysts to work under different pH conditions and tolerate functional groups of acidic or basic nature.

## 2 Hydrogen Economy

Hydrogen has been long termed as the ideal energy source of the future. Molecular H_2_ is light, is storable, has the highest gravimetric energy content of common fuels (120 MJ kg^–1^), and does not produce any direct emission of common pollutants or greenhouse gases, making it a very attractive candidate as a sustainable and clean energy carrier.^25,81,82^ However, as of now, H_2_ is primarily used as an industrial feedstock for the production of chemicals, e.g., ammonia, methanol, and petroleum refining.^83^ Application of (de)hydrogenation reactions to convert waste to useful chemical resources to enable a circular economy has been recently reviewed.^84^ Cost-effective and sustainable demonstration of H_2_ as a clean energy carrier to manifest the hydrogen economy faces two major challenges: (a) sustainable production of renewable H_2_ and (b) efficient storage of H_2_.

More than 95% of H_2_ is currently produced from fossil fuel via steam reforming, coal gasification, or the steam methane reforming (SMR) process where natural gas (primarily CH_4_) is reacted with steam to produce CO and H_2_ (CH_4_ + H_2_O → CO + 3H_2_).^85^ The produced CO subsequently reacts with steam to produce more H_2_ and CO_2_; the process is known as the water gas shift reaction (CO + H_2_O → CO_2_ + H_2_).^86^ Thus, one molecule of CO_2_ is produced per four molecules of H_2_ produced via the SMR process. This makes the release of 5.5 tons of CO_2_ in the atmosphere per ton of H_2_ produced. It is possible to capture almost 70% of the released CO_2_ and store it in deep underground wells (carbon capture and sequestration);^87^ however, such operations are not in common practice. Thus, the production of H_2_ from SMR is not ideal for the hydrogen economy as the feedstock to produce H_2_ is a fossil fuel and a significant amount of CO_2_ is emitted to the atmosphere in this process. Therefore, the development of alternative technologies for the cleaner production of renewable H_2_ is crucial for the hydrogen economy. Several alternative technologies for the production of clean and renewable H_2_ have been evaluated. A technology that is being tested on a large scale for the clean production of H_2_ is the pyrolysis of methane,^88^ where methane is bubbled on a molten catalyst at high temperature (∼1000 °C). The reaction (CH_4_ → C + 2H_2_; Δ*H*° = 74 kJ/mol) requires 5 kWh of electricity for process heat to produce 1 kg of H_2_. The process is claimed to generate no pollution as the produced carbon can be used as manufacturing feedstock in industry or be landfilled. Along a similar direction, the Kværner process^89^ has been developed by the Norwegian engineering firm Kværner that produces H_2_ from hydrocarbons (e.g., natural gas and biogas) in a plasma burner at 1600 °C (C_*n*_H_*m*_ → *n*C + *m*/2H_2_). Although the production of H_2_ from methane pyrolysis and the Kværner process are clean as they do not produce any greenhouse gas, the produced H_2_ is nonrenewable due to the use of fossil fuel feedstock. Biomass has been investigated as an attractive feedstock to produce renewable H_2_ via the process of pyrolysis (biomass + heat → CH_4_ + CO + CO_2_ + H_2_ + other products) or gasification (biomass + O_2_ or H_2_O + heat → H_2_O + CO + CO_2_ + CH_4_ + H_2_ + other products).^90^ However, these processes produce CO_2_, making them less ideal for the vision of hydrogen economy. The most promising approach for the clean production of renewable H_2_ is the electrolysis of water. However, water electrolysis is thermodynamically unfavorable (H_2_O → H_2_ + 1/2O_2_; Δ*G*° = 237.24 kJ/mol; Δ*H*° = 285.83 kJ/mol) and requires a substantial energy input (50–55 kWh of electricity/kg of H_2_).^91^ This makes the success of the technology dependent on the cost and nature of the electricity (e.g., renewable or nonrenewable) to be used for the process. Several renewable energy sources, e.g., wind, solar, and geothermal energy, have been evaluated or demonstrated for the large-scale electrolysis of water. In 2019, only 0.1% of the global hydrogen was produced via the electrolysis of water.^92^ A brief report on the production of H_2_ from biomass and water electrolysis using homogeneous transition metal catalysts is discussed in section 2.5.

Another major challenge lying in front of the hydrogen economy is hydrogen storage as H_2_ has a very low volumetric energy density (0.0108 MJ L^–1^), making it almost impossible to use in its normal form under mild conditions of pressure and temperature for several sectors such as transport. Conventionally, hydrogen gas is stored physically in compressed form at very high pressure (100–700 bar) or in the cryogenic form at a very low temperature of −253 °C. Both the processes are highly energy intensive and not economical, especially for long-term or long-distance transport. Thus, the safe and economical storage of hydrogen gas for long-term and long-distance transport is an important challenge in front of the hydrogen economy. In recent years, there has been significant development toward different approaches for hydrogen storage, in particular, by physisorption in porous materials or by making or breaking chemical bonds. Several reviews have been reported discussing the advantages and disadvantages of each technique.^93−97^ Several properties need to be considered for a suitable hydrogen storage material, for example, the following properties.

**(a) Gravimetric storage capacity.** The material should have a high gravimetric hydrogen storage capacity (e.g., >5.5 wt %, U.S. Department of Energy target).^98^

**(b) Viscosity.** Lower viscosity would allow smoother transport of the carrier materials through various parts of the reactor/storage system, thus making a better hydrogen storage material.

**(c) Gas stream purity.** The presence of contaminants such as CO or NH_3_ in the produced hydrogen gas stream could poison catalysts used in the fuel cells and reduce the efficiency of energy production. Thus, materials or processes producing contaminant-free hydrogen gas are highly desirable.

**(d) Reversibility.** For the hydrogen storage process to be sustainable, both the discharge of H_2_ from the charged fuel and the regeneration of the charged fuel from spent fuel need to be feasible in a green and cost-effective way. Thus, the thermodynamics of both the dehydrogenation and regeneration processes need to be considered for the purpose of hydrogen storage.

**(e) Stability.** Both the charged fuel and spent fuel should be thermally/photolytically stable to keep the charging/discharging cycle continuing.

**(f) Toxicity.** The material should be of low toxicity.

**(g) Availability.** The material should be inexpensive and abundant. Ideally, it should be compatible with the existing infrastructure.

H_2_ gas can be stored through physical adsorption on various materials. However, in almost all cases either a very low temperature or a high pressure is required for the storage. Another area of hydrogen storage is based on chemical hydrogen storage materials where hydrogen atoms are covalently bound, and the H_2_ can be produced by a thermal or catalytic process. Several solid carriers such as metal hydrides,^99,100^ borohydrides,^101^ alanates,^102^ and imides/amides^103^ have been investigated. A majority of them suffer from issues of low hydrogen storage capacity and difficulty in the regeneration of the charged fuel from the spent fuel. Another mode of hydrogen storage is based on liquid organic hydrogen carriers (LOHCs). Effective release of H_2_ from the charged fuel carrier and its selective regeneration under mild conditions require a suitable catalyst. In this section, we review how homogeneous catalysis has contributed to the discoveries of new and potential chemical hydrogen storage materials.

### 2.1 Methanol as a Hydrogen Storage Material

Methanol is an inexpensive alcohol, is a stable liquid with low viscosity (∼0.54 mPa·s) under ambient conditions, and has a high theoretical gravimetric hydrogen capacity (up to 12.5 wt %), making it a promising candidate as a hydrogen storage material. Dating back to the period of 1985–1996, the groups of Saito,^104−108^ Cole-Hamilton,^109^ Shinoda,^110^ and Maitlis^111^ reported on the production of hydrogen gas from methanol, where homogeneous catalysts based on ruthenium, rhodium, or iridium were employed for the dehydrogenation of anhydrous methanol either thermally or photochemically. Depending on the catalyst and the reaction conditions, different products, e.g., formaldehyde, formate salt, methylal (formaldehyde dimethyl acetal), and methyl formate were obtained, influencing the yield of hydrogen gas. Table 1 shows the thermodynamic parameters of different products formed from the dehydrogenation of methanol. Dehydrogenation of methanol to CO and H_2_ offers a possibility of high hydrogen storage capacity (12.5 wt %), but the presence of CO is poisonous to fuel cells (entry 1, Table 1). On the other hand, the formation of dimethoxymethane or methylal offers a very low hydrogen storage capacity (2.1 wt %). For the purpose of hydrogen storage, dehydrogenation of aqueous methanol, named “aqueous methanol reforming”, is attractive as it leads to the complete dehydrogenation of methanol to inert CO_2_ (does not poison the fuel cell catalyst), offering a high gravimetric hydrogen storage capacity (12.0 wt %, entry 3, Table 1). More importantly, several examples for the direct or indirect hydrogenation of CO_2_ to methanol have been reported (*vide infra*, section 4) making methanol a material suitable for the reversible storage of H_2_. A few reviews have been reported for the production of H_2_ from methanol.^112−114^ Here, we focus on the aqueous methanol reforming processes catalyzed by homogeneous transition metal complexes for the purpose of hydrogen storage.

**Table 1:** Thermodynamic Parameters and Theoretical Hydrogen Storage Capacity for the Dehydrogenation of Methanol^114^

entry	reaction	Δ*H*° (kJ mol^–1^)	Δ*S*° (J mol^–1^ K^–1^)	Δ*G*° (kJ mol^–1^)	theoretical hydrogen storage capacity (wt %)
1	CH_3_OH(l) → CO(g) + 2H_2_(g)	+127.9	+332	+29.0	12.5
2	CH_3_OH(l) → HCHO(g) + H_2_(g)	+129.8	+222	+63.5	6.2
3	CH_3_OH(l) + H_2_O(l) → 3H_2_(g) + CO_2_(g)	+130.7	+408.7	+8.9	12.0
4	CH_3_OH(g) + H_2_O(g) → 3H_2_(g) + CO_2_(g)	+53.3	+176.8	+0.6	12.0

#### 2.1.1 Aqueous Methanol Reforming Using Ruthenium-Based Catalysts

**Scheme 2:**
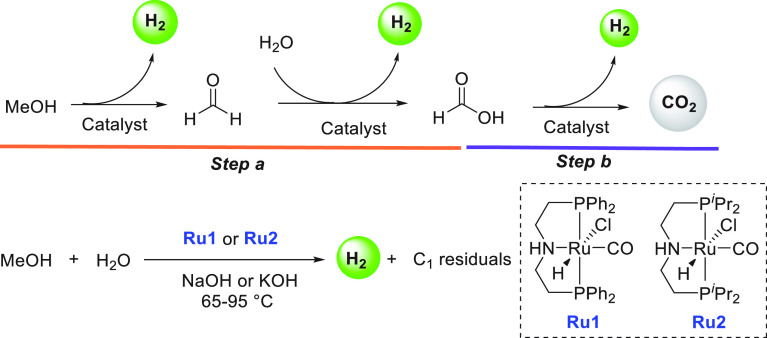
Aqueous Methanol Reforming Using Ruthenium Pincer Catalysts

Major breakthroughs in the aqueous methanol reforming process under mild catalytic conditions were reported in 2013 by the groups of Beller^115^ and Grützmacher.^77^ Beller utilized ruthenium-MACHO pincer complexes (**Ru1**, and **Ru2**) for the dehydrogenation of MeOH/H_2_O mixture to CO_2_ (or CO_3_^2–^) and H_2_ (Scheme 2).^115^ In the presence of complex **Ru1** or **Ru2** and base, methanol is first dehydrogenated to produce formaldehyde, which in the presence of water is dehydrogenated to formic acid, and finally formic acid is dehydrogenated to produce CO_2_. The overall process produces 3 equiv of hydrogen gas (CH_3_OH + H_2_O = 3H_2_ + CO_2_) at 65–95 °C, significantly lower than the temperature of heterogeneously catalyzed aqueous methanol reforming processes (>200 °C).^125^ Catalytic conditions were optimized by varying the catalyst, concentration of base, methanol–water ratio, and reaction temperature. Significant catalytic activity was observed using the conditions of 8 M KOH, reaction temperature of 91 °C, and a 9:1 MeOH/H_2_O solution, where **Ru1** (19 ppm) demonstrated a TOF of 1023 h^–1^, whereas **Ru2** (19 ppm) demonstrated even a higher turnover frequency (TOF) of 2668 h^–1^. Hydrogen gas was detected in highly pure form; contaminants such as CO and CH_4_ were detected in very small amounts (<1 ppm), lower than those reported using heterogeneous catalysts.

**Scheme 3:**
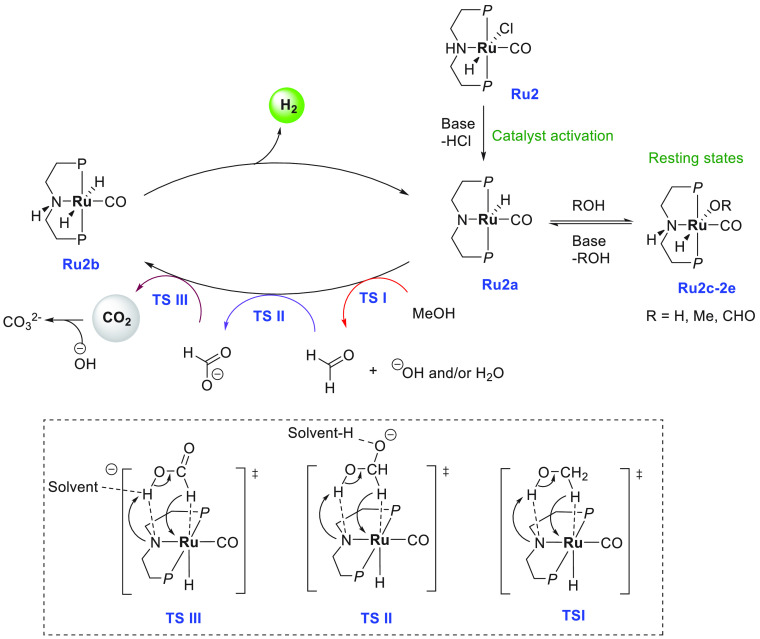
Proposed Mechanism for Aqueous Methanol Reforming Catalyzed by a Ruthenium Pincer Complex (*P* = P^*i*^Pr_2_) and Probable Transition States

A proposed mechanism for the aqueous phase dehydrogenation of methanol has been outlined in Scheme 3. The first step is the reaction of the precatalyst **Ru2** with a base to produce the active species ruthenium amido complex **Ru2a**. Complex **Ru2a** can dehydrogenate methanol via an “outer-sphere” concerted process to form HCHO and complex **Ru2b** via a transition state **TSI**, although HCHO was not detected in solution. Release of H_2_ from **Ru2b** can regenerate complex **Ru2a** which can coordinate with HCHO. Attack of hydroxide on the coordinated aldehyde forms a *gem*-diolate complex which is presumably stabilized by solvent (MeOH/H_2_O) as shown in the transition state **TSII**. Further elimination of H_2_ from the *gem*-diolate complex leads to the formation of a formate species, which can either decoordinate to regenerate the active species **Ru2a** or release CO_2_ to produce complex **Ru2b** via the transition state **TSIII**. A third equivalent of H_2_ is released from **Ru2b** via metal–ligand cooperation, regenerating the active species **Ru2a**. Notably, hydride species **Ru2c**, **Ru2d**, and **Ru2e** were observed in solution, suggesting them to be the resting states. More detailed mechanisms elaborating the role of the base, solvent, and metal–ligand cooperation have been reported by several groups in the recent past.^126−131^

**Scheme 4:**
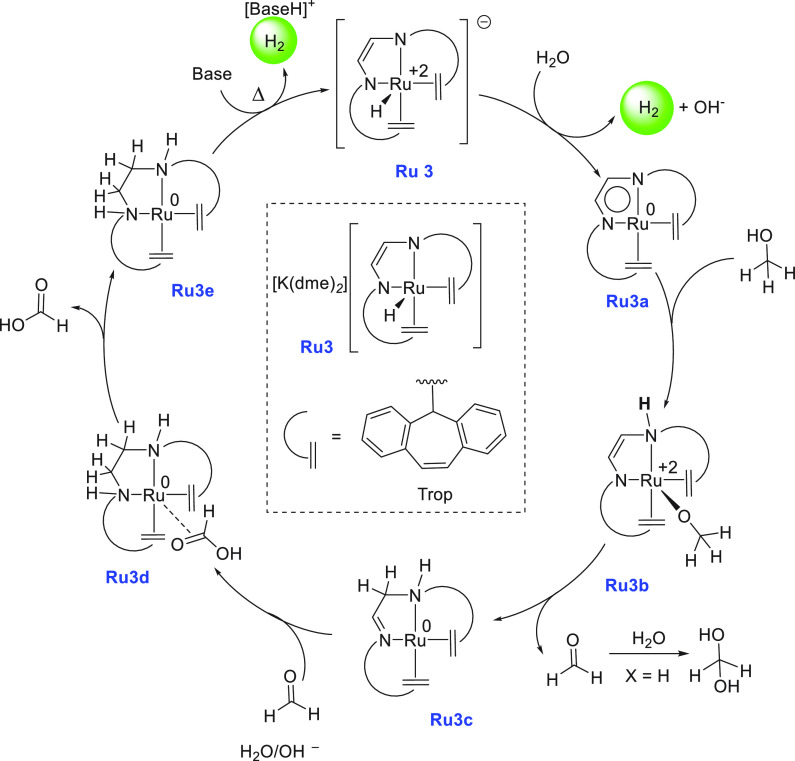
Proposed Mechanism for the Dehydrogenation of Aqueous Methanol Using the Ruthenium Complex **Ru3**

Around the same time, an anionic ruthenium complex **Ru3** was reported by Grützmacher for the dehydrogenation of CH_3_OH/H_2_O mixture to CO_2_/H_2_. Using 0.5 mol % **Ru3**, 80% conversion of methanol was obtained in 10 h at 90 °C (THF solvent) under neutral conditions (TOF, 54 h^–1^).^77^ Compared to Beller’s catalyst discussed above, Grützmacher’s catalyst exhibited a lower TOF but a higher methanol conversion. More remarkably, catalysis was achieved under a neutral condition without needing any base or additive. Furthermore, the practicality of the system was demonstrated by feeding the evolved 3:1 H_2_/CO_2_ gas mixture directly to power an H_2_/O_2_ fuel cell. Based on the experimental studies, a mechanism involving the noninnocence of the trop_2_dad ligand was proposed (Scheme 4). The catalysis starts with the reaction of the anionic [Ru(H)(trop_2_dad)] (**Ru3**) with H_2_O to form complex **Ru3a** with the elimination of 1 equiv of H_2_. This is followed by O–H activation of CH_3_OH to form **Ru3b** and subsequent C–H activation of the coordinated methoxide to form **Ru3c** and 1 equiv of aldehyde. The formed aldehyde then reacts immediately with water to form methanediol. The amino imine complex **Ru3c** can dehydrogenate methanediol to generate formic acid, forming complex **Ru3e** via **Ru3d** and releasing HCOOH. Finally, the Ru(0) complex **Ru3e** reacts with a base and eliminates H_2_ to regenerate the active species **Ru3**. HCOOH can finally be dehydrogenated by complex **Ru3a** to produce CO_2_. More detailed mechanisms have been reported by the groups of Yang,^132^ Hall,^133^ and de Bruin/Grützmacher^134^ using DFT calculations.

**Scheme 5:**
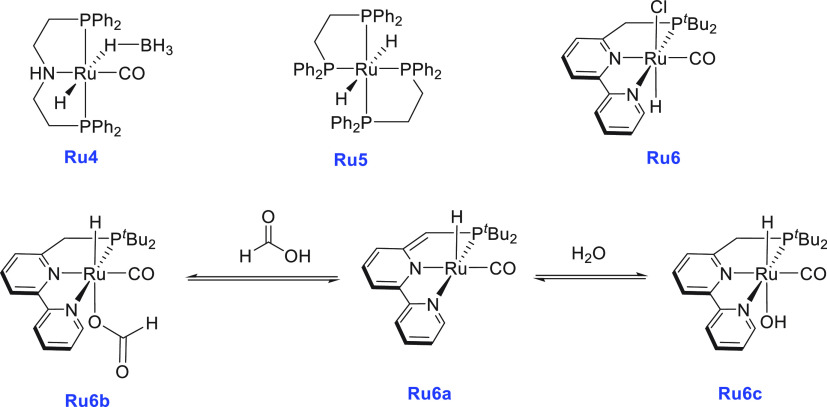
Structures of Ruthenium Complexes **Ru4**, **Ru5**, and **Ru6** and Equilibrium among Complexes **Ru6a**, **Ru6b**, and **Ru6c**

Around the same time, Beller also reported aqueous methanol reforming under neutral conditions using a dual catalytic system—a ruthenium-MACHO pincer complex **Ru4** and a ruthenium bisphosphine dihydride complex **Ru5** (Scheme 5).^116^ The Ru-MACHO-BH complex (**Ru4**) under a low catalytic loading (e.g., 5 μmol) was found to be inactive for the dehydrogenation of methanol/water (methanol, 9.0 mL; water, 1.0 mL) in the absence of a base. However, under a higher catalytic loading (95 μmol of **Ru4**), a dehydrogenation reaction was observed, and the gas evolution rate was found to reach 61 mL/h. The requirement of higher catalytic loading was attributed to the enhanced rate of decomposition of formic acid as the acid could poison the catalyst by strongly binding to the vacant ruthenium site. To overcome this challenge, a dual-catalytic system was used where the role of Ru-MACHO-BH catalyst **Ru4** was to convert methanol to formic acid (step a in Scheme 2) and the decomposition of formic acid was performed by Ru(H)_2_(dppe)_2_, **Ru5** (Scheme 5). A significant catalytic activity (TOF_3 h_ = 138 h^–1^) was obtained in the presence of catalysts **Ru4** (5 μmol) and **Ru5** (5 μmol), and hydrogen gas at the rate of 60 mL/h was collected from a methanol (9.0 mL)/water (1.0 mL). Notably, less than 8 ppm of CO gas was detected by GC. Comparative experimental studies revealed that although both catalysts **Ru4** and **Ru5** were capable of performing both steps independently (Scheme 2)—conversion of methanol to HCOOH (step a) and decomposition of HCOOH to CO_2_ and H_2_ (step b)—when used together they exhibited a positive synergistic effect and resulted in higher catalytic activity.

Soon after, Milstein reported the ruthenium PNN catalyst **Ru6** (Scheme 5) for the dehydrogenation of a methanol/water solution.^118^ At 100 °C, using 0.025 mol % complex **Ru6**, about 80% yield of H_2_ gas was obtained from the basic (KOH, 2 equiv) solution of MeOH/H_2_O in toluene. No dehydrogenation was observed under neat conditions, without adding an external solvent such as THF or toluene which was proposed to solubilize the catalytically active species. A quantitative amount of hydrogen gas was produced at 115 °C in 24 h from formic acid (1 mL, 2.65 mmol) in the presence of 2 equiv of KOH using complex **Ru6** (0.09 mol %) and KO^*t*^Bu (1.1 mol %). However, in the absence of a base (KOH), only a 25% yield of hydrogen gas was obtained. Mechanistic studies revealed that the precatalyst **Ru6** in the presence of a base forms the dearomatized complex **Ru6a** which can react with H_2_O to form a hydroxy complex, **Ru6c**, or with HCOOH to form a formate complex, **Ru6b** (Scheme 5). H_2_O was found to be detrimental for the decomposition of the HCOOH step, and therefore a higher catalytic activity in toluene, in which a high concentration of the catalyst and very low water concentration are expected, was observed. On the basis of this observation, an equilibrium among complexes **Ru6**, **Ru6a**, and **Ru6b** as depicted in Scheme 5 was suggested. The catalyst was found to be remarkably reusable and showed no noticeable loss in activity for nearly 1 month under the conditions described above, and ∼1.53 g of methanol (MeOH was added on regular intervals without isolation or purification of the catalyst) was fully converted to H_2_ and CO_2_ by using 0.0024 g of catalyst **Ru6** exhibiting a turnover number (TON) of ∼29 000.

In 2016, Reek reported aqueous methanol reforming using a ruthenium complex **(Ru7**, Table 2, entry 10) under basic conditions to produce H_2_, formate, and carbonate.^121^ Complex **Ru7** (12 μmol) in the presence of 8 M KOH catalyzed the dehydrogenation of a methanol:water solution (9:1 v/v) in dioxane (total volume 30 mL), exhibiting a TOF of 55 h^–1^ at 82 °C for 4.5 h.

**Table 2:**
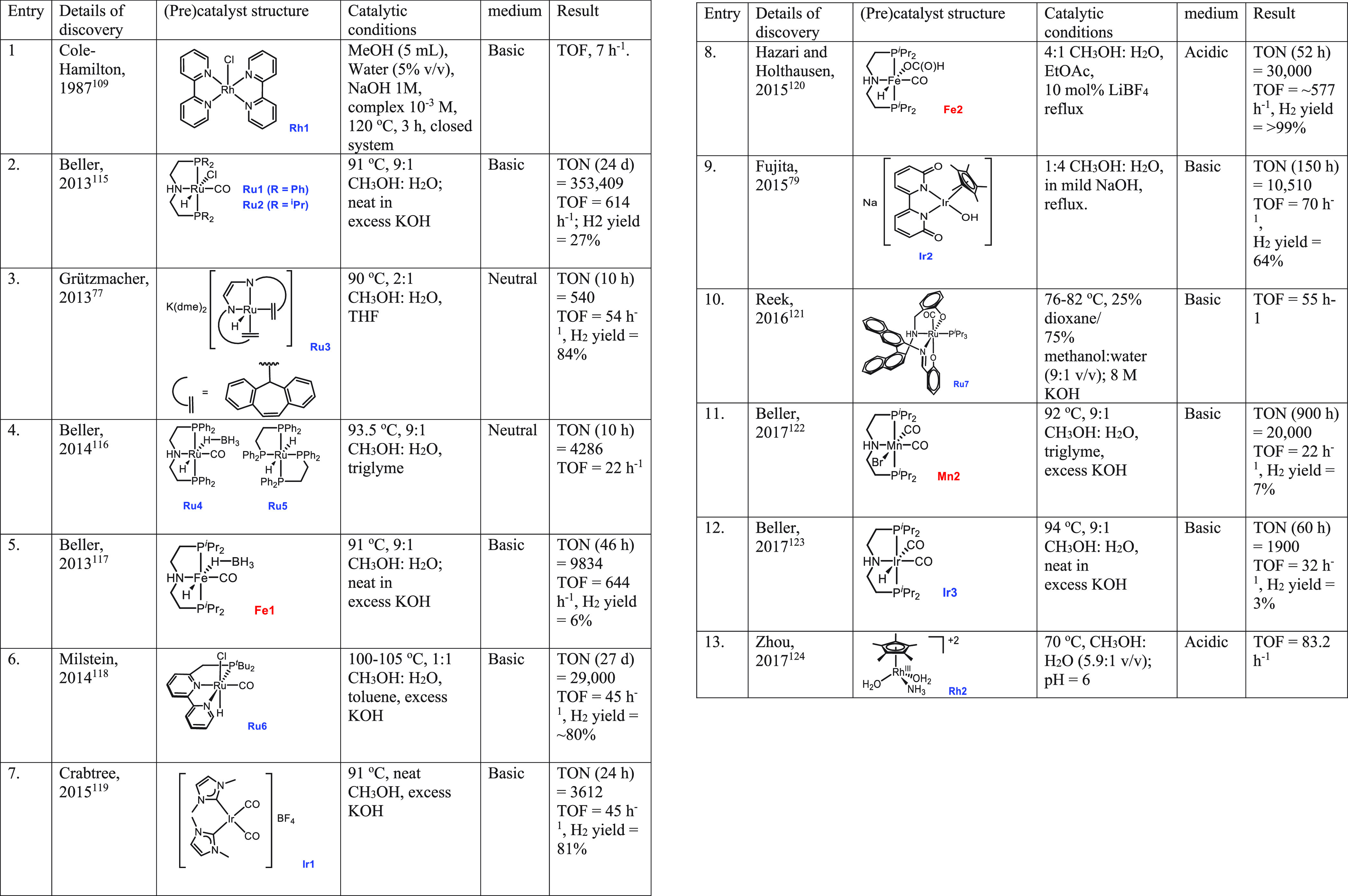
Details of Homogeneous Catalysts Reported for Aqueous Methanol Reforming

#### 2.1.2 Aqueous Methanol Reforming Using Iridium and Rhodium-Based Catalysts

**Scheme 6:**
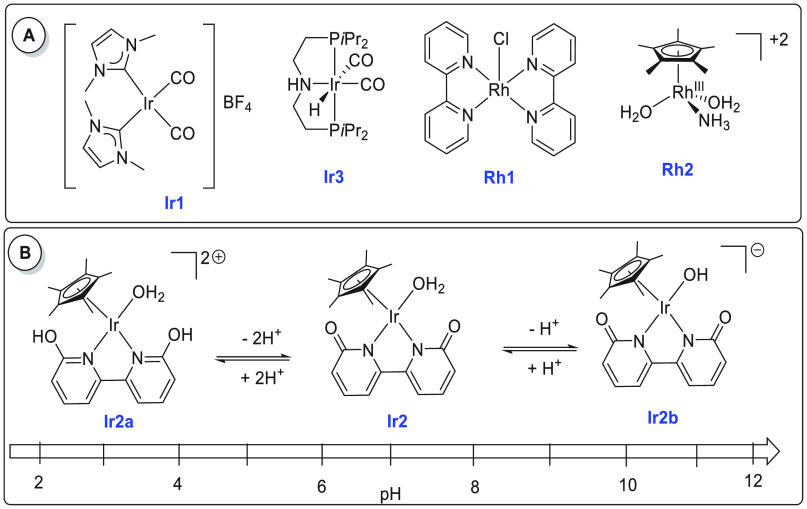
(A) Structures of Iridium and Rhodium Complexes Discussed in Section 2.1.2 and (B) Equilibria among Complexes **Ir2**, **Ir2a**, and **Ir2b**

An iridium-based catalyst for methanol dehydrogenation was reported by Crabtree in 2015.^119^ The iridium complex **Ir1** (Scheme 6A) catalyzed the dehydrogenation of dry methanol under basic conditions (6.7 M KOH) exhibiting a TON of 3612 in 24 h at 91 °C and 81% yield of hydrogen. Unlike previous systems, this process predominantly formed formate rather than CO_2_ or carbonate (<5%). Remarkably, the iridium catalyst **Ir1** was stable in air and no additional water was used in the catalysis. With regard to the aqueous methanol reforming process, Fujita and Yamaguchi reported in 2015 the dehydrogenation of aqueous methanol using iridium complexes **Ir2**, **Ir2a**, and **Ir2b** that could be reversibly interconverted in water by changing the pH of the solution (Scheme 6B).^79^ Addition of NaOH to the aqueous solution of **Ir2a** resulted in the formation of complex **Ir2** at pH 6.8. Further addition of NaOH produced complex **Ir2b** at pH 12. The reverse process was demonstrated by the addition of triflic acid (HOTf) to the solution of **Ir2b** producing complex **Ir2** at pH 6.8 and subsequently complex **Ir2a** at pH 2.7. The catalytic aqueous methanol reforming reaction was found to work under basic conditions suggestive of the requirement of a basic medium for the generation of the active species. Indeed, in the presence of the complex **Ir2a** (0.5 mol %) and NaOH (0.5 mol %), 84% yield of hydrogen gas was obtained from a methanol/water (1:4) solution under reflux conditions for 20 h. A continuous supply of base was needed as the generated CO_2_ dissolves in the reaction medium and lowers the pH, transforming the active species **Ir2b** to the inactive species **Ir2a**. As the need for a continuous supply of a base to keep the catalyst active is a bottleneck for the commercialization of this technology, Inagaki, Fujita, and co-workers have recently reported steam reforming of methanol using vapor-phase flow technology.^135^ The modified anionic iridium bipyridonate (Ir-bpyd) complex (**Ir2b**) was immobilized on a periodic mesoporous organosilica to make a heterogeneous catalyst that could catalyze the dehydrogenation of a methanol/water mixture in the vapor-phase reaction without needing a base. The use of vapor-phase reaction methodology was attributed to prevent CO_2_ to neutralize the anionic iridium bipyridonate complex keeping the catalytic center active. Detailed studies on the acid–base equilibrium of iridium complexes containing dihydroxy-bipyridine ligands were reported by Himeda and Fujita.^136^ A separate study by the same group revealed that the position of the hydroxy group in the bipyridine also plays a crucial role in the substrate activation process and in the catalytic outcome.^137^

In a similar direction, Beller, in 2017 reported an iridium-PNP-MACHO pincer complex **Ir3** (Scheme 6A) for the catalytic dehydrogenation of aqueous methanol under basic conditions.^123^ The catalytic activity of the iridium pincer complex was also compared with the analogous ruthenium complex **Ru2** (Table 2, entry 2) and iron complex **Fe1** (Table 2, entry 5) and was found to be significantly lower. The base concentration was found to play a significant role in catalytic activity. For example, using 0.5 M KOH and 4.18 μmol of catalyst **Ir3** resulted in a TON of 1400 for the dehydrogenation of MeOH/H_2_O solution (9:1, v/v) at 94 °C; however, in the presence of 8.0 M KOH a TON of 1900 was achieved and the catalyst was stable for up to 60 h. In the presence of an excess of base, CO_2_ was trapped as carbonate and only traces of CO_2_ were detected by GC.

Other than ruthenium and iridium, a couple of rhodium complexes have also been utilized for the aqueous methanol reforming process. Seminal work by Cole-Hamilton in 1987 revealed that aqueous methanol, albeit in a lower yield, can be dehydrogenated using the [Rh(2,2′-bipyridyl)_2_]Cl complex **Rh1** (Scheme 6A).^109^ Despite the seminal report, rhodium complexes were not studied for the aqueous reforming of methanol for a long time. Thirty years later, in 2017, Zhou reported the catalytic activities of several rhodium complexes for aqueous methanol reforming at 70 °C without adding any external base, out of which [Cp*Rh(NH_3_)(H_2_O)_2_]^2+^**Rh2** (Scheme 6A) was found to be the most active.^124^ A slight acidic pH of the solution improved the catalytic activity, and the highest TOF of 83 h^–1^ was observed at pH 6 using a phosphate buffer.

#### 2.1.3 Aqueous Methanol Reforming Using Base-Metal Catalysts

**Scheme 7:**
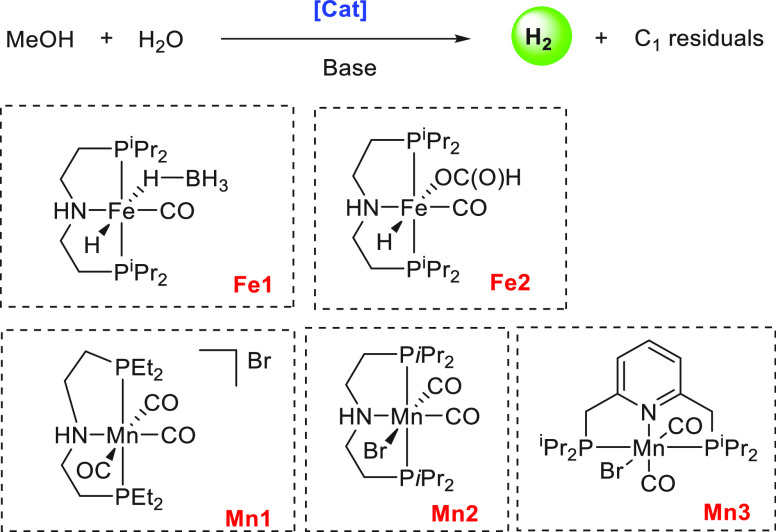
Manganese and Iron Catalysts for Aqueous Reforming of Methanol

Although precious metals have been at the forefront of catalysis, their low abundance and high cost raise the concern of sustainability and create the need for the development of base-metal catalysts. Lately, there has been a substantial development in the direction of homogeneous catalysts based on complexes of earth-abundant metals for (de)hydrogenation reactions which have been reported in several recent reviews.^138−141^ In the direction of aqueous methanol reforming, the first homogeneous catalyst of an earth-abundant metal, the iron pincer complex **Fe1**, was reported by Beller in 2013.^117^ However, the iron complex **Fe1** (Scheme 7) was not as stable as the analogous ruthenium complex **Ru2** (Table 2, entry 2) discussed earlier, and the catalytic activity was found to diminish over time. An outer-sphere mechanism analogous to that discussed for the ruthenium pincer complex (*vide supra*, Scheme 3) involving metal–ligand cooperation was proposed.

In 2015, Bernskoetter, Hazari, and Holthausen reported a highly active iron catalyst, **Fe2** (Scheme 7), for aqueous methanol reforming under acidic conditions.^120^ This is unlike most of the homogeneous catalysts reported earlier that required a basic medium. The Fe-PNP pincer catalyst **Fe2** (Scheme 7) in the presence of a Lewis acid cocatalyst (LiBF_4_) exhibited a TON up to 51 000 for the production of H_2_/CO_2_ from a 4:1 methanol/water solution. The TON reported here was the highest reported for either the base-metal catalysts or base-free systems. In the absence of water, methanol was dehydrogenatively coupled to form methyl formate. Screening of several Lewis acids revealed that small or oxophilic cations (e.g., Li^+^, Na^+^) and weakly or noncoordinating anions (e.g., PF_6_^–^, BF_4_^–^, and OTf^–^) were the most effective. The use of Lewis acids in a catalytic amount allows the catalysis to occur under milder conditions.

Another base-metal catalyst for aqueous methanol reforming was reported by Beller in 2017 using manganese complexes of PNP-type tridentate ligands.^122^ A series of manganese complexes were evaluated for the dehydrogenation of MeOH/H_2_O (9:1) solution under basic condition (8 M KOH) at 92 °C, out of which **Mn1**, **Mn2**, and a combination of [Mn(CO)_5_Br] and 10 equiv of the ligand HN(CH_2_CH_2_)P(CH(CH_3_)_2_)_2_ (PNP^*i*^Pr ligand) exhibited reasonable catalytic activities of TON_5 h_ = 54, 65, and 68 respectively (Scheme 7). Notably, the catalysis in the case of **Mn1** was found to be highly sensitive to light irradiation and all the catalytic experiments were performed with the exclusion of light. Catalyst **Mn3** was also active but showed lower catalytic activity (TON_5 h_ = 41). Interestingly, the presence of a Lewis acid (LiBF_4_) inhibited the catalysis, unlike the reports by Bernskoetter, Hazari, and Holthausen.^120^ Addition of an excess of the PNP^*i*^Pr ligand to complex **Mn2** resulted in remarkable stability for longer than a month, showing a TON of more than 20 000. The stability of this system was higher than that of the analogous Fe-PNP pincer catalyst **Fe1** (Scheme 7) reported by Beller for aqueous methanol reforming which was stable only for up to 5 days. However, the catalytic activities of the Mn-PNP pincer catalysts were still lower than that of the iron pincer catalyst **Fe2** (Scheme 7) reported by Bernskoetter, Hazari, and Holthausen, which exhibited a TON of up to 51 000. A mechanism similar to that discussed earlier in the case of Ru-PNP catalyst (Scheme 3) was proposed based on the NMR and *ex situ* IR investigations.

### 2.2 Hydrogen Production from Aqueous Formaldehyde/Paraformaldehyde

**Scheme 8:**
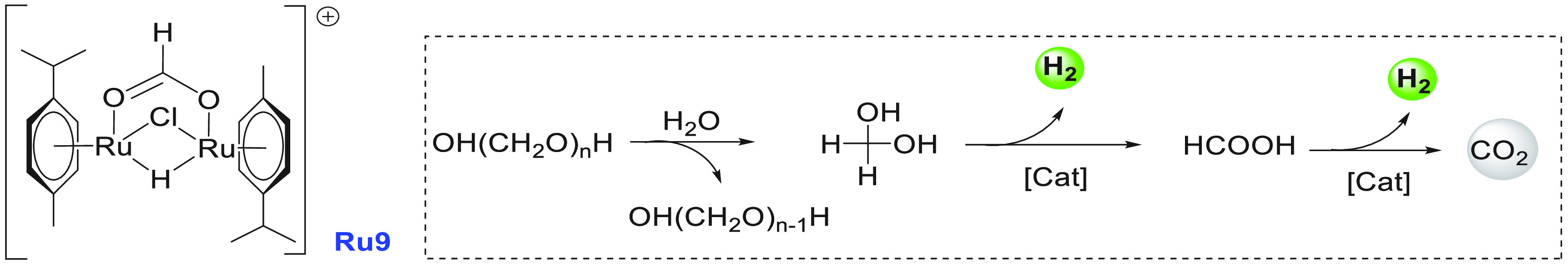
Dehydrogenation of Aqueous Formaldehyde/Paraformaldehyde The structure of **Ru9** is shown on the left.

Similar to aqueous methanol, aqueous formaldehyde also offers the possibility of being used as a hydrogen storage material although with a lower storage capacity. In a typical mechanism, formaldehyde or paraformaldehyde can react with water to form methanediol, which in the presence of a catalyst can liberate H_2_ to form HCOOH. Further catalytic dehydrogenation of HCOOH can liberate H_2_ and CO_2_ (Scheme 8). Overall, the formaldehyde–water system offers a hydrogen storage capacity of 8.4 wt %. Catalytic hydrogenation of CO_2_ to formaldehyde has also been reported, making formaldehyde a potentially reversible hydrogen storage material.^142−146^ However, unlike examples reported for the catalytic dehydrogenation of methanol (section 2.1) and formic acid (section 2.4.4), dehydrogenation of aqueous formaldehyde has received scant attention. In this section, we review homogeneous catalysts that have been utilized for the dehydrogenation of aqueous formaldehyde or paraformaldehyde (Table 3).

**Table 3:**
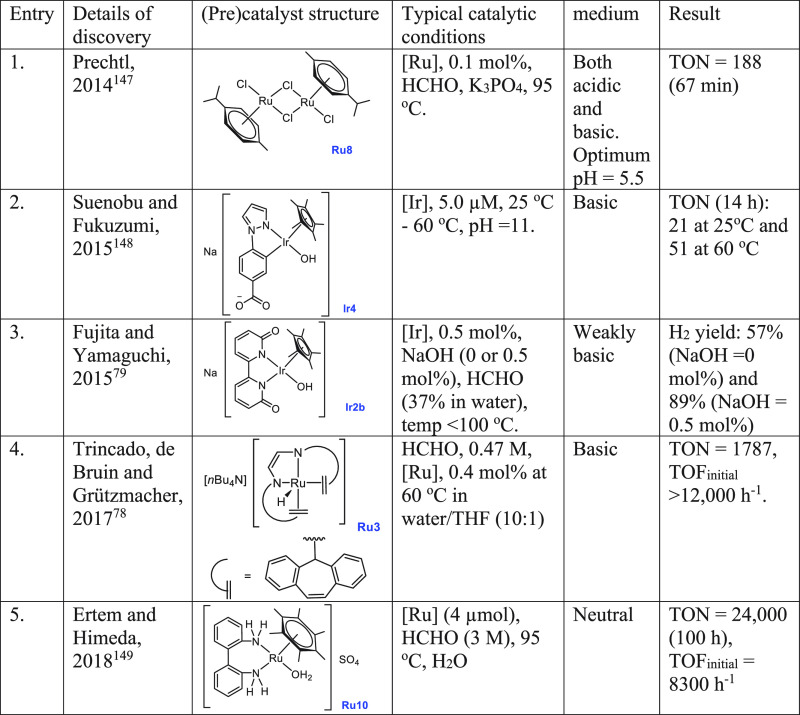
Complexes Used for the Catalytic Dehydrogenation of Aqueous Formaldehyde or Paraformaldehyde

Prechtl, in 2014, published the first report on the aqueous phase reforming of formaldehyde or paraformaldehyde in the presence of the [(Ru(*p*-cymene))_2_(μ-Cl)_2_Cl_2_] complex **Ru8** (Table 3, entry 1).^147^ Interestingly, the dehydrogenation process was also accomplished in the absence of a base (although the presence of a base accelerates the dehydrogenation process slightly), unlike in the cases of dehydrogenation of methanol (*vide supra*, section 2.1) and formic acid (*vide infra*, section 2.4.4) where the presence of a base was found to be crucial for most of the catalytic systems. The efficiency of the catalytic (0.1 mol % **Ru8**) dehydrogenation was found to be dependent on the reaction temperature, and the best catalyst performance was achieved at 95 °C producing H_2_ in 84% yield in 60 min. The use of a pH buffer during catalysis was beneficial but not crucial, and efficient yields of H_2_ were obtained for a wide range of pH, e.g., pH 2.4 (75% H_2_) and pH 9 (73% H_2_) with a maximum yield at pH 5.5 (85% H_2_). Catalyst recyclability and long-term stability were also demonstrated, and continuous production of H_2_ gas was achieved by simply recharging the aqueous phase with paraformaldehyde. The nature of the active catalyst was probed by NMR spectroscopy, mass spectrometry, and isotope-labeling experiments that suggested a dinuclear ruthenium species [(Ru(μ-cymene))_2_(μ-H)(μ-HCO_2_)μ-Cl]^+^ (**Ru9**, Scheme 8) to be the active catalytic species. Moreover, control experiments using metal nanoparticles did not show any catalytic activity, suggestive of homogeneous catalysis. Furthermore, in support of the nature of the active catalyst, complex [(Ru(μ-cymene))_2_(μ-H)(μ-HCO_2_)μ-Cl]BF_4_ (**Ru9**) was independently synthesized and tested in catalysis, resulting in a catalytic activity similar to that of **Ru8** (Table 3, entry 1). On the basis of the mechanistic studies, the mechanism of the catalytic cycle was proposed to be analogous to the one reported by Puddephatt for formic acid dehydrogenation by a binuclear ruthenium complex.^150^

**Scheme 9:**
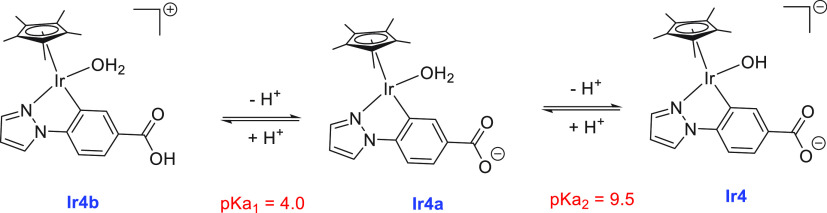
Interconversion of Complexes **Ir4**, **Ir4a**, and **Ir4b** on Changing the pH From ref (148). CC by 3.0.

Deska and Prechtl utilized a similar ruthenium system for the dehydrogenation of formaldehyde and its application for transfer hydrogenation reactions.^151^ Suenobu and Fukuzumi reported in 2015 the decomposition of aqueous paraformaldehyde to H_2_ and CO_2_ using an organoiridium complex **Ir4** that is interconvertible to **Ir4a** and **Ir4b** upon changing the pH as shown in Scheme 9.^148^ At pH 11, a catalytic amount of complex **Ir4b** (5.0 mM) facilitated the decomposition of aqueous paraformaldehyde (2.0 mg, 66.7 mmol) to produce H_2_ and CO_2_ (2:1 molar ratio) at 298 K with a TON of 21 (14 h). Reducing the catalyst concentration to 1.0 mM did not change the TON, whereas a higher TON of 51 was obtained at a higher temperature of 333 K. The rate of H_2_ production was found to decrease upon decreasing pH and no H_2_ production was observed at pH 3, suggesting that the hydroxo form **Ir4** rather than **Ir4a** or **Ir4b** is the actual catalyst.

Similar to **Ir4**, **Ir4a**, and **Ir4b** complexes, Fujita and Yamaguchi utilized the pH-dependent iridium complexes **Ir2**, **Ir2a**, and **Ir2b** discussed earlier for aqueous methanol reforming (Scheme 6), for the dehydrogenation of aqueous formaldehyde.^79^ Complexes **Ir2**, **Ir2a**, and **Ir2b** (0.5 mol %) were tested for the dehydrogenation of formaldehyde–water solution (37% formaldehyde) under reflux conditions for 20 h. Poor catalytic activity was exhibited by complexes **Ir2a** and **Ir2**, but complex **Ir2b** showed higher catalytic activity and 57% hydrogen gas was produced. Interestingly, the catalytic activity was enhanced in the presence of an additional 0.5 mol % NaOH, resulting in an 89% yield of hydrogen gas.

**Scheme 10:**
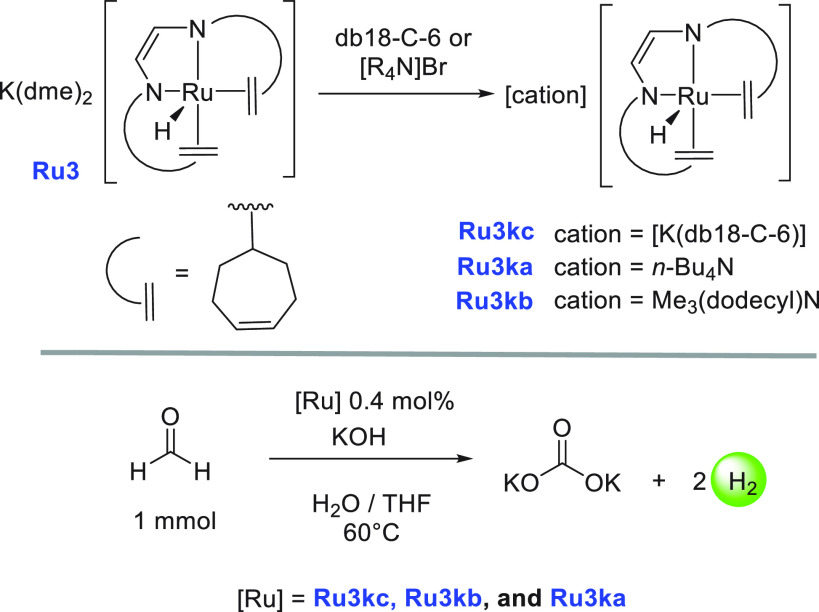
Synthesis of Complexes **Ru3ka**, **Ru3kb**, and **Ru3kc** and Catalytic Dehydrogenation of Aqueous HCHO db18-C-6 = dibenzo-18-crown-6.

In the direction of producing H_2_ gas from formaldehyde, Trincado, de Bruin, and Grützmacher utilized the redox noninnocent complex **Ru3**, used earlier for the dehydrogenation of MeOH/H_2_O mixture (Scheme 4), and analogous complexes for producing H_2_ gas from a formaldehyde–water mixture (Scheme 10).^78^ Interestingly, the dehydrogenation product was trapped as a carbonate salt, unlike the above-discussed examples where CO_2_ gas was eliminated upon dehydrogenation of HCHO. Anionic complexes **Ru3kc**, **Ru3ka**, and **Ru3kb** (0.4 mol %) that exist as a tight ion pair^77^ were screened for the catalytic decomposition of aqueous formaldehyde (1 mmol, initial concentration = 0.47 M) at 60 °C in a water/THF (10:1) mixture (Scheme 10). A low yield of hydrogen gas (32%) and a poor TON (115 in 12 h) were observed in the absence of a base using catalyst **Ru3k**. However, using an additional 6 equiv of KOH (keeping the remaining conditions the same) resulted in the release of 86% hydrogen gas and exhibited a high TON of 430 (in 2 h). The addition of KOH was found to drive the reaction by trapping the evolved CO_2_ as K_2_CO_3_.

**Scheme 11:**
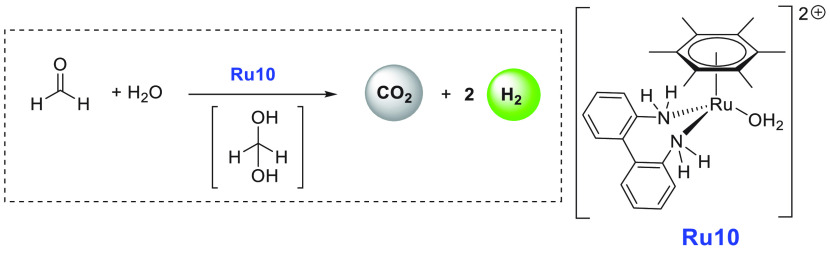
**Ru10** Catalyzed Dehydrogenation of Aqueous HCHO

Although the redox-active complexes (**Ru3ka**, **Ru3kb**, **Ru3kc**, Scheme 10) exhibited high catalytic turnovers and recyclability, the use of an excess base in catalysis limits its commercial application due to the production of a stoichiometric salt waste in the regeneration of formaldehyde. To avoid this problem, Ertem and Himeda, in 2018, reported the ruthenium catalyzed dehydrogenation of aqueous formaldehyde in the absence of a base. Remarkably, a record TON (up to 24 000) was obtained without the use of any additive or a base.^149^ Screening of several ruthenium catalysts (10 μmol) at 50 °C for 7 h revealed that complex **Ru10** (Scheme 11) is the most active catalyst for the dehydrogenation of aqueous formaldehyde. The catalysis was found to be significantly dependent on the temperature, and the optimum temperature was found to be 95 °C. With the use of 10 μmol of catalyst **Ru10**, 5 mmol of paraformaldehyde (in 5 mL of H_2_O) was dehydrogenated at 95 °C to obtain a high yield and selectivity of H_2_ gas (95%) in 7 h.

### 2.3 Amine-boranes as Hydrogen Storage Materials

Amine-boranes (RH_2_N·BH_2_R) are Lewis acid and Lewis base adducts of amines and boranes, where protic and hydridic hydrogen atoms are adjacent to each other, making the release of hydrogen gas kinetically favorable. Low molecular weight amine-boranes can offer very high gravimetric hydrogen capacities; for example, H_3_N·BH_3_ can exhibit a hydrogen storage capacity of up to 19.6 wt %. This has led to immense studies in pursuit of efficient catalysts to dehydrogenate various amine-boranes as discussed below.

#### 2.3.1 Dehydrogenation of Linear Amine-boranes

**Scheme 12:**
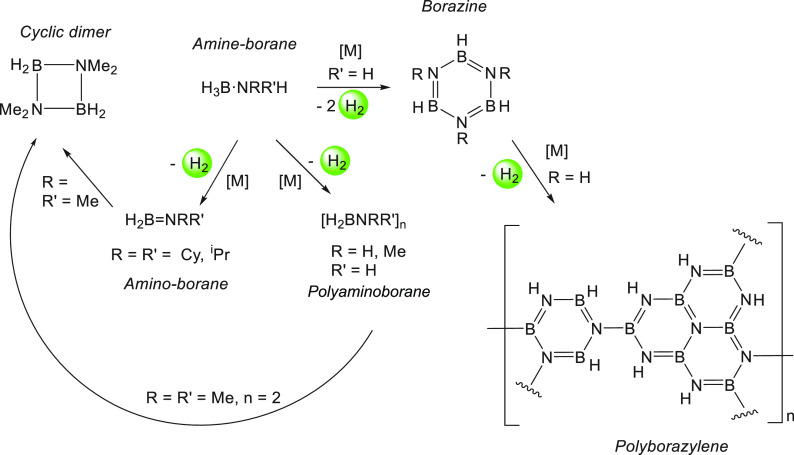
General Pathways for the Dehydrocoupling of Linear Amine-boranes^159^ [M] = metal catalyst.

Dehydrogenation of low molecular weight linear amine-boranes have been intensively studied in the past decade as they exhibit high hydrogen storage capacities and offer a potential route to make new types of “B–N” polymer isosteres with polyolefins. Although amine-boranes can be dehydrogenated thermally without needing a catalyst, the use of a transition metal catalyst can allow the dehydrogenation to occur under milder conditions. Moreover, a catalyst can control the kinetics and influence the final product distribution, which is crucial as it affects the hydrogen storage capacity. Table 4 summarizes the hydrogen storage capacities of various linear amine-boranes and their dehydrogenation products. As described in Table 4 and Scheme 12, H_3_B·NH_3_ can liberate 1, 2, or >2 equiv of hydrogen gas, forming polyaminoborane [H_2_BNH_2_]_*n*_ (6.48 wt %), borazine [HBNH]_3_ (12.96 wt %), or polyborazylene (>12.96 wt %), respectively. A primary amine-borane such as H_3_B·NMeH_2_ can dehydrocouple to form N-methylated borazine [HBNMe]_3_ and liberate 2 equiv of H_2_ gas, offering a high storage capacity of 8.86 wt %. Alternatively, H_3_B·NMeH_2_ can also dehydrocouple to form polyaminoborane [H_2_BNMeH]_*n*_, exhibiting a relatively lower hydrogen storage capacity of less than 4.43 wt %. A secondary amine-borane such as H_3_B·NMe_2_H can release only 1 equiv of hydrogen gas forming the cyclic dimer [H_2_BNMe_2_]_2_ via the amino-borane H_2_B=NMe_2_ intermediate, offering a low hydrogen storage capacity of 3.38 wt % (Scheme 12). However, the products resulting from the dehydrocoupling of H_3_B·NMe_2_H are well-defined and soluble in common organic solvents, unlike H_3_B·NH_3_ that upon dehydrogenation usually results in a mixture of insoluble products. This makes H_3_B·NMe_2_H a strategic choice for mechanistic investigations via kinetics and spectroscopic studies. Indeed, several pioneering studies aimed at elucidating the mechanisms of the catalytic cycle for the dehydrogenation of amine-boranes have been performed using H_3_B·NMe_2_H.^152−158^ Compared to N-substituted amine-boranes, reports on the dehydrogenation of B-substituted amine-boranes are less common. Release of 2 equiv of hydrogen gas from MeH_2_B·NH_2_R (R = H, Me) can produce borazine derivatives [MeBNR]_3_ exhibiting hydrogen storage capacities of 8.86 wt % (R = H) and 6.76 (R = Me) wt %. Properties and catalytic dehydrogenation of these amine-boranes have been reviewed earlier in detail and will not be discussed here.^27−36^

**Table 4:** Hydrogen Storage Capacity for Dehydrogenation of Linear Amine-boranes

entry	dehydrogenation reaction	max hydrogen storage capacity (wt %)
1	*n*H_3_B·NH_3_ → [H_2_BNH_2_]_*n*_ + (*n* – 1)H_2_ (polyaminoborane)	6.48
2	3H_3_B·NH_3_ → [HBNH]_3_ + 6H_2_ (borazine)	12.96
3	*n*H_3_B·NH_3_ → polyborazylene + (>2*n*)H_2_	>12.96
4	3H_3_B·NH_2_Me → [HBNMe]_3_ + 6H_2_ (borazine derivative)	8.86
5	*n*H_3_B·NH_2_Me → [HBNR]*n* + (*n* – 1)H_2_ (polyaminoborane)	<4.43
6	2H_3_B·NHMe_2_ → [H_2_BNMe_2_]_2_ + 2H_2_ (cyclic dimer)	3.38
7	3MeH_2_B·NH_2_Me → [MeBNMe]_3_ + 6H_2_ (borazine derivative)	6.76
8	3MeH_2_B·NH_3_ → [MeBNH]_3_ + 6H_2_ (borazine derivative)	8.86

#### 2.3.2 Dehydrogenation of Cyclic Amine-boranes

**Scheme 13:**
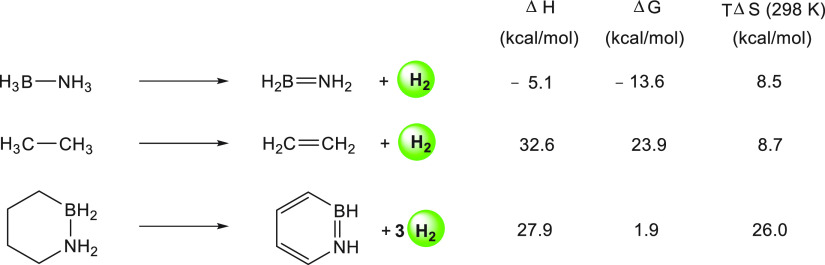
Thermodynamic Parameters for the Dehydrogenation Reactions

Despite the high gravimetric hydrogen capacities of linear amine-boranes and ease of dehydrogenation as reflected by several reports using a range of catalysts, a practical application of amine-boranes for hydrogen storage has not been demonstrated yet. This is mainly because of a high thermodynamic barrier for the regeneration of charged fuel (amine-borane) from the dehydrogenated product (spent fuel). Dixon and Liu proposed that a hybrid system designed by combining both BN and CC fragments might result in a material that can dehydrogenate with a minimum thermodynamic overpotential as the dehydrogenation of an alkane is endergonic whereas the dehydrogenation of amine-boranes is highly exergonic.^160^ For example, the dehydrogenation of H_3_B·NH_3_ is exergonic by −13.6 kcal mol^–1^ while the dehydrogenation of its alkane isostere H_3_C–CH_3_ is endergonic by 23.9 kcal mol^–1^ (Scheme 13). Dixon and Liu also demonstrated that a cyclic amine-borane such as 1,2-BN cyclohexane, containing both BN and CC moieties, leads to a thermodynamically reversible hydrogen storage pathway (taking into account the aromatic stabilization) with Δ*G*(dehydrogenation) = 1.9 kcal mol^–1^ (Scheme 13).^160^

**Scheme 14:**
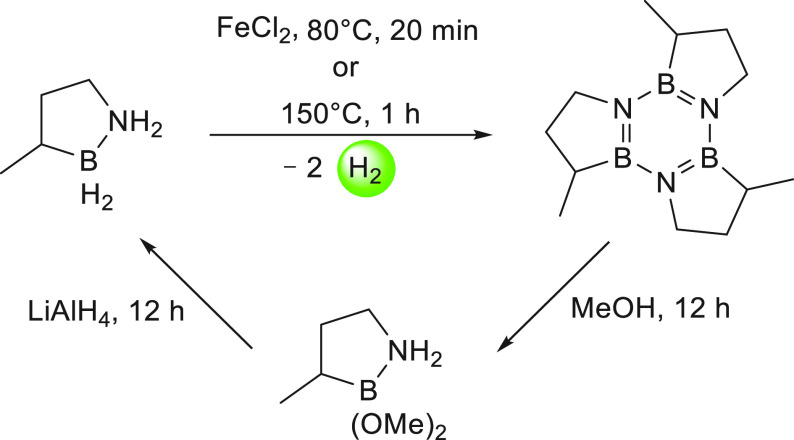
Dehydrogenation and Regeneration of BN-methylcyclopentane

The first example of dehydrogenation of cyclic amine-boranes was reported by Liu in 2011.^161^ Liu reported the synthesis of an air- and moisture-stable amine-borane, BN-methylcyclopentane, and its dehydrogenation under thermal conditions to release 2 equiv of hydrogen gas, forming the tricyclic borazine.^161^ Regeneration of the charged fuel BN-methylcyclopentane from the spent fuel borazine was also demonstrated in an overall yield of 92% using a two-step methodology (Scheme 14). Furthermore, BN-methylcyclopentane was also demonstrated to be a potential candidate for hydrogen storage by evaluating several relevant properties such as thermal stability below 40 °C, a viscosity of 25 cP (comparable to ethylene glycol), and a pure hydrogen stream produced upon thermal dehydrogenation.^162^

**Scheme 15:**
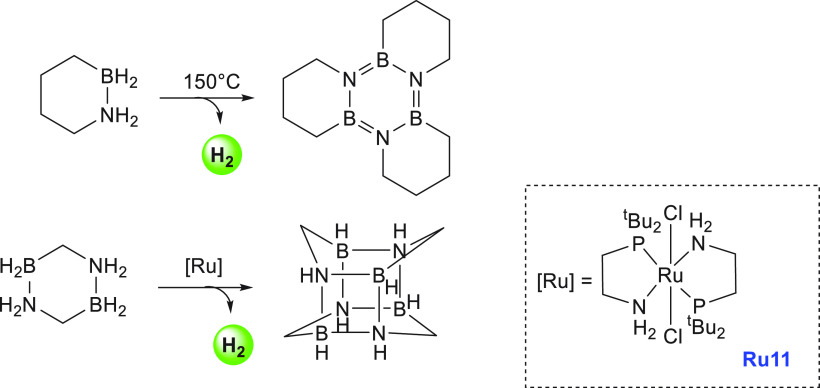
Dehydrocoupling of 1,2-BN Cyclohexane (top) and Bis-BN Cyclohexane (bottom)

Furthermore, in 2011, Liu reported the synthesis and dehydrogenation of 1,2-BN cyclohexane and its thermal dehydrogenation at 150 °C to produce tricyclic borazine (Scheme 15).^163^ Later, in 2016, Weller and Liu utilized iridium and rhodium bis-phosphine complexes to perform dehydrogenation under ambient conditions.^164^ Both BN-methylcyclopentane and 1,2-BN cyclohexane were demonstrated to exhibit a hydrogen storage capacity of 4.7 wt %, although further dehydrogenation of the cyclic backbone can result in a higher hydrogen storage capacity of up to 9.4 wt %. In a similar direction, Liu reported the synthesis of bis-BN cyclohexane and its dehydrogenation to form a cagelike structure catalyzed by the ruthenium-PN complex **Ru11** (0.5 mol %) at 65 °C (Scheme 15).^165^

#### 2.3.3 Regeneration of Amine-boranes from the Spent Fuel

Difficulty in the regeneration of amine-boranes (charged fuel) from the spent “BN” fuel poses a significant challenge in the practical utility of amine-boranes. Multistep processes involving stoichiometric reagents (generating waste) for the regeneration of H_3_B·NH_3_ from the waste BNH_*x*_ products have been demonstrated.^166−169^ Gordon has reviewed regeneration of H_3_B·NH_3_ using a multistep protocol consisting of (a) digestion (H^+^ addition), where a spent fuel (BNH_*x*_) is protonated by a strong acid or a weak acid, e.g., alcohol, amine, or thiol (HX) to produce BX_3_ and NH_3_; (b) reduction (H^–^ addition), where BX_3_ reacts with a reductant, e.g., metal hydride, in the presence of an amine to form H_3_B·NR_3_; and (c) ammoniation, where the reaction of H_3_B·NR_3_ with NH_3_ regenerates H_3_B·NH_3_.^170^ Remarkably, Sutton and Gordon reported in 2011 a one-pot regeneration of H_3_B·NH_3_ from the spent fuel polyborazylene by the reaction of anhydrous N_2_H_4_ in liquid NH_3_.^171^ Polyborazylene (BNH*_x_*) reacted with 1.35 equiv of anhydrous N_2_H_4_ at 40 °C in a sealed vessel for 24 h to regenerate H_3_B·NH_3_ (92% yield) and N_2_ as the byproduct. However, the use of N_2_H_4_, which is a potential hydrogen storage material in itself, for the regeneration process presented a new set of practical challenges in terms of handling hydrazine such as toxicity, instability, and risk of explosion as well as high cost and multistep synthesis of N_2_H_4_ from NH_3_. Interestingly, Manners reported that the B=N bond of a spent fuel, e.g., H_2_B=N^*i*^Pr_2_, can be converted back to the charged fuel H_3_B·N^*i*^Pr_3_ via transfer hydrogenation using amine-boranes, e.g., H_3_B·NHRR′ (R, R′ = H or Me) or linear diborazane Me_3_N·BH_2_·NHMe·BH_3_.^172,173^ Remarkably, the reaction occurred without needing any catalyst at 20 °C (THF solvent) to give yields up to 90%. However, this concept too does not offer a practical sustainable solution as the regeneration process produces spent BN fuel and requires sacrificial charged fuel. Furthermore, Manners reported that aminoboranes H_2_B=NR_2_ can be converted to H_3_B·NHR_3_ in the presence of H_2_O in a single step without needing a catalyst. The reaction was driven by the formation of insoluble borate products (B_*x*_O_*y*_H_*z*_). However, only a low yield of ∼30% was achieved. A higher yield was achieved by the addition of sacrificial agents, e.g., BH_3_·THF or LiBH_4_. Thus, it is essential to develop an efficient and atom-economical route to regenerate amine-boranes from the spent BN fuel. There is no report on the direct hydrogenation of BN spent fuel such as borazine to regenerate H_3_B·NR_3_. As H_3_B·NR_3_ can be easily dehydrogenated at high temperatures, the use of low temperature for the hydrogenation reaction is recommended.^169^ Timoshkin reported that the use of a Lewis acid can significantly decrease the activation energy of hydrogenation.^174^ Szymczak and Heiden have performed DFT calculations and suggested that coordination of borazine to a transition metal fragment such as M(CO)_3_ can decrease the activation energy for the reactivity of borazine toward a hydride source.^175^

### 2.4 Liquid Organic Hydrogen Carriers (LOHCs)

**Figure 1:**
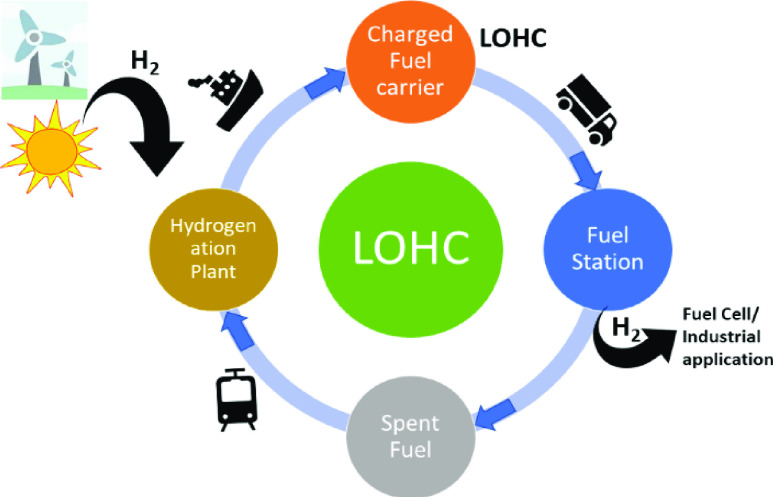
Model depicting use of LOHCs in hydrogen economy.

To overcome the challenges of regenerability and reversibility, liquid organic hydrogen carriers (LOHCs) have been proposed as potential hydrogen materials. LOHCs are low molecular weight organic compounds in the liquid state at room temperature that can liberate hydrogen gas (fuel) in the presence of a catalyst, forming spent fuel that can be converted back to the charged fuel by hydrogenation, thus closing the loop.^176^ The liquid state gives the advantage of utilizing the established infrastructure for delivering gasoline fuels. A model for the utilization of LOHC for the hydrogen economy is shown in Figure 1. As depicted, an LOHC ideally of low flammability and toxicity and in the liquid state, can be easily transported to a fuel station where it can be stored indefinitely under ambient conditions. H_2_ can be produced at the fuel station via catalytic dehydrogenation of the LOHC and can be used for energy generation in a fuel cell or for industrial applications. For transportation purposes, LOHCs can be directly loaded to a hydrogen vehicle where onboard catalytic dehydrogenation can produce H_2_ gas that can be fed to a fuel cell to drive the vehicle. The spent fuel (preferably liquid) produced in the dehydrogenation process can then be transported conveniently to a suitable hydrogenation facility where H_2_ produced from renewable sources can be used to hydrogenate the spent fuel back to the LOHC (charged fuel carrier). Reviews on various perspectives of LOHCs, e.g., chemical and economic properties or supply chain strategies, have been reported earlier.^176−182^ Here, we focus on the recent developments in LOHCs facilitated by homogeneous transition metal catalysts.

#### 2.4.1 LOHCs Based on Carbocycles

Carbocycles contain several interesting properties such as high gravimetric storage capacities, being liquid at room temperature, high boiling points, low toxicity, CO-free dehydrogenation products, and high abundances, making them suitable candidates for LOHCs. The major drawback associated with carbocycles is the high barrier for the dehydrogenation reaction, requiring harsh reaction conditions for the dehydrogenation process such as a high temperature, 200–350 °C. This makes it challenging for using homogeneous catalysts for the dehydrogenation process, and most of the demonstrations of carbocycles as LOHCs involve heterogeneous catalysts. Early studies using heterogeneous catalysts were focused on LOHCs based on methylcyclohexane/toluene (6.1 wt % theoretical hydrogen storage capacity),^183,184^ cyclohexane/benzene (7.14 wt %),^185^ decalin/naphthalene (7.3 wt %),^186^ and perhydro-dibenzyltoluene/dibenzyltoluene (6.2 wt %).^187^ Remarkably, LOHC technologies based on methylcyclohexane/toluene and perhydro-dibenzyltoluene/dibenzyltoluene were recently commercialized by Chiyoda Corp.^188^ and Hydrogenious LOHC Technologies,^189^ respectively. However, issues of selectivity during (de)hydrogenation reactions due to the possibility of the formation of multiple intermediates remain to be sorted out, offering scope for the discovery of new and more efficient LOHCs.

#### 2.4.2 LOHCs Based on Heterocycles

**Scheme 16:**
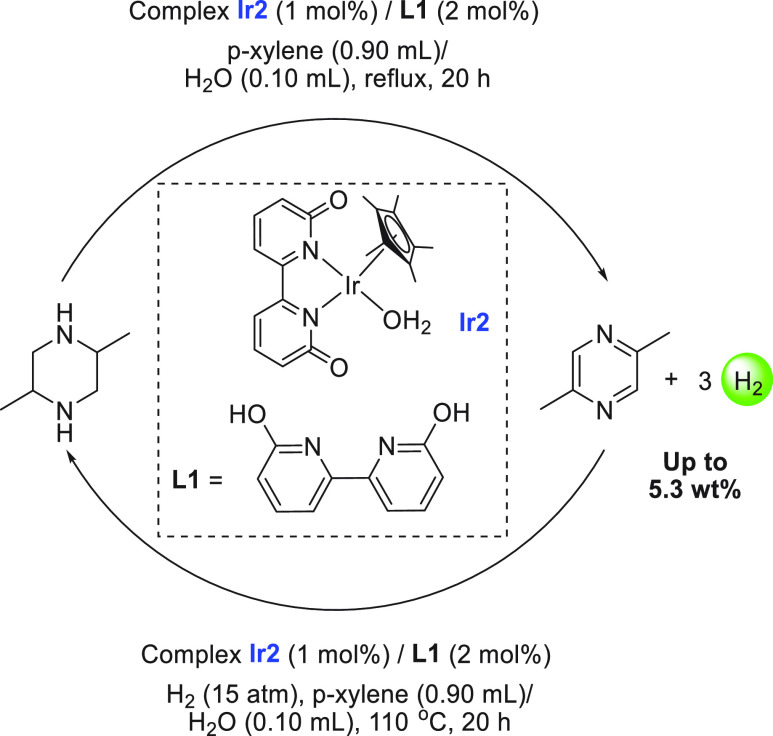
LOHC Based on 2,5-Dimethylpiperazine and 2,5-Dimethylpyrazine Using an Iridium Catalyst

Nitrogen- or oxygen-containing heterocycles are thermodynamically advantageous for dehydrogenation reactions compared to the corresponding carbocyclic compounds.^177,190,191^ For practical advantages heterogeneous catalysts have been investigated extensively to develop LOHCs based on heterocycles. A seminal discovery in this direction was made by Pez and co-workers, who developed an LOHC based on *N*-ethylcarbazole (NEC) with a hydrogen storage capacity of 5.8 wt %.^192^ Although a facile dehydrogenation and hydrogenation process has been demonstrated, the NEC/H_12_–NEC system has some drawbacks that limit its commercialization such as the solid state of *N*-ethylcarbazole and the formation of unwanted side products during dehydrogenation. Jessop and co-workers^193^ and Ke and Cheng^194^ independently reported an LOHC system based on octahydro-1-methylindole/1-methylindole constituting a hydrogen storage capacity of 5.8 wt %. In a similar direction, LOHC based on 2-[(*N*-methylcyclohexyl)methyl]piperidine/2-(*N*-methylbenzyl)pyridine with a hydrogen storage capacity of 6.1 wt % was reported by Suh and Park.^195^ Kempe discovered an LOHC based on phenazine (PHZ) that can be synthesized from cyclohexane-1,2-diol obtained from hydrogenolysis of lignin.^196^ With the use of a bimetallic catalyst, Pd_2_Ru@SiCN, PHZ was hydrogenated to form 14H-phenazine, making it a hydrogen storage material of 7.2 wt %. Milstein recently reported a solvent-free LOHC based on 2-picoline/2-methylpiperidine (6.1 wt %) and 2,6-lutidine/2,6-dimethylpiperidine (5.3 wt %) using a palladium-based heterogeneous catalyst generated *in situ*, efficient for both hydrogenation and dehydrogenation reactions under relatively mild conditions.^197^ In addition to the demonstration of LOHCs by heterogeneous catalysts, there are a couple of examples in which molecular complexes were employed. Fujita and co-workers reported the use of an iridium complex for the dehydrogenation of 2,5-dimethylpiperazine to 2,5-dimethylpyrazine releasing 3 equiv of H_2_ in the presence of a very small amount (0.5–1 mL) of solvent (Scheme 16).^198^ Under the catalytic conditions of **Ir2** (Scheme 16, 0.25 mol %) and 6,6′-dihydroxy-2,2′-bipyridine (**L1**, 0.50 mol %) in the *p*-xylene/water solvent, quantitative dehydrogenation of dimethylpiperazine to 2,5-dimethylpyrazine was observed. Moreover, hydrogenation of 2,5-dimethylpyrazine to 2,5-dimethylpiperazine took place quantitatively (Scheme 16). Furthermore, closed-loop conversion between 2,5-dimethylpyrazine and 2,5-dimethylpiperazine was also demonstrated with the quantitative yields at least four times.

#### 2.4.3 LOHCs Based on Alcohols and Amines

**Scheme 17:**
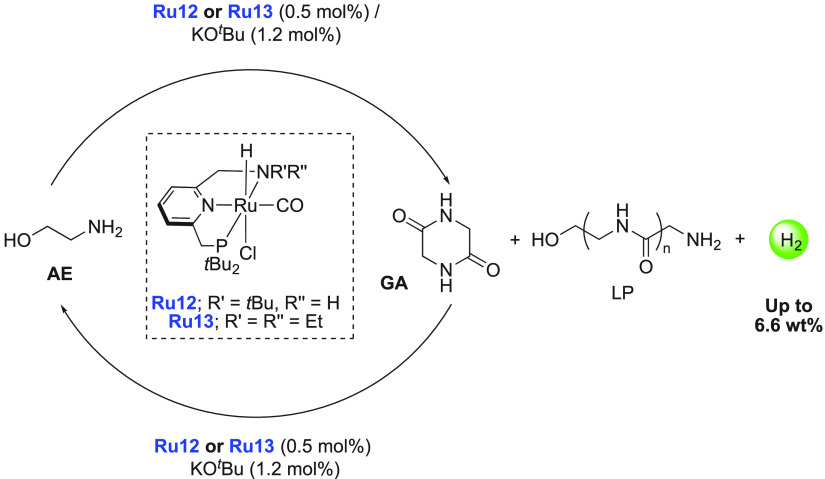
LOHC Based on 2-Aminoethanol (AE) and Glycine Anhydride (GA)/ Linear Peptides (LP)

A new approach in the area of LOHCs was revealed by Milstein and co-workers by utilizing the concept of acceptorless dehydrogenative coupling of amines and alcohols to form amides^199,200^ with the liberation of H_2_ and its reverse reaction—hydrogenation of amides to form alcohols and amines.^200^ In 2015, Milstein reported the first LOHC based on this concept involving the dehydrogenative self-coupling of the widely used, industrial, 2-aminoethanol (AE) and the reverse hydrogenation reaction exhibiting a theoretical hydrogen storage capacity of 6.56 wt % (Scheme 17).^201^ Using a combination of the ruthenium pincer complex **Ru12** (0.5 mol %) and KO^*t*^Bu (1.2 mol %), AE (85% conversion) was dehydrogenated to form the cyclic glycine anhydride (GA, 60%) as the major product and linear peptides (LP) as the side product. H_2_ gas was generated in 77% yield. The same catalyst system catalyzed the hydrogenation of glycine anhydride under 50 bar H_2_ at 110 °C, 48 h, in approximately quantitative yield. Importantly, the mixed products GA + LP produced from the dehydrogenation reaction were also successfully hydrogenated under the same catalytic conditions, indicating that the formation of linear peptides is not a problem for the regeneration of AE. Complex **Ru13** also catalyzed the same dehydrogenation and hydrogenation reactions although with lower yield and selectivity.

**Scheme 18:**
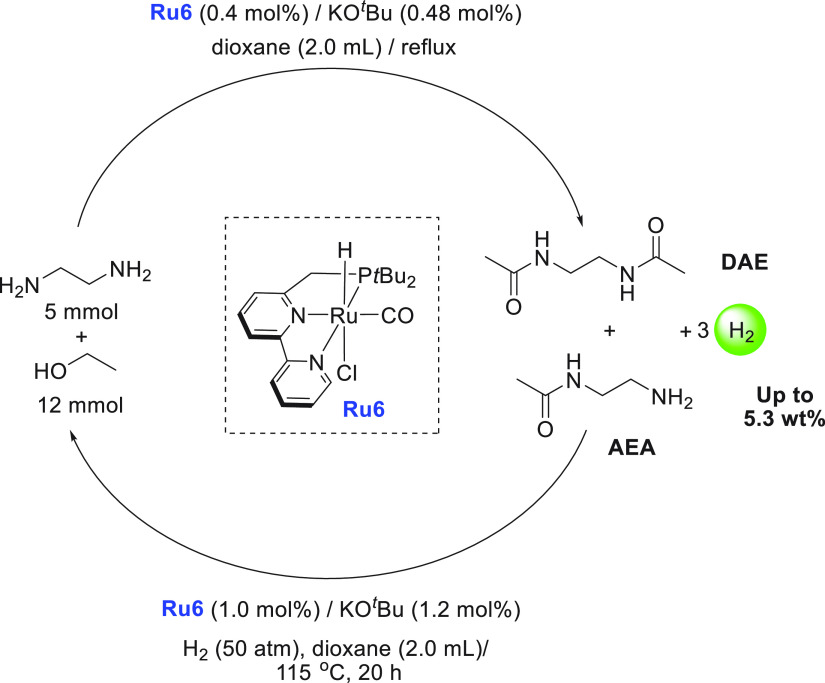
LOHC Based on Ethylenediamine and Ethanol Using a Ruthenium Pincer Catalyst

Furthermore, Milstein reported in 2016 an LOHC based on the dehydrogenative coupling of ethylenediamine and ethanol to form *N*,*N*′-diacetylethylenediamine (DAE) using the ruthenium pincer catalyst **Ru6** (Scheme 18).^202^ The same complex was also found to catalyze the reverse reaction, i.e., the hydrogenation of DAE to a mixture of ethylenediamine and ethanol, forming an LOHC system with a theoretical hydrogen storage capacity of 5.3 wt %. Under the catalytic conditions of complex **Ru6** (0.02 mmol) and KO^*t*^Bu (0.024 mmol), 5 mmol of ethylenediamine and 12 mmol of ethanol were dehydrogenated (100% conversion) in refluxing dioxane (2 mL) to form DAE in 93% yield and *N*-(2-aminoethyl)-acetamide (AEA) in 7% yield. Similarly, in the presence of complex **Ru6** (1 mol %) and KO^*t*^Bu (1.2 mol %), DAE was hydrogenated (50 bar H_2_, 115 °C) to form a mixture of ethylenediamine (91%), AEA (9%), and ethanol (70% yield). Repetitive demonstration of dehydrogenation/hydrogenation cycles was also demonstrated without adding more catalyst.

**Scheme 19:**
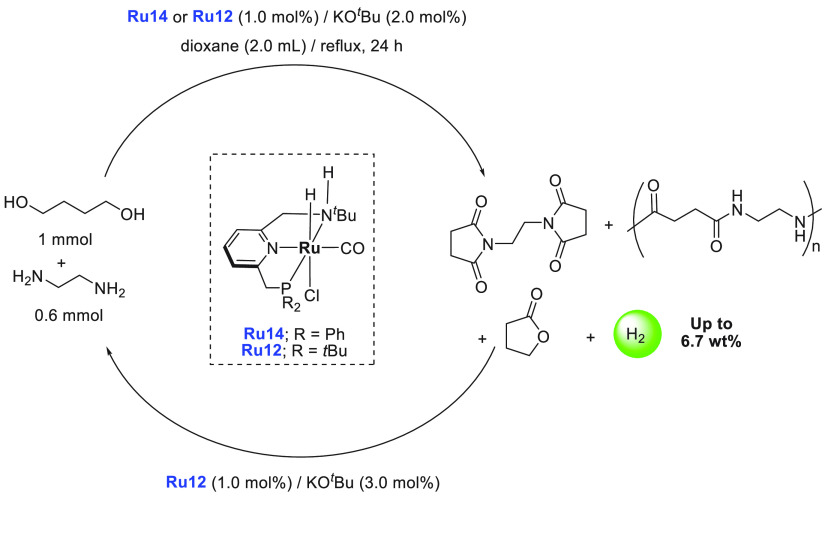
LOHC Based on Ethylenediamine and 1,4-Butanediol Using a Ruthenium Pincer Catalyst

Another LOHC based on alcohol and amine, with a theoretical hydrogen storage capacity of 6.66 wt %, was reported by Milstein in 2018 using 1,4-butanediol and ethylenediamine (Scheme 19).^203^ Employing the ruthenium pincer complex RuP^Ph_2_^NNH (**Ru14**, 1 mol %) in the presence of 2 mol % KO^*t*^Bu (2 mL 1,4-dioxane, 120 °C), catalytic dehydrogenation of 1,4-butanediol (1 mmol) and ethylenediamine (0.6 mmol) resulted in a mixture of bis-cyclic imide (70%), lactone (12%), and oligoamides. Pure H_2_ gas (82 mL), as confirmed by GC, was also collected. An analogous complex RuP^^*t*^Bu_2_^NNH (**Ru12**, 1 mol %) in the presence of KO^*t*^Bu (3 mol %) catalyzed the hydrogenation (40 bar H_2_, 135 °C, 40 h) of the bis-cyclic imide to regenerate 1,4-butanediol and ethylenediamine in approximately 90% yields.

**Scheme 20:**
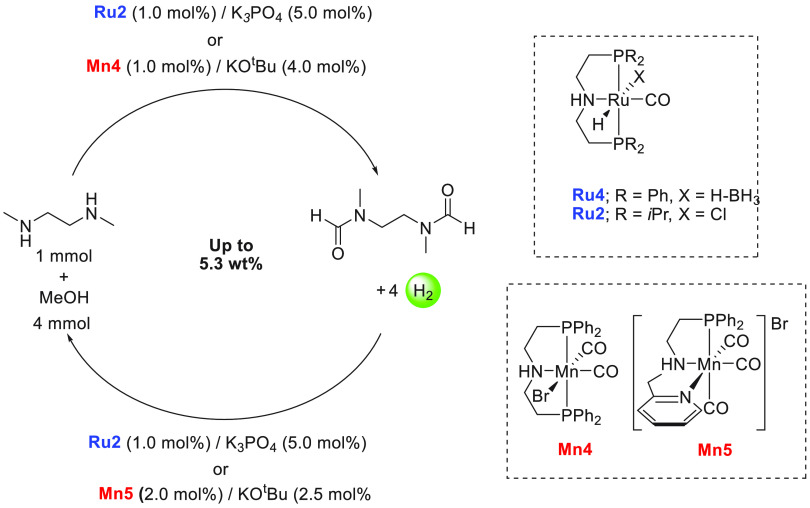
LOHC Based on *N*,*N*′-Dimethylethylenediamine/CH_3_OH and Diamide Using Ruthenium or Manganese Catalysts

Along a similar direction, Prakash reported an LOHC based on “amine reforming of methanol” in analogy with the steam reforming of CH_3_OH using a combination of *N*,*N*′-dimethylethylenediamine (DMEDA) and CH_3_OH that can exhibit a hydrogen storage capacity of up to 5.3 wt % (Scheme 20).^204^ With employing the ruthenium complex **Ru4** (1 mol %) and K_3_PO_4_ (4 mol %), the coupling of DMEDA (1 mmol) and methanol (4 mmol) at 120 °C for 24 h in toluene resulted in the formation of the corresponding diamide (75%) and monoamide (22%) along with hydrogen gas (86%). The undesired CO gas was detected by GC in 2.8% yield, suggested to originate from decarbonylation of intermediate formaldehyde, in competition with amine attack to form the α-amino alcohol intermediate. Interestingly, using the RuPNP^^*i*^Pr^ complex **Ru2** (1 mol %) and K_3_PO_4_ (5 mol %), keeping the remaining conditions the same, 90% yield of hydrogen gas was observed, and no CO was detected in the gas mixture. In order to study the reversibility of the reaction, the crude reaction mixture after dehydrogenation using catalyst **Ru2** was subjected to hydrogenation conditions (60 bar H_2_, 120 °C, 24 h) without adding any additional catalyst. After 24 h, the formation of DMEDA was observed in 95% yield. Furthermore, both dehydrogenation and hydrogenation reactions were performed under neat conditions without adding any solvent and gave good to moderate yields (76 and 60%, respectively). Recycling of the catalyst up to three times was also demonstrated.

In the direction of using earth-abundant metals in catalysis, Liu and co-workers have recently demonstrated the proof of concept of the same LOHC (DMEDA/CH_3_OH and diamide) using manganese-based catalysts (Scheme 20).^205^ The best dehydrogenation activity was achieved using the complex **Mn4**, whereas the complex **Mn5** exhibited the highest yield for the hydrogenation reaction. Compared to the ruthenium catalyst **Ru2**, a higher loading (2 mol %) and higher temperature (165 °C for 16 h) were used in the case of **Mn4**. Furthermore, a higher amount (∼5%) of CO gas was detected in the case of **Mn4** which could be suppressed by the addition of **Mn4** in two equal portions.

**Scheme 21:**
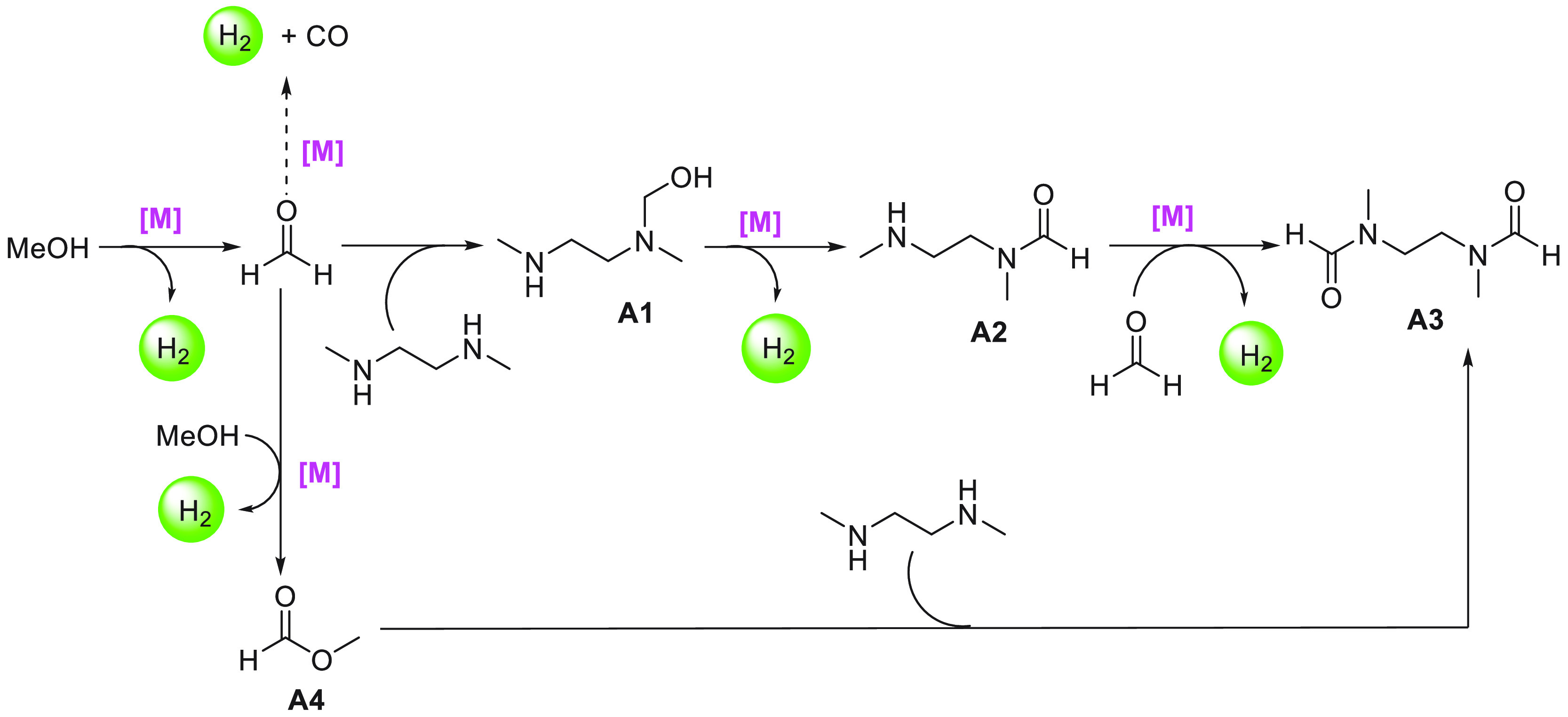
Proposed Mechanism for the Dehydrogenative Coupling of DMEDA and Methanol [M] = Ru or Mn catalysts.

Based on control experiments and earlier reports, a plausible mechanism was proposed for the manganese- or ruthenium-catalyzed dehydrogenative coupling of diamine and methanol (Scheme 21). Methanol first is dehydrogenated to formaldehyde that reacts with an amine moiety of DMEDA to generate the hemiaminal species **A1** that eliminates one molecule of H_2_ to afford the monoamide intermediate **A2**. **A2** reacts with another molecule of formaldehyde to produce the final diamide product **A3**. Another possible pathway could be via the formation of methyl formate (**A4**) from methanol that undergoes aminolysis with DMEDA to form the diamide **A3**. Fast condensation of formaldehyde with the amino groups and monoamide **A2** circumvents the decarbonylation of formaldehyde to CO and H_2_.

**Scheme 22:**
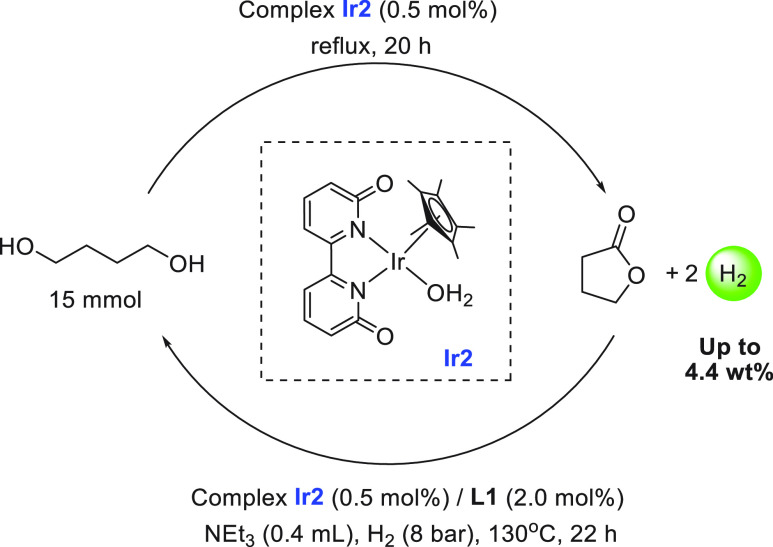
LOHC Based on 1,4-Butanediol and γ-Butyrolactone **L1** = 6,6′-dihydroxy-2,2′-bipyridine.

Besides using a combination of alcohols and amines, simple alcohols have also been demonstrated as LOHCs. Recently, Fujita has reported a reversible hydrogen storage system based on 1,4-butanediol/γ-butyrolactone^206^ catalyzed by the iridium complex **Ir2** reported earlier for the development of the LOHC based on 2,5-dimethylpiperazine/2,5-dimethylpyrazine (Scheme 16).^198^ Under the catalytic condition of 0.1 mol % **Ir2**, a solution of 1,4-butanediol in 1,2-dimethoxyethane under reflux for 20 h resulted in dehydrogenation to produce γ-butyrolactone and H_2_ gas in quantitative yields (Scheme 22). Solvent screening showed that anisole was a better solvent in which a complete dehydrogenation was obtained in only 3 h. Interestingly, the reaction also proceeded under neat conditions, and almost complete conversion of 1,4-butanediol (15 mmol) to γ-butyrolactone was obtained in the presence of 0.5 mol % catalyst **Ir2** and hydrogen gas was collected in 99% yield. Moreover, the reverse reaction, i.e., hydrogenation of γ-butyrolactone to 1,4-butanediol, was also demonstrated under neat conditions using **Ir2** and ligands such as 6,6′-dihydroxy-2,2′-bipyridine (**L1**) and triethylamine. Furthermore, successive interconversions between γ-butyrolactone and 1,4-butanediol were also demonstrated under neat conditions, and both the dehydrogenation and hydrogenation steps were accomplished with almost quantitative yields. However, the theoretical hydrogen storage capacity of this system is lower than those of the systems reported using alcohols/amines and is limited to 4.4 wt %.

**Scheme 23:**
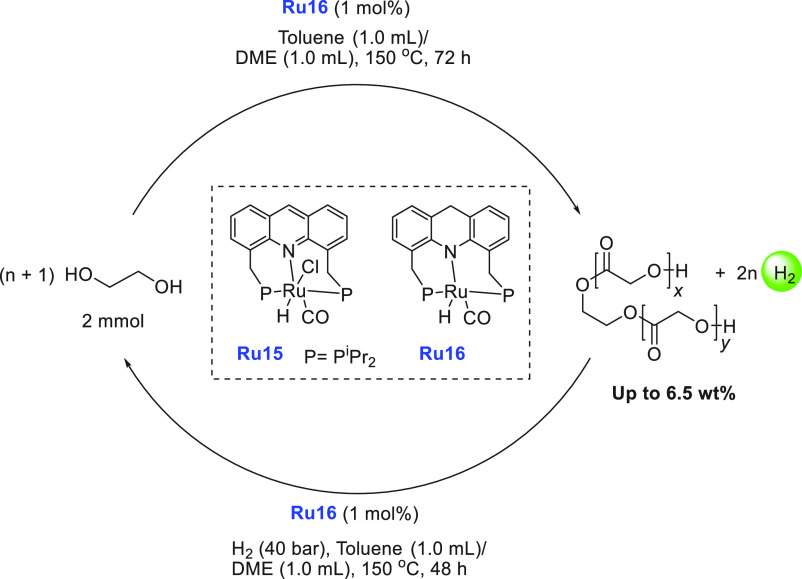
LOHC Based on EG and Oligoester Using a Ruthenium Catalyst

In a recent advance, Milstein and co-workers reported an LOHC system based on ethylene glycol (EG).^207^ EG is industrially produced on a large scale with a global annual production of 34 million tons, and it has applications in the automobile and polyester industries.^208^ Additionally, EG can be produced from biomass-derived hydrocarbons, making it a sustainable and renewable LOHC.^209^ However, dehydrogenation of ethylene glycol presents additional challenges such as catalyst poisoning by chelation of the vicinal 1,2-diol; the formation of α-keto ester byproduct, which can readily decompose to aldehyde and CO which could again poison the catalyst; and formation of unwanted cyclic side products such as 1,3-dioxolan-2-ylmethanol, resulting in lower hydrogen capacities. However, remarkably, with the use of the acridine-based ruthenium PNP complex **Ru15**,^210,211^ these difficulties were circumvented to some extent. In the presence of **Ru15** (1 mol %) and KO^*t*^Bu (1 mol %), ethylene glycol (2.0 mmol) in toluene/DME (1.0 mL/1.0 mL) at 150 °C and 72 h was dehydrogenated to produce 54 mL of hydrogen gas, 2-hydroxyethyl glycolate (HEG) (33%), and oligoesters (EG conversion 94%) (Scheme 23). Interestingly, dehydrogenation of EG proceeded smoothly in the presence of the dearomatized ruthenium PNP complex **Ru16** (1 mol %) under base-free conditions (keeping the remaining conditions the same), producing 61 mL of hydrogen gas and oligoesters of up to hexamer, with the conversion of EG being 97%. Interestingly, the reaction mixture obtained from the dehydrogenation process was hydrogenated back to ethylene glycol in excellent yield (92%) by using **Ru16** (1 mol %) and 40 bar H_2_ (48 h) in toluene/DME solvent, demonstrating the utility of EG as an LOHC. Moreover, the reaction was also demonstrated under *solvent-free* conditions at a reduced pressure (95 mbar, EG, 35.9 mmol, 2 mL) at 150 °C that resulted in 94% conversion of EG in 7 days.

**Scheme 24:**
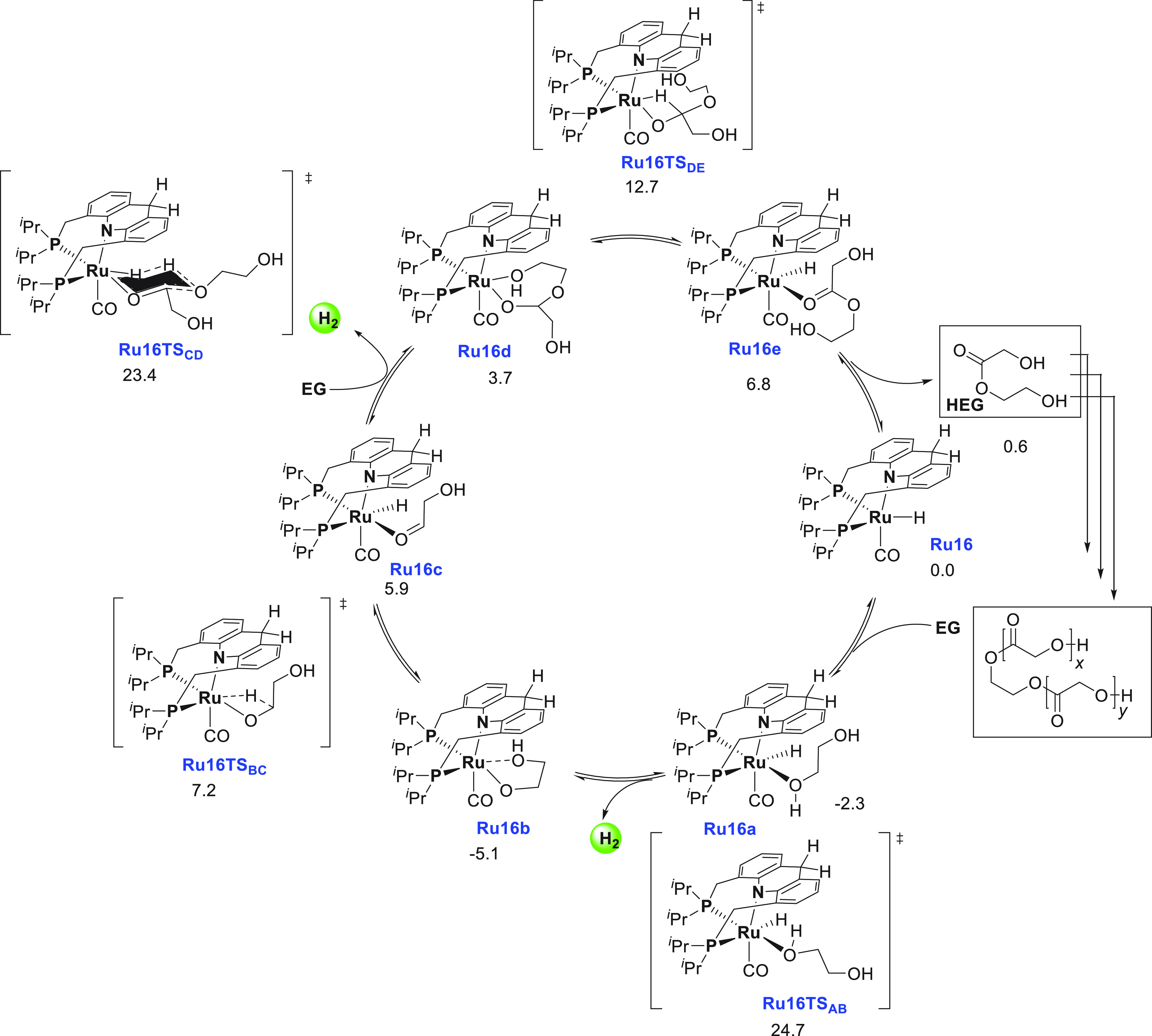
Proposed Catalytic Cycle for Dehydrogenative Coupling of EG to Form Polyester Using a Ruthenium Catalyst All values correspond to Gibbs free energies at 423.15 K (in kcal·mol^–1^ with respect to the starting material). Reproduced with permission from ref (207). Copyright 2019 Springer Nature.

A catalytic cycle was proposed based on the DFT calculations (Scheme 24). As outlined in Scheme 24, the first step is the coordination of EG to complex **Ru16** to form intermediate **Ru16a**, followed by the dehydrogenation step via **Ru16TS**_**AB**_ (24.7 kcal·mol^–1^) resulting in the formation of a κ^2^-alkoxide coordinated **Ru16b** (−5.1 kcal·mol^–1^). Hemilability of the hydroxy group facilitates the β-hydride elimination step via **Ru16TS**_**BC**_ (7.2 kcal·mol^–1^) forming a coordinated hydroxyaldehyde intermediate **Ru16c** (5.9 kcal·mol^–1^). Attack of another molecule of EG on the coordinated aldehyde followed by dehydrogenation via a concerted Zimmerman–Traxler-type six-membered transition state (**Ru16TS**_**CD**_, 23.4 kcal·mol^–1^) results in the formation of intermediate **Ru16d** (3.7 kcal·mol^–1^). A similar decoordination of the hydroxy group and β-hydride elimination step results in the formation of metal-bound ester **Ru16e** (6.8 kcal·mol^–1^) which can regenerate complex **Ru16** by dissociation of 2-hydroxyethyl glycolate (HEG). Subsequently, HEG can generate an oligoester through similar cycles.

#### 2.4.4 LOHCs Based on Formic Acid and CO_2_

Formic acid (FA) has attracted significant interest as a potential hydrogen storage material due to several qualities. For example, FA is a kinetically stable liquid at room temperature and is produced on a large scale by the chemical industry as well as by biomass fermentation. Although the theoretical hydrogen storage capacity (4.4 wt %) is lower than the target set by the U.S. Department of Energy for 2020 (5.5 wt %), the dehydrogenation process to produce CO_2_ is thermodynamically favorable (Δ*G* = −32.9 kJ/mol) at room temperature. Several examples have been reported for the catalytic dehydrogenation of HCOOH, and this topic has also been reviewed multiple times in the past.^114,212−221^ Laurenczy and Beller, in 2018, published a detailed review on the dehydrogenation of formic acid catalyzed by both noble metals and non-noble metals.^114^ Zell and Langer, in 2019, reviewed the dehydrogenation of formic acid catalyzed by both homogeneous and heterogeneous catalysts.^218^ Huang published an update on the dehydrogenation of formic acid using homogeneous catalysts in 2020.^220^ Two review articles, one by Yao and co-workers^222^ and another by Himeda,^223^ have been published in 2021 on the catalytic dehydrogenation of formic acid. The reverse reaction—direct hydrogenation of CO_2_ to HCOOH—has also been well-reviewed in the past.^224,225^

Different from dehydrogenation, hydrogenation of CO_2_ to HCOOH is thermodynamically uphill. Three approaches have been used to overcome the thermodynamic barrier for the hydrogenation of CO_2_ to HCOOH: (a) use of a stoichiometric base to stabilize HCOOH as a formate salt, (b) use of a polar solvent such as DMSO to stabilize HCOOH by H-bonding,^226,227^ and (c) use of ionic liquids containing basic anions such as 1,3-propyl-2-methylimidazolium formate to improve the solubility of CO_2_ and stabilize the product HCOOH.^228^ However, it is important to note that for a reversible process, as required for an LOHC, the additive used or the byproduct formed during hydrogenation should be compatible with the dehydrogenation reaction or *vice versa* in order to avoid purification and generation of waste. For example, dehydrogenation of formic acid can be carried out in an acidic medium, but CO_2_ hydrogenation might require a basic medium. Thus, the development of compatible catalysts and the choice of an appropriate hydrogen storage couple becomes highly important. Here we present a synopsis of the reports demonstrating HCOOH as a reversible hydrogen storage system catalyzed by a transition metal complex.

As the regeneration of HCOOH from CO_2_ poses a thermodynamic challenge, Papp and Joó reported a reversible hydrogen storage system based on aqueous HCOONa that was dehydrogenated to NaHCO_3_ in the presence of a water-soluble catalyst [RuCl(mtppms)_2_]_2_ (mtppms = sodium diphenylphosphinobenzene-3-sulfonate), originally developed by Laurenczy and co-workers.^229^ No additive or tuning of pH was required for this reversible process. Hydrogenation reaction was achieved at 100 bar H_2_, at 80 °C, and dehydrogenation or decomposition reaction was performed in a glass pressure tube at 80 °C. Almost no CO gas (≤10 ppm) was detected in the gas phase by GC.

**Scheme 25:**
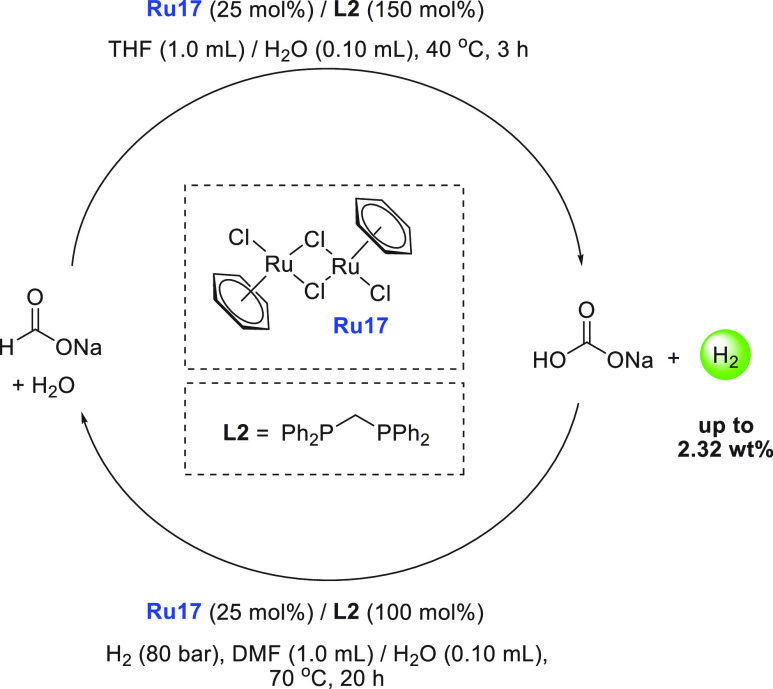
Hydrogen Storage System Based on HCOONa, H_2_O, and NaHCO_3_ Using a Ruthenium Catalyst

In the same direction, Beller and co-workers reported a reversible hydrogen storage system based on dehydrogenation of aqueous formate solution to a bicarbonate salt using a ruthenium/phosphine catalyst and the reverse reaction, i.e., hydrogenation of bicarbonates to formates.^230^ A catalytic combination of [RuCl_2_(benzene)]_2_/dppm (1:4 ratio) (dppm = diphenylphosphinomethane) was used for the hydrogenation of several alkali metal bicarbonates out of which NaHCO_3_ gave the best result where HCOONa was formed in 96% yield (TON = 1108) in 20 h at 70 °C under 80 bar H_2_ pressure (Scheme 25). The addition of external CO_2_ pressure enhanced catalytic activity. Moreover, dehydrogenation of various formates was explored using Ru/dppm at 60 °C in a DMF/H_2_O solution. Production of hydrogen gas was observed in all cases without detection of any CO gas. The use of excess water assisted in the liberation of H_2_ from formates that shifts the pH of the solution to a more basic medium. The basic solution could trap the generated CO_2_ and allow it to precipitate as bicarbonate during the reaction. Under the catalytic conditions of 5.0 mmol of [{RuCl_2_(benzene)}_2_] (**Ru17**) and 30 mmol of dppm (Ru/P = 1:6), 20 mmol of HCOONa was dehydrogenated at 40 °C to produce 490 mL of hydrogen gas with a TON (3 h) of 2000. A limitation of the above-described process reported by Beller is the use of different solvent systems for dehydrogenation (THF/H_2_O) and hydrogenation (DMF/H_2_O) reactions, which poses a practical challenge in the utilization of this system for reversible hydrogen storage. Moreover, the theoretical hydrogen storage capacity of the HCOONa and H_2_O/NaHCO_3_ system is only 2.32 wt %.

**Figure 2:**
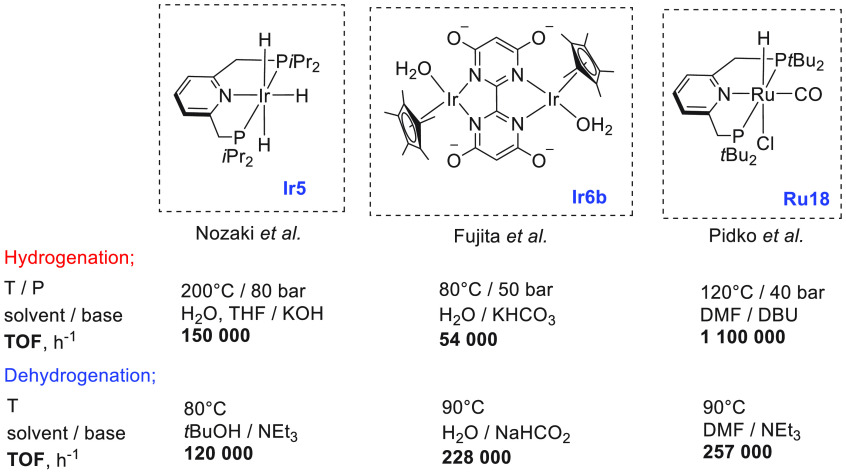
Catalytic conditions for the reversible conversion of CO_2_ to HCOOH.

In 2009, Nozaki reported the hydrogenation of CO_2_ to formate in a basic medium catalyzed by the iridium trihydride pincer complex [Ir(H)_3_(P^^*i*^Pr^N^Py^P^^*i*^Pr^)] (**Ir5**, Figure 2).^231^ The catalytic activity of this complex was the highest of its time (TOF 150 000 h^–1^ at 200 °C, 50 bar, and TON 3 500 000 at 120 °C). The same complex was also later utilized for the dehydrogenation of formic acid, albeit with a relatively lower catalytic activity (TON_43 h_ 890 at 60 °C) compared to that of the hydrogenation of CO_2_.^232^ Addition of base significantly enhanced the catalytic activity, e.g., TON_5 h_ of 4900 at 60 °C in the case of triethanolamine as a base. Although the reversibility of the HCOOH/CO_2_ + H_2_ system was studied, the demonstration of a charging/discharging cycle for the purpose of using formic acid as an LOHC was not performed in this study. Following this discovery, several homogeneous catalysts were reported for the demonstration of HCOOH/CO_2_ as a reversible hydrogen storage system.

In 2012, Laurenczy and Beller reported a reversible conversion of CO_2_ to formic acid (FA) catalyzed by a ruthenium complex [RuCl_2_(benzene)]_2_ (**Ru17**) and bisphosphine (diphenylphosphinoethane (dppe) or diphenylphosphinomethane (dppm)) ligand.^233^ Hydrogenation of CO_2_ to formic acid was accomplished using [RuCl_2_(benzene)]_2_ (10 mmol) and dppm (6 equiv) under a basic solution (NEt_3_) and a moderate pressure (30 bar H_2_ + 30 bar CO_2_). A reaction temperature of 100 °C was required for the generation of the active species, after which the catalysis could proceed at room temperature. This strategy was also applied to demonstrate hydrogen loading and unloading to establish a “hydrogen battery”.

**Scheme 26:**
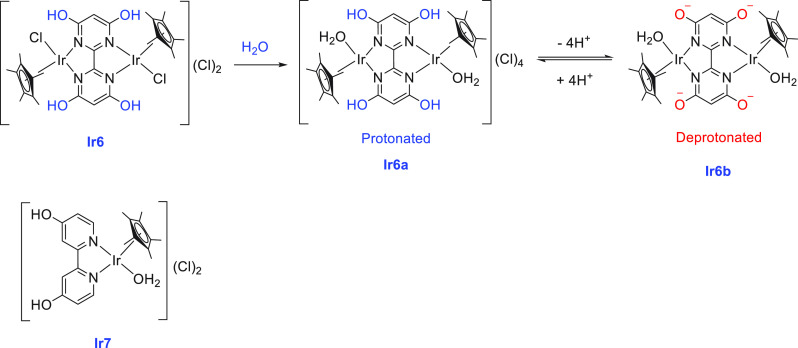
Relationship among Complexes **Ir6**, **Ir6a**, and **Ir6b**, and the Structure of Complex **Ir7**

Soon after, Hull, Himeda, and Fujita reported a reversible hydrogen storage system catalyzed by an iridium complex using CO_2_, formate, and formic acid under mild conditions.^80^ Drawing inspiration from hydrogen bonding in nature and the use of bases to relay protons in enzymatic catalysis, the authors developed new iridium complexes based on proton-responsive ligands bearing pendent-base moieties that could assist the interaction of H_2_, CO_2_, H_2_O, HCO_2_H, HCO_2_^–^, and H^+^ in the primary coordination sphere of iridium. Complex **Ir6**, synthesized using the bridging ligand 4,4′,6,6′-tetrahydroxy-2,2′-bipyrimidine, rapidly hydrolyzes in water to produce **Ir6a** (Scheme 26). Increasing the pH to more than 5 results in deprotonation of the ligand phenol moieties and the formation of **Ir6b**. Both catalysts **Ir6a** and **Ir6b** were active for storing and releasing H_2_ under mild conditions. In the presence of **Ir6b** as the catalyst, a 1:1 H_2_/CO_2_ mixture (0.1 MPa) was converted to 0.36 M formate (TON 7200) at 25 °C (pH 8.4) with an initial TOF of 64 h^–1^. This activity was found to be superior to those of **Ir7** (initial TOF 7 h^–1^) and [{Ir(Cp*)(OH_2_)}_2_(bpym)]Cl_2_ (initial TOF 0 h^–1^), suggesting that the pendent hydroxy base plays an important role in the hydrogenation process. The reverse reaction, i.e., the dehydrogenation of formic acid to CO_2_, was studied under acidic conditions. Under pH 3.5, complex **Ir6b** is partially protonated and exists in an intermediary form between **Ir6b** and **Ir6a**. Dehydrogenation of an aqueous solution of HCOOH/HCOONa at pH 3.5 by partially protonated **Ir6b** produces a 1:1 mixture of H_2_/CO_2_ with a remarkably high TOF (228 000 h^–1^ at 90 °C and TON of 308 000 at 80 °C).

**Scheme 27:**
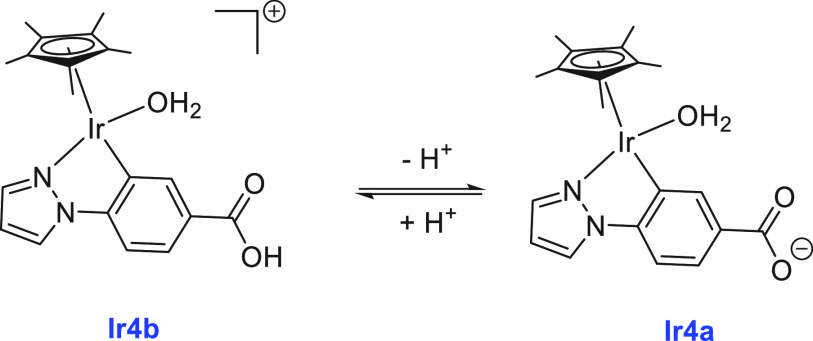
pH-Dependent Relationship of Complexes **Ir4a** and **Ir4b**

Along the direction of pH-dependent charging and discharging of formic acid, Fukuzumi, in 2013, reported the hydrogenation of CO_2_ using an iridium complex (**Ir4a**) in a weakly basic medium (pH 7.5 in H_2_O) at ambient temperature and pressure.^234^ The dehydrogenation of formic acid was demonstrated at pH 2.8 (acidic medium) by using **Ir4a** at room temperature (Scheme 27).

Pidko in 2014 reported the reversible hydrogenation of CO_2_ using the ruthenium PNP pincer catalyst **Ru18** (Figure 2).^235^ Optimization of catalytic conditions for the dehydrogenation of FA revealed that a catalytic combination of **Ru18** and NR_3_ (R = Et, hexyl) in DMF solvent is highly efficient. A catalytic combination of **Ru18** (1.42 μmol) and NEt_3_ (33.5 mmol) in DMF (35 mL total volume) exhibited significant dehydrogenation of FA with TOF of 257 000 h^–1^ at 90 °C (TON = 326 500). Catalytic hydrogenation of CO_2_ to formate under a basic medium was also demonstrated by using the complex **Ru18** in DMF. The combination of **Ru18** and 1,8-diazabicyclo[5.4.0]undec-7-ene (DBU) at 120 °C (H_2_, 30 bar, and CO_2_, 10 bar) resulted in a TOF of 1 100 000 h^–1^, which is the highest reported catalytic activity for the hydrogenation of CO_2_ to date, higher than the earlier record value reported by Nozaki^231^ using the Ir pincer complex **Ir5** and that by Fujita using **Ir6b** (Figure 2).^80^ The nature of the base was found to significantly affect the catalytic outcome. For example, (a) a higher TON was obtained (TOF = 256 000 h^–1^ and TON = 706 500) in the case of a less volatile amine such as trihexylamine, in comparison to that of NEt_3_ for FA dehydrogenation; (b) a stronger base resulted in a higher AAR (acid to amine ratio) at higher temperature and shorter reaction time; and (c) the rate-determining step of the catalytic cycle was found to be influenced by the strength of the base (e.g., DBU and NEt_3_).

**Scheme 28:**
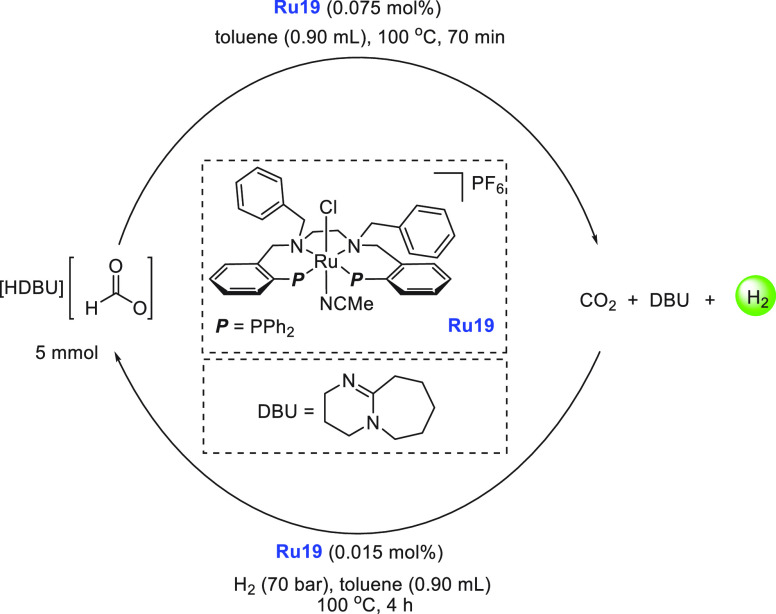
Hydrogenation of CO_2_ to a Formate Salt and the Reverse Reaction Using the Ruthenium Catalyst **Ru19**

In a similar direction, Plietker and co-workers in 2014 reported a reversible hydrogen storage system based on amine/CO_2_ using a Ru-PNNP catalyst (**Ru19**) (Scheme 28).^236^ The charged system could be stored for several days without loss of efficiency, and several hydrogenation and dehydrogenation cycles were demonstrated without changing the catalyst or the reaction vessel. Efficient hydrogenation of CO_2_ (dry ice) was accomplished by using catalyst **Ru19**. Using 0.015 mol % **Ru19** and 65.7 mmol of DBU, at 100 °C and 70 bar H_2_, 20 g of dry ice was hydrogenated to the corresponding DBU formate salt in 4 h (yield 84% and TON = 5600). Interestingly, at a higher catalyst loading (0.075 mol % **Ru19**), a bis-formic acid DBU adduct, instead of a monoadduct, was formed, allowing higher hydrogen storage capacity of this system. The same catalyst also catalyzed the dehydrogenation reaction. In the presence of 0.075 mol % **Ru19**, complete dehydrogenation of a 1:1 adduct of HCOOH and DBU was observed at 100 °C in 70 min. Interestingly, the addition of toluene increased the dehydrogenation rate. CO was not detected in the gas phase down to 10 ppm. Under these catalytic conditions, up to five charge–discharge cycles were performed in the same reactor, without deterioration of the catalyst and high product yields (formate salts and H_2_ + CO_2_ mixture) were obtained at the end of each cycle.

**Scheme 29:**
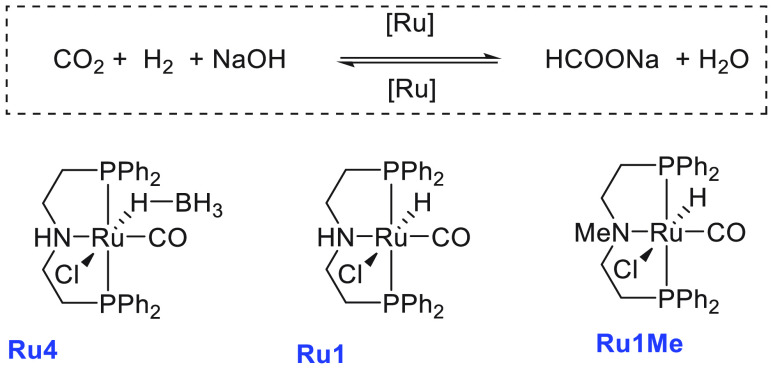
Ruthenium Pincer Complexes Used for the Reversible Hydrogenation of CO_2_ to Formic Acid under Basic Conditions

Along the same direction, an LOHC based on HCOONa/CO_2_ in the basic medium was reported by Czaun, Prakash, Olah, and co-workers.^237^ Both reactions, i.e., dehydrogenation of HCOONa and hydrogenation of CO_2_ (in a basic medium), were catalyzed by ruthenium pincer complexes under relatively mild conditions without any need for external pH control. Dehydrogenation of formate salts (20 mmol) was studied using pincer complexes **Ru4**, **Ru1**, or **Ru1Me** (20 mmol) in 1,4-dioxane/H_2_O solvent at 69–84 °C (Scheme 29). Both catalysts **Ru4** and **Ru1** exhibited similar catalytic activities (initial TOF = 286 h^–1^) for the dehydrogenation of HCOONa, and an almost quantitative conversion of HCOONa was observed in ∼300 min at 70 °C producing approximately 490 mL of hydrogen gas. Interestingly, complex **Ru1Me** was found to be more active in this reaction and quantitative dehydrogenation of HCOONa was observed in 4 h under the same conditions, giving an initial TOF of 430 h^–1^. This suggests that the N–H moiety does not influence the rate of catalysis, in contrast to several other reports using analogous systems where N–H plays an important role in (de)hydrogenation reactions. The reverse reaction, i.e., hydrogenation of CO_2_ to formate (H_2_/CO_2_ pressure 30/30 bar) in the presence of NaOH was also accomplished by using complex **Ru4** at 76 °C in THF/H_2_O solvent resulting in the formation of sodium formate in 93% yield (TON = 1160). Similar to dehydrogenation, complex **Ru1Me** was also active for the hydrogenation reaction, suggesting that the N–H moiety is not important in this transformation. Furthermore, hydrogenation of bicarbonates and carbonates was also accomplished under similar catalytic conditions.

Closed-loop cycles consisting of CO_2_ hydrogenation (charging) and formate dehydrogenation (discharging) were also demonstrated by using catalyst **Ru4** at 78 °C up to six hydrogenation–dehydrogenation cycles. Significantly, CO was not detected in the evolved gas mixture in any of the catalytic cycles. Furthermore, the reversibility of this hydrogen storage system was also demonstrated in the same pot.

**Scheme 30:**
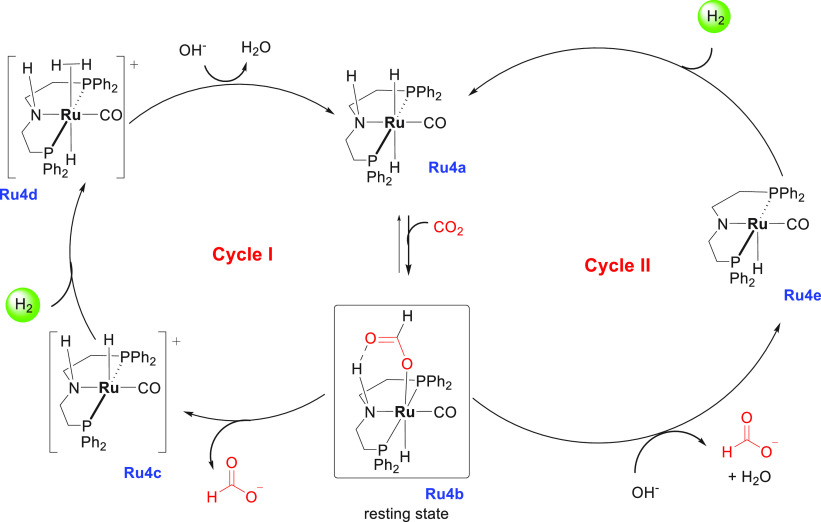
Proposed Mechanism of the Hydrogenation of CO_2_ to Formate Using a Ru Pincer Catalyst Reproduced with permission from ref (237). Copyright 2015 John Wiley and Sons.

On the basis of their mechanistic investigations, two possible pathways for the catalytic cycle were proposed (Scheme 30). In both pathways, the first step is the insertion of CO_2_ into the Ru–H bond of complex **Ru4a** to form a ruthenium formate complex, **Ru4b**. In the first pathway (cycle I), the formate dissociates to form the 16-electron complex **Ru4c** followed by its reaction with H_2_ to produce the dihydrogen complex **Ru 4d**. The abstraction of a proton from complex **Ru 4d** results in the regeneration of the *trans*-dihydride complex **Ru4a** and H_2_O. The second pathway (cycle II) involves the formation of an amido complex, **Ru4e**, by reaction of a base with **Ru4b** as proposed earlier for the transfer hydrogenation of ketones using Ir-H_3_[(^*i*^Pr_2_PCH_2_CH_2_)_2_NH].^238^ Addition of H_2_ to complex **Ru4e** regenerates the complex **Ru4a**. As cycle II involves deprotonation of the N–H proton, catalysis performed by the complex **Ru1Me** occurs most likely via cycle I (Scheme 30).

### 2.5 Hydrogen Production from Biomass and Water Splitting

Another challenge, in addition to storage, in front of a viable hydrogen economy is that of sustainable production of H_2_. As of 2018, ∼95% of hydrogen gas was produced from fossil fuels, mainly via steam reforming of natural gas,^239^ resulting in dependence on the depleting fossil fuels, and also producing large amounts of the greenhouse gas CO_2_. Relevant to this review are the opportunities that biomass presents as a potential renewable feedstock for H_2_ production.^240^ Several catalysts, mostly heterogeneous, have been reported for the production of hydrogen gas using gasification or thermochemical conversion of biomass.^240−242^ Recently, a few transition metal complexes have been utilized for the efficient production of H_2_ from biomass feedstock, e.g., (bio)ethanol,^243−245^ glycerol,^246−250^ and polyols, e.g., sugar alcohols.^251−254^ Detailed review articles featuring dehydrogenation of alcohols catalyzed by transition metal complexes have been reported recently.^255−257^

**Scheme 31:**
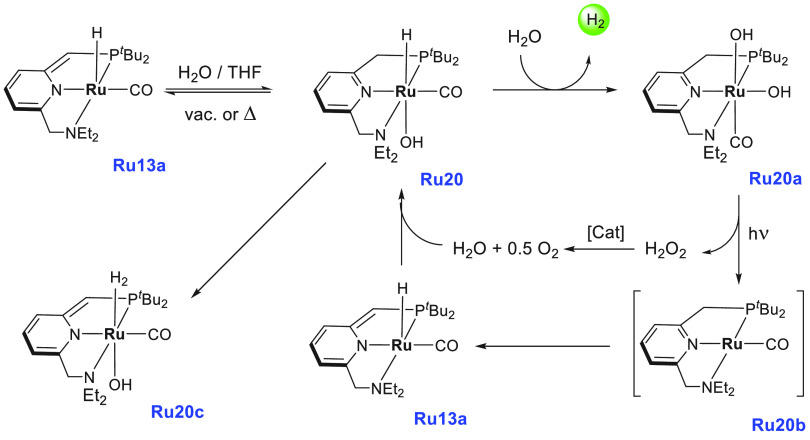
Consecutive Water Splitting Using a Ruthenium Pincer Complex with Suggested H_2_O_2_ Intermediacy

A very attractive strategy is water splitting, which offers a sustainable way to produce clean H_2_ and O_2_, which is the reverse reaction occurring in a hydrogen fuel cell.^258^ Currently, water splitting is mainly performed by electrolysis, but much research is directed at photoelectrochemical water splitting and photocatalytic water splitting, as extensively reviewed in recent years.^259−265^ An efficient and economic water-splitting technology can result in a breakthrough needed to underpin the hydrogen economy. In a fundamentally new approach, Milstein in 2009 reported a stoichiometric stepwise water-splitting reaction where a ruthenium pincer complex splits H_2_O first thermally, producing H_2_ gas, and then photolytically to produce O_2_ gas.^266^ The addition of 1 equiv of H_2_O to the Ru-PNN dearomatized complex **Ru13a** resulted in the reversible O–H activation of water via metal–ligand cooperation, forming the ruthenium hydride hydroxo complex **Ru20** (Scheme 31). Significantly, heating complex **Ru20** in refluxing water for 3 days resulted in the formation of the *cis* dihydroxo complex **Ru20a** with concomitant evolution of hydrogen gas. Moreover, irradiation of complex **Ru20a** (in THF or water) with a 300 W halogen lamp filtered through Perspex for 2 days resulted in the regeneration of the ruthenium hydrido-hydroxo complex **Ru20** with the concomitant formation of oxygen gas. It was suggested that complex **Ru20a** eliminated H_2_O_2_ via reductive elimination of two hydroxo ligands followed by decomposition of H_2_O_2_ to form H_2_O and O_2_. Labeling experiments were performed to confirm that O–O bond formation in this system is intramolecular and involves only a single metal center (Scheme 31).

**Scheme 32:**
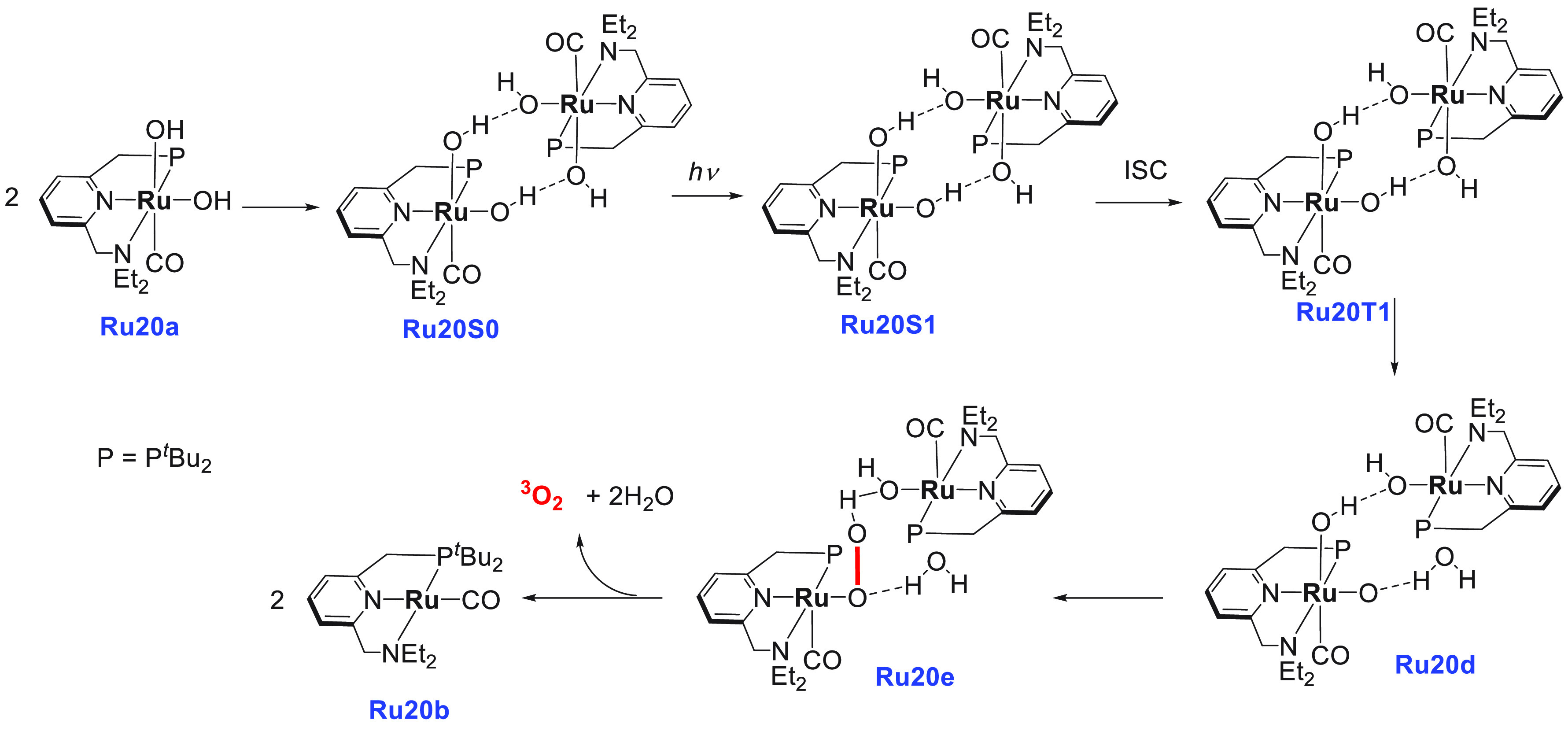
Proposed DFT Mechanism for the Elimination of O_2_ from **Ru20a** Complex

Soon after this, successive DFT calculations were performed by Yoshizawa^267^ and Hall.^268^ Both calculations suggested that the heterolytic coupling of ruthenium hydride with a proton from the side arm of the pincer ligand in **Ru20** forming the dearomatized sigma-complex **Ru20c** is the rate-limiting step. Further lower energy pathways have been proposed independently by Suresh^269^ and Fabris,^270^ where solvolysis by H_2_O was found to play an important role. Suresh performed a systematic study to explore how modification of Milstein’s RuPNN complex can affect the rate-determining H_2_ elimination step.^271^ The study revealed that decreasing the steric bulk of the phosphine moiety allows the exothermic association of a water molecule with the ruthenium center and makes the intermediates and transition states more stable. Thus, replacing the bulky *tert*-butyl group with a methyl or ethyl group at the phosphine moiety of the pincer complex can reduce the Δ*G*^⧧^ by a significant amount, such as ∼10 kcal/mol. As Ru–H facilitates H···H interaction with H_2_O for H_2_ elimination in the water-splitting reaction,^269^ Suresh studied the hydridic character of several transition metal hydride complexes using the approach of the molecular electrostatic potential (MESP).^272^ The study suggested that a lower barrier for the activation of H_2_ can be obtained if a more electron-rich hydride ligand is used. On the basis of DFT calculations, Fang^273^ proposed a new mechanism for the formation of triplet O_2_ that does not involve H_2_O_2_ intermediacy and involves the formation of a dimer, **Ru20S0** (Scheme 32). Photoexcitation of this dimer results in a change of a spin state and the formation of the **Ru20T1** complex via the intersystem crossing (ISC) of **Ru20S1.** This is followed by the first hydrogen atom transfer (HAT) and the concerted dehydration steps to form **Ru20d**. Here the second HAT and the dehydration steps occur which are accompanied by a concerted formation of the O–O bond in the complex **Ru20e**. The elimination of triplet O_2_ and H_2_O molecules from **Ru20e** results in the formation of **Ru20b**. This mechanism agrees with the experimental results from the Milstein group.

## 3 Fuels from Biomass

### 3.1 Advanced Biofuel from Ethanol

**Scheme 33:**
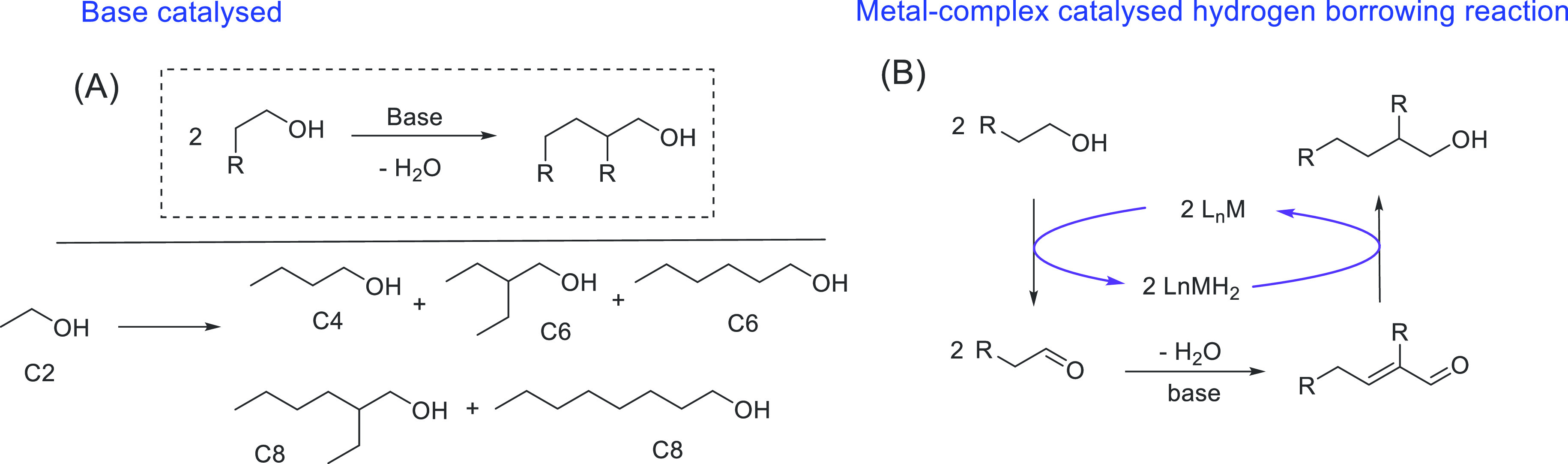
Guerbet Reaction via (A) Condensation and Higher Oligomer Formation and (B) Borrowing Hydrogenation Pathway Catalyzed by Transition Metal Complexes

Our current demand for transportation fuel is largely met by fossil fuels, causing environmental and sustainability concerns. Biofuels which can be produced from renewable biomass have been proposed as a sustainable alternative to fossil fuels.^274−278^ One of the examples of biofuels is bioethanol that can be produced via fermentation of biomass. However, direct use of ethanol as a fuel has some drawbacks such as its low energy density, corrosive nature, and formation of an azeotrope with water causing separation problems, which limits its application in the transportation sector. On the other hand, butanol isomers have a high energy density and noncorrosive nature and are immiscible with water, more closely resembling gasoline. Therefore, butanol isomers such as *n*-butanol are termed “advanced biofuels”.^279−281^ Thus, the synthesis of butanol from a bio-based feedstock such as ethanol is an attractive topic. An important approach in this direction is based on the “Guerbet reaction”,^282,283^ discovered by Guerbet more than 100 years ago using simple sodium alkoxides as catalysts at elevated (200 °C) temperature (Scheme 33A).^284,285^ Although simple and elegant, the process suffers from a selectivity issue as a mixture of oligomers and polymers is formed under the reaction conditions. As transition metal catalysts can allow the control of kinetics and the oligomerization process, the “Guerbet reaction” has been expanded to a “hydrogen borrowing reaction” catalyzed by transition metal complexes. A proposed mechanism has been outlined in Scheme 33B. The alcohol is first dehydrogenated to the corresponding aldehyde, followed by self-aldol condensation of the formed aldehyde to form an enal (crotonaldehyde) and subsequent hydrogenation of both C=C and the aldehyde group of the enal (crotonaldehyde) to form a higher-chain alcohol. Several heterogeneous^286,287^ and homogeneous catalysts have been reported in recent years for catalytic upgradation of ethanol to butanol using a “hydrogen borrowing” process. With relevance to the current report, we discuss the details of homogeneous catalysts, in particular pincer complexes, for ethanol to butanol transformation.

A homogeneous catalyst based on iridium was reported by Ishii in 2009 for the upgradation of ethanol to butanol.^288^ Using EtONa in the presence of [Ir(cod)(acac)] (**Ir8**) (acac = acetylacetonate, cod = 1,5-cyclooctadiene), with 1,3-bis(diphenylphosphino)propane (dppp) ligand and 1,7-octadiene as additive, *n*-butanol was obtained with selectivity up to 61% but at a lower conversion of 12%. Later, in 2013, the Wass group reported that using a ruthenium complex, [*trans*-RuCl_2_(dppm)_2_] (**Ru21**) (dppm = 1,2-bis(diphenylphosphino)methane) (0.01 mol %), and EtONa (5 mol %) at 150 °C and 20 h, 13% overall conversion of ethanol was obtained with 90% selectivity to *n*-butanol (TON = 1330).^289^ The same group also reported a more active system with *in situ* generated catalysts from a mixture of [RuCl_2_(η^6^-*p*-cymene)]_2_ and 2-(diphenylphosphino)ethylamine. With the use of 0.1 mol % catalyst loading and EtONa base (5 mol %), a good overall conversion of ethanol was obtained exhibiting high selectivity (≥92%). The isolated active catalyst [RuCl(η^6^-*p*-cymene)(2-(diphenylphosphino)ethylamine)]Cl (**Ru22**) showed almost the same reactivity.^290^

**Scheme 34:**
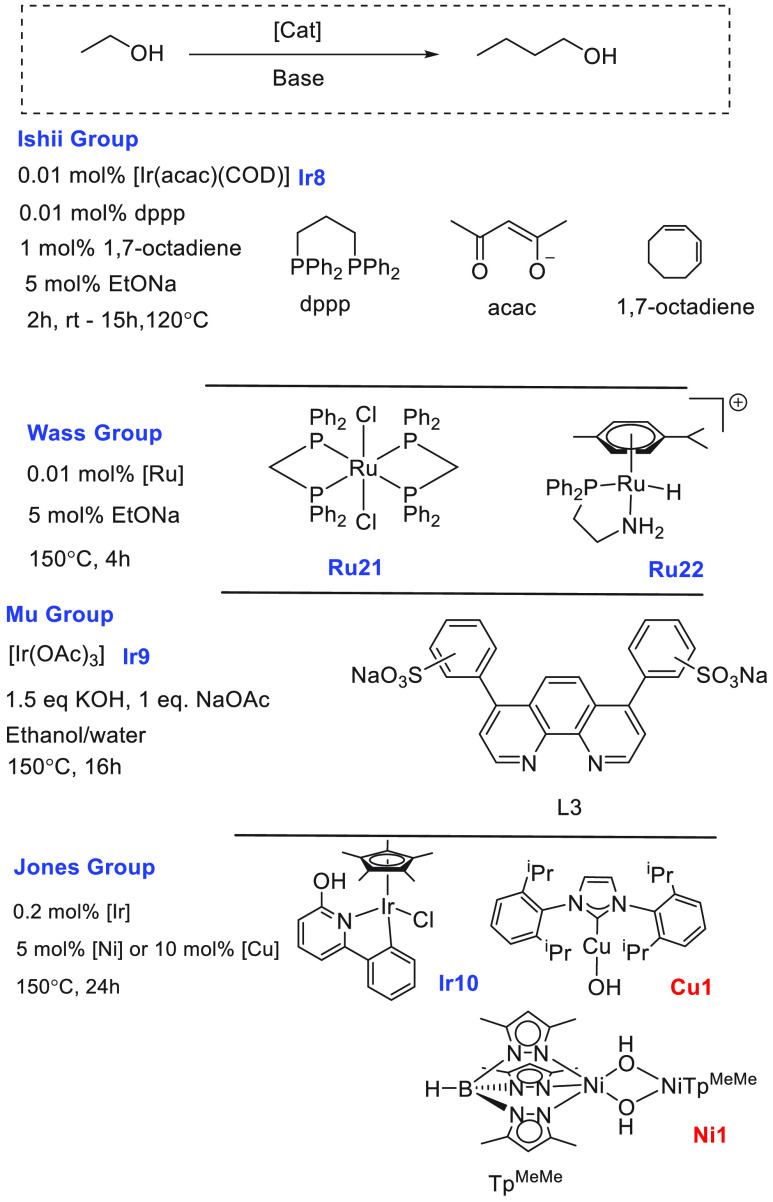
Guerbet Reaction Catalyzed by Homogeneous (Nonpincer) Catalysts Tp^MeMe^ = tris(3,5-dimethyl-pyrazolylborate).

Around the same time, Mu reported that, using [Ir(OAc)_3_] (**Ir9**) and bathophenanthroline disulfonic acid disodium salt as the ligand (**L3**), KOH, and NaOAc, resulted in 52% conversion of ethanol with 26% yield of *n*-butanol at 150 °C for 16 h in air.^291^ Interestingly, Jones demonstrated that, using highly basic, bulky complexes, the nickel complex **Ni1**, or the copper complex **Cu1** instead of bases such as NaOEt, excellent selectivity of butanol can be obtained.^292^ The high yield/selectivity was attributed to the sterically bulky nickel or copper hydroxide complexes that catalyze the aldol reaction in almost quantitative yield and selectivity to form the crotonaldehyde. The tandem catalytic reaction was performed in a combination of the iridium complex (**Ir10**) used for the catalytic (de)hydrogenation steps and **Ni1** or **Cu1** complexes used for the catalytic aldol reaction (Scheme 34).

**Scheme 35:**
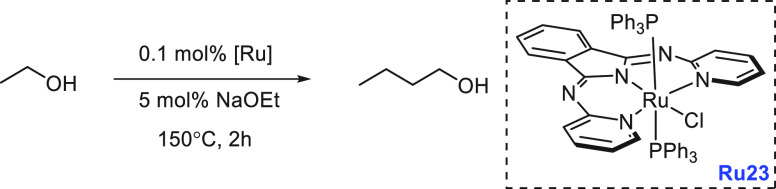
Ethanol to *n*-Butanol Formation Using Ru Pincer Complex

Along this direction, the Szymczak group in 2016 demonstrated an ethanol upgrade using an air-stable pincer-based complex **Ru23** (Scheme 35).^293^ They reported that, by heating 17.1 mmol of EtOH, 5 mol % NaOEt base, and 0.1 mol % Ru pincer catalyst (**Ru23**) at 150 °C for 2 h, 30% overall conversion of ethanol to *n*-butanol (91% selectivity, with TON = 300) was obtained. Significantly, performing the reaction with air-saturated solvents and weighing all the reagents in the air did not affect the reactivity of the system. The TON reached up to 1400 with 100% selectivity at 1.4% conversion upon decreasing the catalyst loading to 0.001 mol %. Detailed mechanistic studies suggested that displacement of PPh_3_ ligand and formation of mono- or dicarbonyl species slows down the reaction rate which could be avoided by adding excess PPh_3_ ligand during catalysis.

**Scheme 36:**
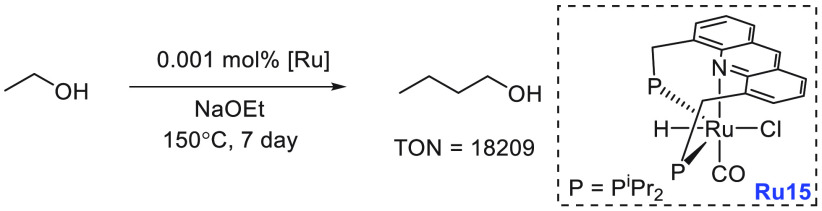
Ethanol Upgrading Using an Acridine-Based Ruthenium Complex

A record TON for the ethanol-to-butanol transformation was reported by Milstein in 2016 using an acridine-based ruthenium complex (**Ru15**, Scheme 36).^294^ A high yield and selectivity were obtained at a temperature of 150 °C using EtONa as a base. Interestingly, C_6_ and C_8_ alcohols, which have higher energy capacities than butanol, were also formed as coproducts via cross-coupling and homocoupling of 1-butanol. A maximum TON of 18 209 was obtained after 7 days.

**Scheme 37:**
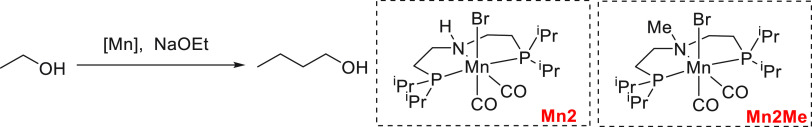
Guerbet Reaction of Ethanol-to-Butanol Transformation Using Mn Pincer Complexes

In addition to precious metal complexes, a few base-metal catalysts have also been recently reported for upgradation of ethanol to butanol. The first example of a base-metal catalyst for this transformation was reported by Liu in 2017 using a manganese pincer complex (**Mn2**) and NaOEt base (Scheme 37).^295^ Remarkably, a very high catalytic activity was observed and by using 0.0001 mol % (8 ppm) **Mn2**, a record TON of 114 120 with 92% butanol selectivity was obtained in 7 days (Scheme 37). Soon after, the Jones group also reported the same transformation using the **Mn2** complex (0.5 mol %) and NaOEt base (25 mol %).^296^ A lower amount of base loading (e.g., 50 equiv of EtONa relative to **Mn2**) was used in comparison to that reported by Liu (600 equiv of EtONa relative to **Mn2**), which could be responsible for a higher TON in Liu’s case. Interestingly, both groups reported a lower TON while using **Mn2Me** instead of **Mn2**, suggestive of the important role of N–H proton in catalysis. A detailed theoretical investigation of this process using a Mn(I)-PNP pincer complex has been reported by Pathak.^297^

**Scheme 38:**
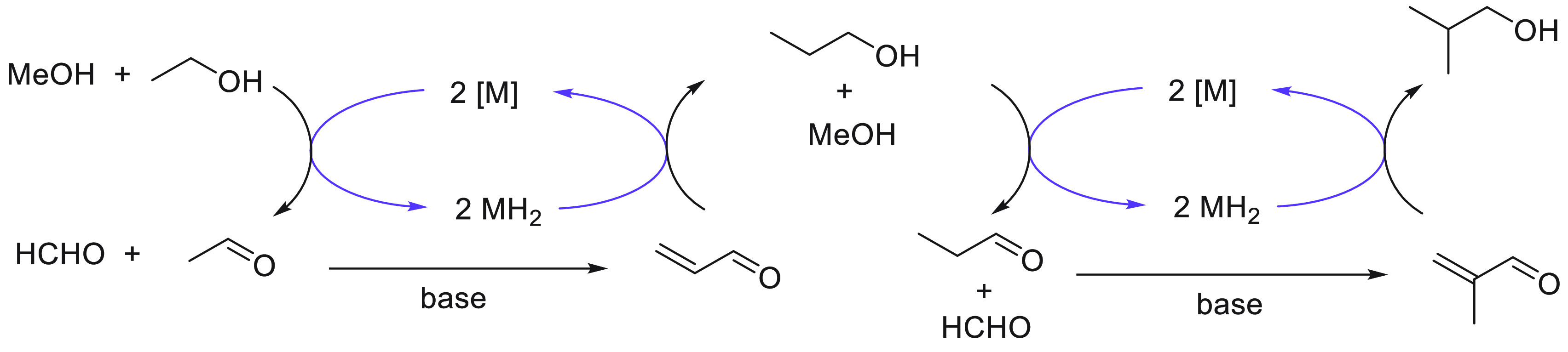
Synthesis of Isobutanol Using Ethanol and Methanol

Another attractive direction for ethanol upgradation is the conversion of ethanol to isobutanol, which has an improved octane number over 1-butanol. Ethanol can be coupled with methanol via a “hydrogen-borrowing” pathway in the presence of transition metal catalysts to produce isobutanol via a mechanism (Scheme 38) analogous to the ethanol-to-butanol transformation (Scheme 33B). As outlined in Scheme 38, ethanol and methanol first are dehydrogenated to form acetaldehyde and formaldehyde, respectively, followed by their aldol coupling reaction to form acrylaldehyde which subsequently is hydrogenated to form propanol. Propanol and methanol undergo the same consecutive cycle to form isobutanol. A few heterogeneous catalysts have been reported for this transformation, but they suffer from limitations such as harsh reaction conditions and low selectivity.^298−301^

**Scheme 39:**
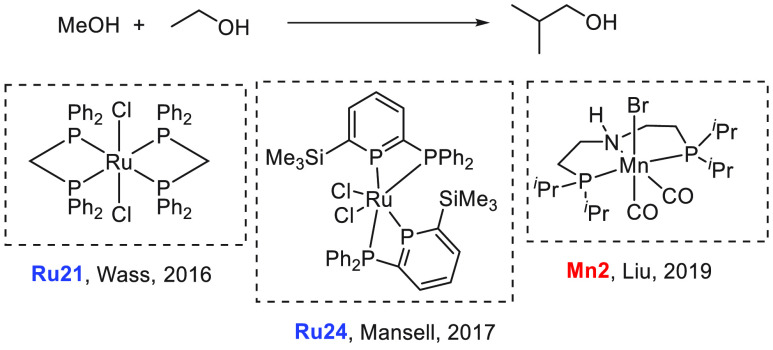
Isobutanol Formation from Ethanol and Methanol Using Transition Metal Complexes

A few homogeneous catalysts have also been investigated for the selective upgradation of ethanol to isobutanol. The first homogeneous catalyst in this direction was reported by the Wass group in 2016 using [RuCl_2_(L)_2_]-type complexes in the presence of a base such as NaOMe, where “L” is chelating diphosphine or PN ligands.^302^ The best performance was obtained by using the small-bite-angle dppm ligand based complex **Ru21** which resulted in up to 75.2% conversion and almost quantitative selectivity of isobutanol (Scheme 39). Later, the same group demonstrated that the Ru catalyst (**Ru21**) remains active in the presence of water of a concentration similar to that of fermentation broth.^303^ Interestingly, various commercial alcoholic beverages such as raki, lager, gin, brandy, and wine were used as ethanol surrogates obtained from fermentation broth instead of pure ethanol, exhibiting conversions of up to 79%. Mansell, in 2017, also reported a ruthenium complex bearing a chelating bis-phosphine ligand (**Ru24**) for the coupling of methanol and ethanol to form isobutanol in 50% yield and a selectivity of 96% (Scheme 39).^304^ Recently, in 2019, Xu, Yu, and Liu reported the upgradation of ethanol to isobutanol using the first base-metal catalyst based on manganese (**Mn2**, Scheme 39).^305^ Remarkably, a higher TON of up to 9233 (at 200 °C, 48 h) was obtained in comparison to those reported by Wass (TON = 750 at 180 °C, 20 h) and Mansell (TON = 495 at 180 °C, 20 h).

### 3.2 Hydrogenation of Vegetable Oils: Upgrading Biodiesel

Biodiesel is a type of diesel fuel compatible with the current infrastructure of distributing transportation fuel. Typically, biodiesel is a fatty acid methyl ester (FAME) that is usually produced from the transesterification of triglycerides derived from animal fat or plant oil with methanol.^306−308^ These FAMEs contain unsaturated fatty acid derivatives such as linoleic acid and linolenic acids. The direct use of such unsaturated FAMEs as biodiesels has several drawbacks such as lower fuel stability and lubricity, increased viscosity and gum formation, and slow ignition and high emission of hydrocarbons. To avoid these drawbacks, hydrogenation of unsaturated C=C has been performed using heterogeneous catalysts, under harsh reaction conditions.^309−312^

**Scheme 40:**
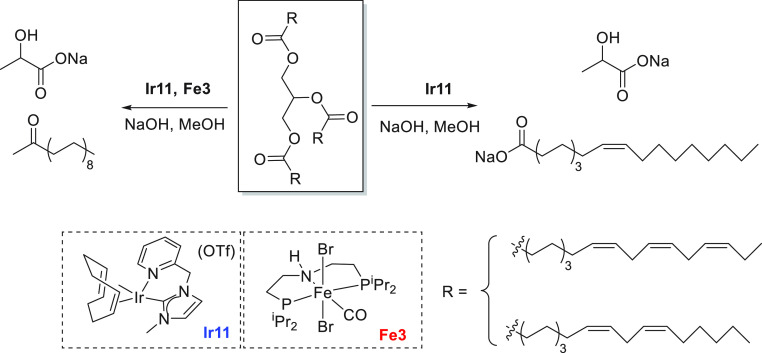
Upgrading Biodiesel via Hydrogenation of Vegetable Oils Using Homogeneous Ir and Fe Catalysts

Homogeneous catalysts have also been employed lately to produce (saturated) biodiesel from vegetable oil. The Williams group reported the transformation of a corn or soybean oil to biodiesel and lactate in the presence of 0.3 mol % **Ir11** and 5 equiv of NaOH in methanol or glycerol (Scheme 40).^313^ An advantage of this methodology over the conventional biodiesel production technology is that it produces lactate as a byproduct instead of glycerol, which is discarded as a waste from the biodiesel industry. A satisfying result was obtained using only 30 ppm **Ir11** catalyst, delivering over 230 000 turnovers in a single catalytic run. Interestingly, by using a combination of **Ir11** catalyst and the iron pincer catalyst **Fe3**, a reduction of unsaturated C=C was also obtained (product yield of up to 90%) without loss of selectivity in conversion of the backbone to lactate (Scheme 40). Also, the system exhibited good water tolerance, and the iridium catalyst was soluble in water which allowed smooth reusability of the catalyst.

### 3.3 Lignin Depolymerization

**Scheme 41:**
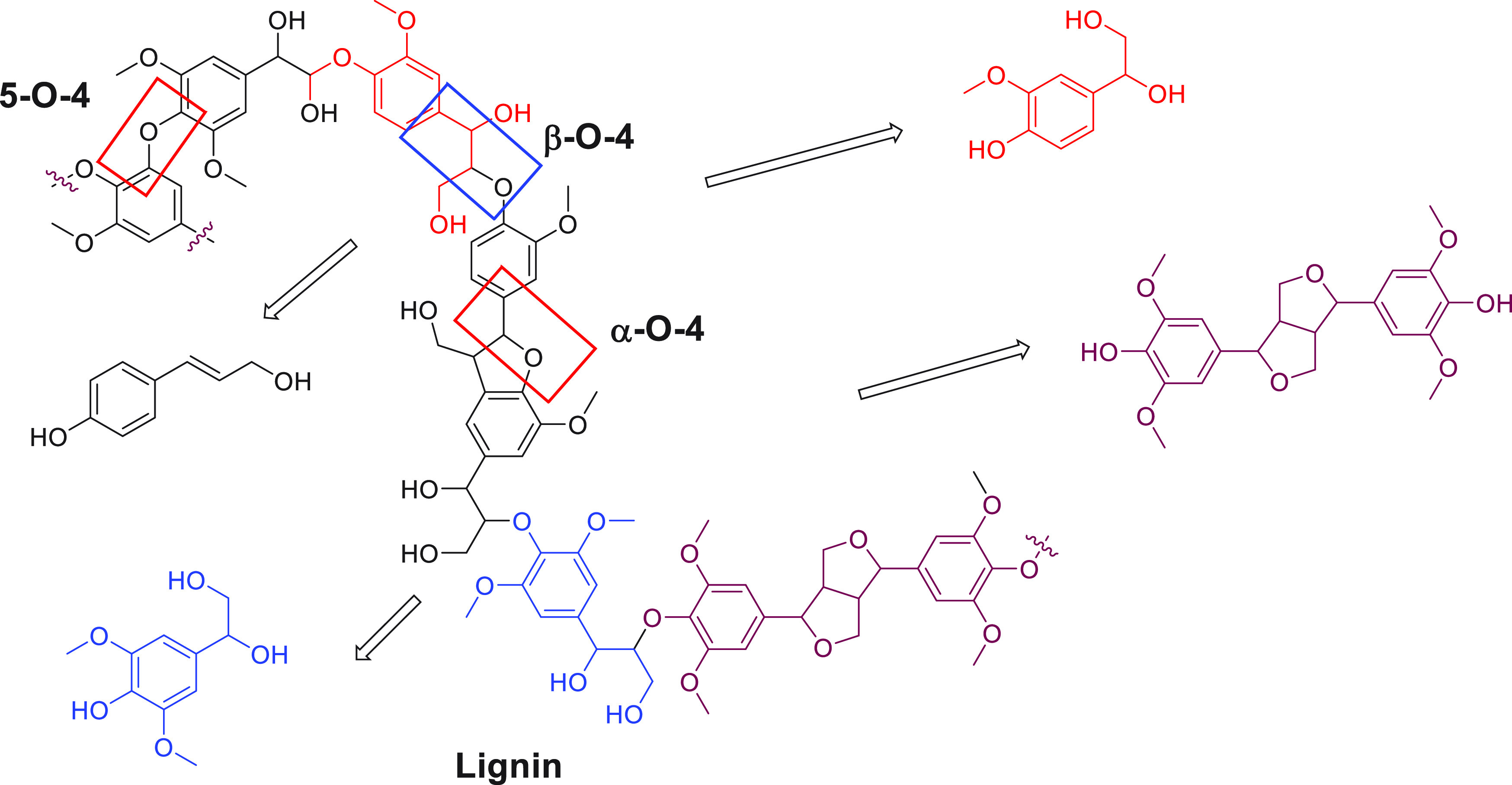
Polymeric Structure of Lignin Important bonds for the purpose of depolymerization are highlighted.

Lignin is a biomass-derived complex polymer containing aryl alkyl ether and alcoholic C–O linkages (Scheme 41). Lignin is usually considered a waste product from the pulp and paper industry and is produced at the scale of 70 million tons annually worldwide. Currently, lignin waste is mostly used to generate energy by burning, leading to harmful impacts on the environment.^314^ However, as lignin is made of organic fragments, its depolymerization can provide a sustainable route to access renewable and useful organic chemicals.

**Scheme 42:**
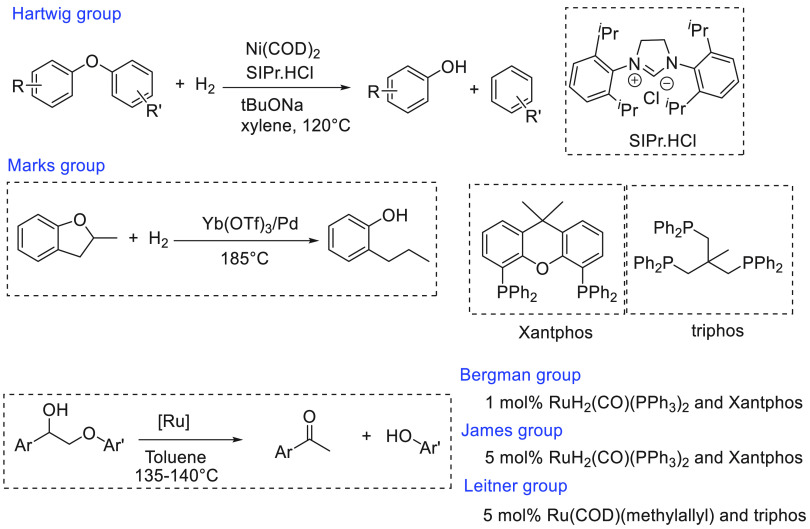
C–O Cleavage of Ethers Using Homogeneous Catalysts

In recent advances in this field, lignin has been depolymerized into oligomers containing phenolic fragments, also called lignin oil, which has applications in jet fuel and the polymer industry.^315^ However, the demonstration is only at the proof-of-concept level and there are certain challenges associated with the depolymerization of lignin on an industrial scale. These are due to (a) lack of a well-defined polymeric structure, making it difficult to develop a general protocol, and (b) difficulty in cleaving strong β-O-4 linkages (Scheme 41). Due to the need for harsh reaction conditions such as a high temperature, a vast majority of studies on the topic of lignin depolymerization involve heterogeneous catalysts or acid/base catalysts.^54−62^ However, recently a few examples of well-defined transition metal catalysts have been utilized for the reductive cleavage of the C–O bond in model ether compounds of lignin (Scheme 42).

A breakthrough in this direction was reported by the Hartwig group in 2011, who demonstrated selective cleavage of C–O bonds in aryl ethers using soluble catalysts consisting of Ni(COD)_2_ (5–20 mol %), and N-heterocyclic carbene ligand (10–40 mol %), in the presence of NaO^*t*^Bu (Scheme 42).^316^ Remarkably, only 1 atm H_2_ was sufficient for the cleavage in the 80–120 °C temperature range. Soon after, in 2012 and 2013, catalytic cleavage of cyclic alkyl ethers was reported by Marks using Yb(OTf)_3_/Pd nanoparticles and H_2_ through dehydroalkoxylation.^317,318^ Ellman and Bergman used the xantphos ligand with RuH_2_(CO)(PPh_3_)_3_ as a catalyst to cleave C–O bonds in alkyl aryl ethers such as 2-phenoxy-1-phenethanol, where the adjacent alcohol fragments transfer hydrogen to cleave the C–O bond.^319^ In this direction, a ruthenium–xantphos complex and a combination of [Ru(cod)(methylallyl)_2_] and the triphos ligand was reported by James^320^ and Leitner^321^ respectively for the cleavage of the same β-O-4 motif. All these catalysts used mild pressure of H_2_ (1–40 bar) or an internal hydrogen donor moiety at temperatures in the range 120–185 °C and produced moderate to excellent yields of the products.

**Scheme 43:**
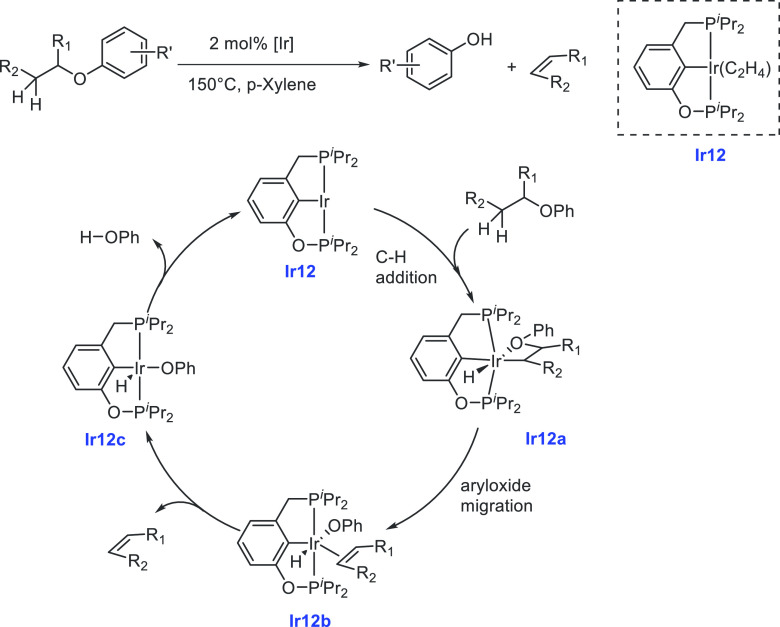
Cleavage of Ether C–O Linkage Using an Ir Pincer Complex

A new atom-economical approach without using any reductant was reported by Goldman. This was based on dehydroaryloxylation of alkyl aryl ethers using an Ir-based pincer catalyst.^322^ Various aryl ethers were successfully transformed to phenol and an olefin in the presence of 2 mol % (^*i*^PrPCOP)Ir (**Ir12**) at 150–200 °C for 16 h. Based on stoichiometric control experiments, a mechanism for the dehydroaryloxylation reaction was proposed as outlined in Scheme 43. The first step is the C–H activation of the alkyl aryl ether to form a four-membered cyclometalated complex (**Ir12a)**. This is followed by the migration of the aryl oxide moiety forming an olefin-bound complex **Ir12b** that liberates olefin to form **Ir12c** followed by the reductive elimination of phenol, regenerating **Ir12**. Direct depolymerization of lignin has also been attempted using a homogeneous ruthenium/xantphos catalyst; however, the catalytic activity was poor.^323^

## 4 Methanol Economy

Methanol is a highly important feedstock for several high-value chemicals and is globally produced at the scale of more than 75 million metric tons.^324^ Additionally, methanol has significant applications in the energy sector in internal combustion engines (ICE) and in direct methanol fuel cells (DMFC). The application of methanol as a hydrogen carrier for the hydrogen economy and homogeneous catalysts developed for the aqueous methanol reforming reaction has been discussed in section 2.1. Olah and Prakash have proposed the vision of a “methanol economy”,^26,64,325,326^ where methanol can be essentially produced in a renewable, sustainable, and carbon-neutral cycle by the carbon capture and recycling (CCR) process. In this section, we discuss homogeneous catalytic processes that have been developed for the sustainable production of methanol.

### 4.1 Methanol Production from CO_2_

Currently, methanol is industrially produced from syngas^327^ that is produced from fossil fuels, such as the gasification of coal.^328^ However, recently it has been possible to produce methanol from biomass or by the direct hydrogenation of CO_2_.^329^ Industrial production of 100% renewable methanol is carried out by Carbon Recycling International at the scale of 50 000–100 000 tons/year, where CO_2_ is captured from industrial emissions and then hydrogenated to methanol by using H_2_ produced from the electrolysis of water using renewable electricity.^330^ Most of the reports on direct hydrogenation of CO_2_ to methanol (CO_2_ + 3H_2_ → CH_3_OH + H_2_O) involve heterogeneous catalysts.^331−333^ Electrochemical reduction of CO_2_ to methanol using molecular complexes has also received significant attention.^334−337^

**Scheme 44:**
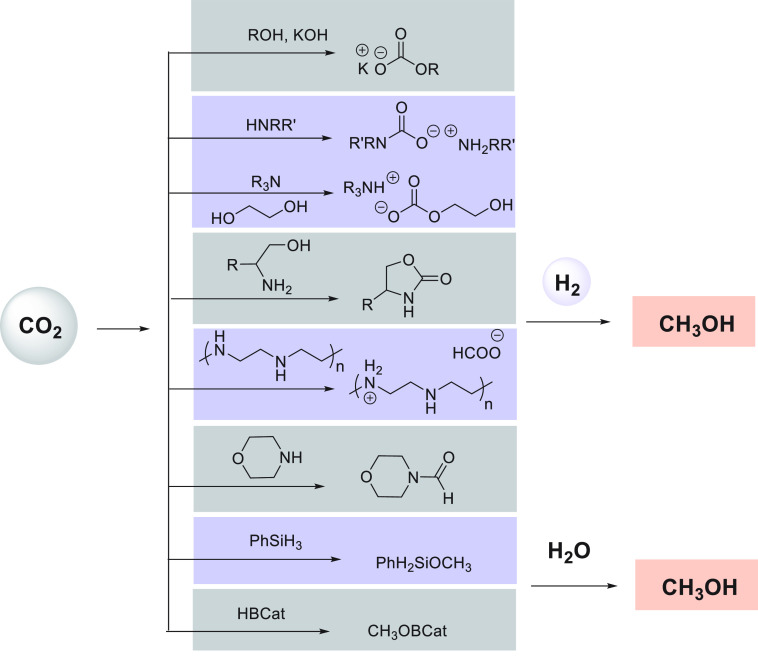
Two-Step Approach for the Conversion of CO_2_ to Methanol

Hydrogenation of CO_2_ to methanol is exothermic in nature (Δ*H*_r,298 K_ = −50 kJ/mol); however, high temperature is required to overcome the chemical inertness of CO_2_ that poses a thermodynamic hindrance as the reaction becomes thermodynamically less favorable at higher temperature. To overcome the thermodynamic challenge, an indirect approach for the conversion of CO_2_ to methanol has been developed using homogeneous catalysts under relatively mild conditions.^338−343^ In this approach, CO_2_ is first chemically captured by its reaction with a trapping reagent such as amines, alcohols, silanes, or boranes, followed by subsequent hydrogenation or hydrolysis to form methanol and regenerate the trapping reagent (Scheme 44). Section 4.1.1 describes various transition metal catalysts used for the indirect conversion of CO_2_ to methanol.

#### 4.1.1 CO_2_ to Methanol Using Amine as a Capturing Agent

**Scheme 45:**
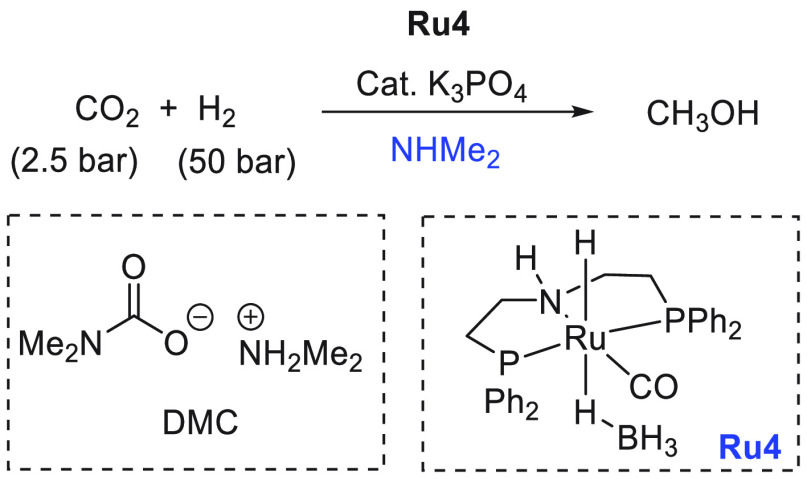
Amine-Assisted CO_2_-to-Methanol Hydrogenation Using a Ru Pincer Complex

Conventionally amines are used to capture CO_2_ as a carbamate salt from which CO_2_ can be liberated at a high temperature with concomitant regeneration of amines. However, a significant amount of amine is decomposed in the regeneration process, leading to the low efficiency of this process. As amine-assisted CO_2_ capture technology has already been developed, transformation of the trapped intermediate to methanol can be an attractive indirect approach for CO_2_ reduction to methanol. Amine-assisted CO_2_-to-methanol transformation was first reported by Sanford using NHMe_2_ and a pincer catalyst, **Ru4** (Scheme 45).^344^ CO_2_ is captured using NHMe_2_ to form dimethylammonium dimethylcarbamate (DMC) that can be subsequently hydrogenated by **Ru4** to form a mixture of methanol and dimethylformamide (DMF). With the use of a combination of Ru catalyst (**Ru4**) and K_3_PO_4_, CO_2_ was hydrogenated to a mixture of DMF/DMFA (dimethylammonium formate) and CH_3_OH. Overall, 96% conversion of CO_2_ and 27% yield of methanol were obtained.

**Scheme 46:**
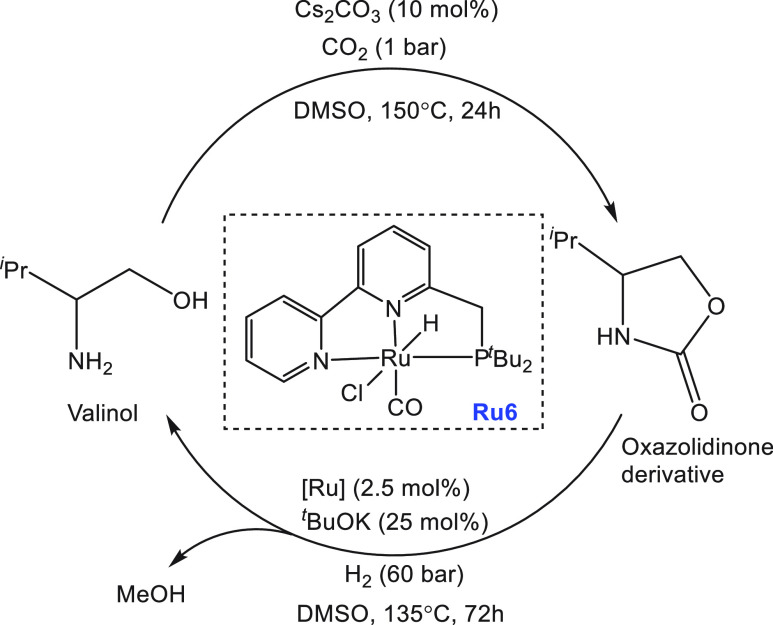
Aminoethanol-Assisted CO_2_ to Methanol Using Ru Pincer Complex

Around the same time, the Milstein group reported hydrogenation of low-pressure CO_2_ (1 atm).^345^ CO_2_ was captured using valinol to form an oxazolidinone-type intermediate followed by subsequent hydrogenation to methanol. CO_2_ capture was catalyzed by Cs_2_CO_3_,^346^ whereas the hydrogenation reaction was catalyzed by a ruthenium pincer complex **Ru6** in the presence of KO^*t*^Bu (Scheme 46). Furthermore, a tandem reaction was also demonstrated without the need for the isolation of the oxazolidinone derivative. Only 1 atm pressure of CO_2_ was sufficient for the formation of oxazolidinone derivative which was hydrogenated to form methanol at 60 bar and 135 °C, producing MeOH in 53% yield (Scheme 46).

**Scheme 47:**
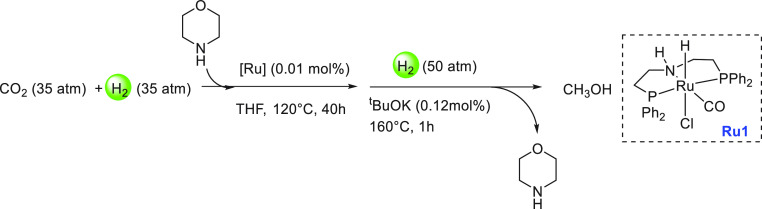
Morpholine-Assisted CO_2_ to Methanol Using Ru Pincer Complex

In a similar direction, Ding reported a sequential CO_2_ reduction to CH_3_OH using a Ru-MACHO catalyst, **Ru1**, in the presence of morpholine.^347^ Under the reaction condition (70 atm of 1:1 CO_2_:H_2_) CO_2_ was captured as *N*-formylmorpholine, which was subsequently hydrogenated to form methanol in 36% yield (Scheme 47).

**Scheme 48:**
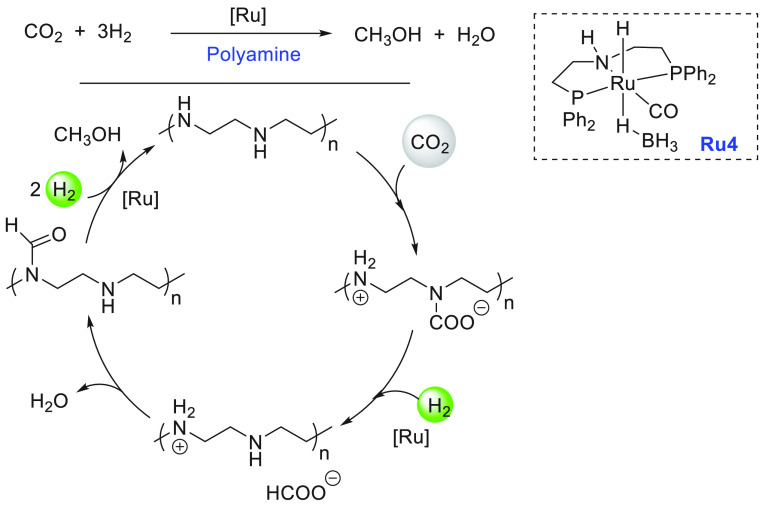
Polyethylenimine-Assisted CO_2_ to Methanol Using a Ru Pincer Complex

Prakash has recently reported a series of papers on CO_2_-to-methanol transformation using various additives. For example, in an interesting discovery, CO_2_ was hydrogenated to CH_3_OH in the presence of polyamines such as pentaethylenehexamine (PEHA; Scheme 48).^348^ Polyamines have higher basicities, allowing them to capture more CO_2_ than an amine molecule. Moreover, they exhibit higher thermal stabilities and lower volatilities, making them better candidates for CO_2_ capture. The ruthenium-MACHO-BH catalyst **Ru4** was utilized for the hydrogenation of CO_2_ at the pressure of 75 bar (CO_2_/H_2_ 1:3) and in the temperature range 95–155 °C in THF solvent. The addition of a catalytic amount of base (K_3_PO_4_) was found to increase the yield of methanol from 7.6 to 9 mmol presumably by favoring the N–H-assisted metal–ligand cooperation pathways. Recycling of catalyst was also demonstrated for five cycles exhibiting a total TON of 1850. A proposed mechanism that is similar to those using amine for CO_2_ capture is outlined in Scheme 48. The reaction starts with the trapping of CO_2_ by basic polyamines resulting in the formation of a carbamate salt that is hydrogenated to form a formate salt. The formate salt is dehydrated under the thermal condition to form a formamide that subsequently is hydrogenated in the presence of **Ru4** to form methanol and regenerate the polyamine.

**Scheme 49:**
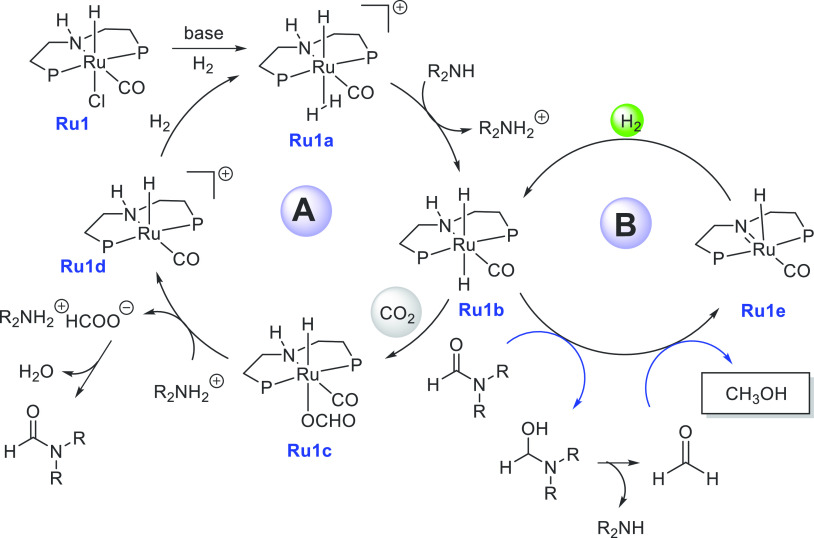
Proposed Mechanism of Amine-Assisted CO_2_-to-Methanol Transformation Using Ru Pincer Complex P = PPh_2_.

Later, Prakash reported a more practical approach of amine-assisted CO_2_-to-methanol transformation using a biphasic solvent system consisting of 2-MeTHF and H_2_O, considering that catalysts are mostly soluble in organic solvents whereas the capturing amines are soluble in water.^349^ This approach was helpful in the recycling of amines and catalysts after the hydrogenation step. Mechanistic studies were later reported by Prakash.^350^ Interesting structure–activity studies were conducted by the variation of phosphine substituents of the Ru-PNP complex, revealing that methanol yield followed the order Ph > ^*i*^Pr > Cy > ^*t*^Bu (phosphine substituents, PR_2_). Based on further experiments, a mechanism consisting of two cycles as outlined in Scheme 49 was proposed. Cycle A describes the formation of formamide from CO_2_, amine, and H_2_, whereas cycle B describes the hydrogenation of formamide to form methanol. The catalysis starts with cycle A where the dihydrogen sigma-complex **Ru1a**, formed from the reaction of precatalyst **Ru1** with base and H_2_, is deprotonated by an amine, forming a ruthenium *trans*-dihydride complex **Ru1b**. Insertion of CO_2_ to **Ru1b** results in the formation of the formyl complex **Ru1c**. Attack of an amine on the formyl moiety of **Ru1c** forms an alkylammonium formate salt and the cationic complex **Ru1d** that coordinates with H_2_ to regenerate the sigma-complex **Ru1a**. The formed alkylammonium formate salt is dehydrated under thermal conditions to form formamide that feeds to cycle B, where it is hydrogenated by **Ru1b** to form a hemiaminal intermediate and the complex **Ru1e**. **Ru1e** reacts with H_2_ to regenerate the *trans*-dihydride complex **Ru1b**. The hemiaminal intermediate decomposes to form formaldehyde that is hydrogenated by the same cycle to form methanol.

**Scheme 50:**
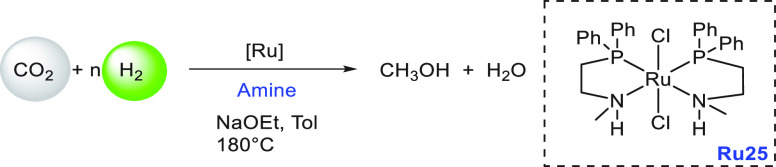
Amine-Assisted CO_2_-to-Methanol Formation Using Ru Non-Pincer Complex

Furthermore, Prakash reported a modified version of this process by immobilizing the amine onto a solid support.^351^ The advantage of solid-supported amines (SSAs) is that no solvent is needed, making the overall process less energy intensive. A nonpincer ruthenium complex **Ru25** was utilized by Wass for the hydrogenation of CO_2_ to methanol in the presence of an amine (Scheme 50).^352^ Similar to the mechanism described earlier, CO_2_ is trapped by an amine and H_2_ to generate a formamide that is hydrogenated by **Ru25** to form methanol, regenerating the amine. It was observed that the choice of amine plays a crucial role in the yield of methanol. Bulkier amines such as pyrrolidine, ^*i*^Pr_2_NH, and Et_2_NH afforded lower yields compared to that of Me_2_NH. No methanol was produced by using the tertiary amine NEt_3_ due to the lack of a N–H proton required for the generation of a formamide. Catalysis was performed at 180 °C, under 10 bar CO_2_ and 30 bar H_2_ pressure, exhibiting a TON up to 8900 and a TOF up to 4500. Interestingly, no methanol was formed when the catalysis was performed using an analogous ruthenium complex containing a tertiary amine moiety, suggesting the crucial role of the N–H proton of the ligand in catalysis. It was suggested that the catalytic hydrogenation occurs via an “outer-sphere mechanism” facilitated by metal–ligand cooperativity.

**Scheme 51:**
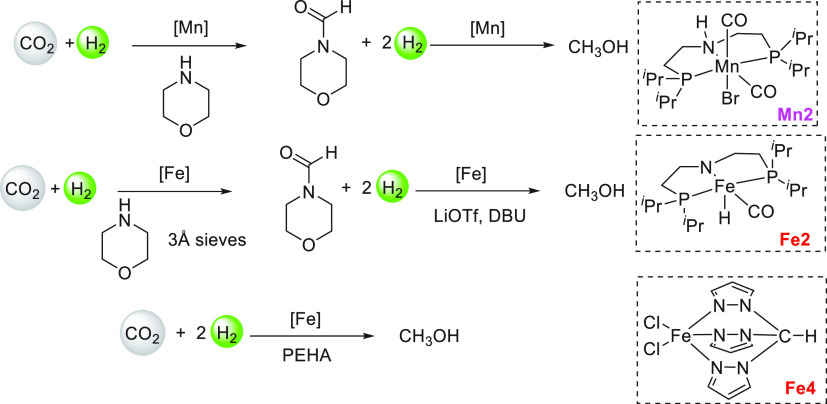
Amine-Assisted CO_2_-to-Methanol Transformation Using Mn and Fe Complexes

In addition to precious metal complexes, base-metal complexes were also employed for the integrated conversion of CO_2_ to CH_3_OH (Scheme 51). The Prakash group employed a manganese pincer catalyst, **Mn2**, for the conversion of CO_2_ to CH_3_OH using amines such as morpholine or benzylamine (Scheme 51).^353^ The CO_2_ is trapped as an N-formylated product that is hydrogenated to produce methanol. Compared to the ruthenium catalysts described above, the TON was found to be significantly lower (up to 36). Using a similar approach, the Bernskoetter group recently reported the hydrogenation of CO_2_ to methanol catalyzed by an iron pincer catalyst, **Fe2**, using morpholine as an amine source (Scheme 51).^354^ The initial step involves N-formylation of morpholine using CO_2_/H_2_ to produce formylated morpholine and is followed by deaminative hydrogenation in the presence of an iron pincer catalyst to form methanol. Overall, a high TON of 1160 was obtained for the reaction of CO_2_ and H_2_ to form formylmorpholine, and a TON of 590 was achieved for the hydrogenation of isolated formylmorpholine to methanol. Mechanistic studies suggest that the presence of CO_2_ inhibits the hydrogenation of formamide by forming a stable iron(II) formate complex, thus hampering the single batch catalysis. Along this direction, Martins and Pombeiro have reported an iron catalyst exhibiting a TON up to 2300 for the hydrogenation of CO_2_ to methanol using pentaethylenehexamine.^355^ Drawing inspiration from the metal–ligand cooperation exhibited by [FeFe] hydrogenase enzymes, the authors used the tripodal C-scorpionate iron(II) complex (**Fe4**) as a catalyst (Scheme 51). It is expected that the N-atoms present in the pyrazolyl rings assist in proton transfer needed for the H–H cleavage step.

#### 4.1.2 CO_2_ to Methanol Using Alcohols

**Scheme 52:**
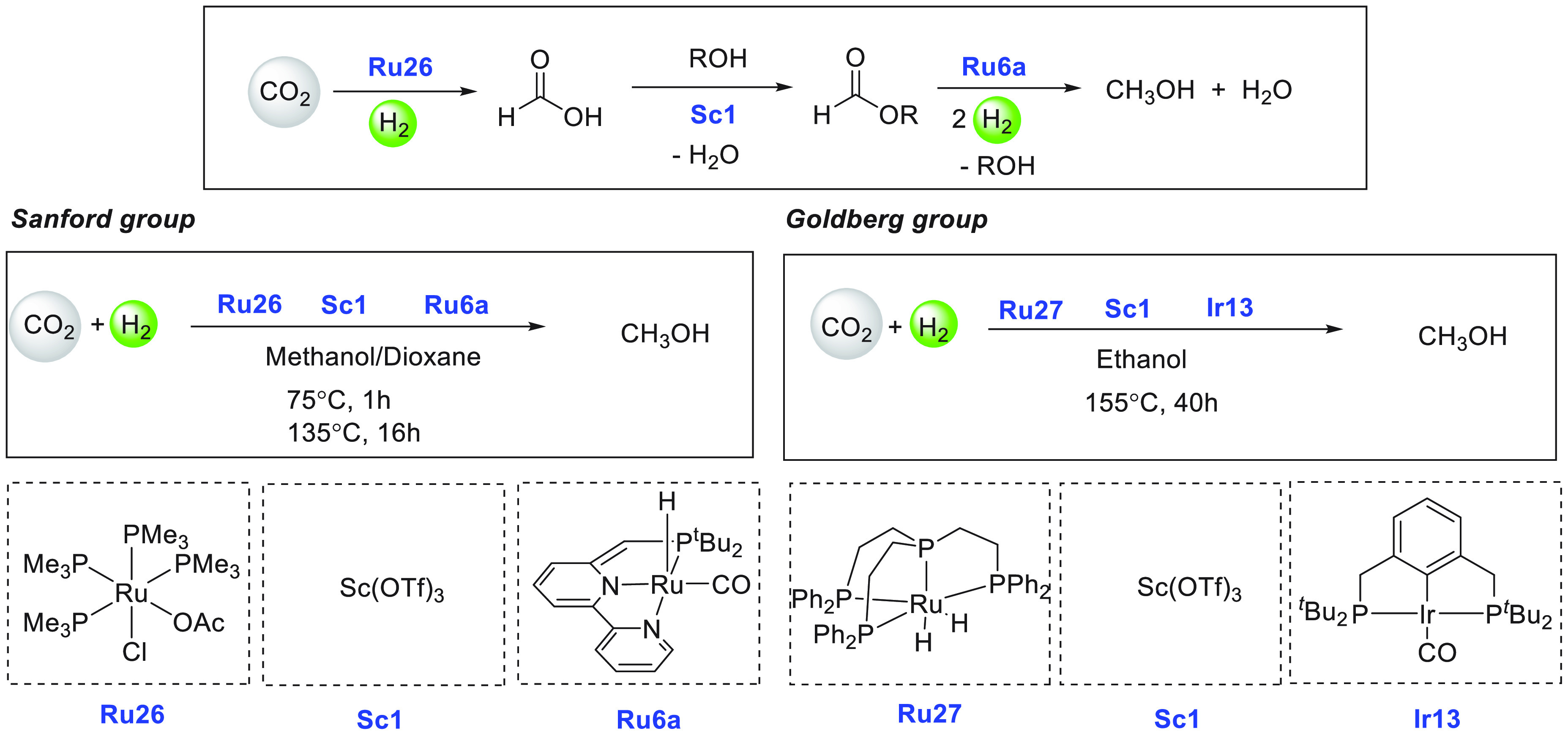
Alcohol-Assisted CO_2_-to-Methanol Transformation via Cascade Catalysis

In addition to amines, alcohols have also been employed for the reduction of CO_2_ to CH_3_OH (Scheme 52). The first example in this direction was reported in 2011 by the Sanford group using a cascade catalytic process.^356^ The cascade catalysis consisted of (a) hydrogenation of CO_2_ to HCOOH catalyzed by **Ru26**, (b) Sc(OTf)_3_ catalyzed reaction of HCOOH with methanol to form methyl formate (HCOOMe), and (c) hydrogenation of HCOOMe catalyzed by Milstein’s catalyst (PNN)Ru-(CO)(H) (**Ru6a**) to form methanol. To demonstrate the proof of concept, a reaction of 30 bar CO_2_ and 10 bar H_2_ was performed using 0.0126 mmol each of the catalysts **Ru26**, Sc(OTf)_3_ (**Sc1**), and **Ru6a**, which resulted in the formation of a mixture of CH_3_OH (2.5 turnovers) and HCO_2_CH_3_ (34 turnovers). The low yield of CH_3_OH was attributed to the deactivation of Milstein’s catalyst (**Ru6a**) by Sc(OTf)_3_. This issue was solved by physically separating the **Ru6a** catalyst from the remaining two catalysts, which were stored in a vial in the central area of the reactor, while the PNN complex **Ru6a** was stored in the outer wall of the autoclave. The *in situ* generated methyl formate that formed in the central vial was transferred to the outer vessel under the reaction condition and was hydrogenated in the presence of **Ru6a**. This approach was indeed successful, and a higher yield of methanol (21 turnovers) was obtained.

Goldberg has recently reported a more efficient catalytic system using a similar approach.^357^ Screening of several catalysts revealed that the combination of **Ru27**/**Sc1**/**Ir13** was the most active for the hydrogenation of CO_2_ to methanol in the presence of ethanol. A TON of 428 (40 h, 155 °C) was obtained under the catalytic condition producing a high yield of methanol (1.07 M).

Byers and Tsung have recently reported a bio-inspired multicomponent catalytic approach for the hydrogenation of CO_2_ to methanol in the presence of alcohol.^358^ The cascade catalytic process involves two catalysts: (a) a RuPNP pincer catalyst encapsulated in an MOF (UiO-66) that converts CO_2_ to a formate ester via HCOOH in the presence of alcohol and (b) Milstein’s catalyst **Ru6** (Table 2) that hydrogenates the formate ester to methanol and regenerates the alcohol. The heterogeneous catalyst system was successfully recycled, demonstrating an excellent TON of up to 21 000 at the end of the five cycles. The same groups have recently studied the effect of the secondary sphere interaction between the encapsulated pincer catalyst and the MOF host on the catalytic hydrogenation reaction.^359^ A variety of functionalized MOF (UiO-66-X) hosts was used to study the structure–activity relationship. The best results were obtained using the ammonium functional group (UiO-66-NH_3_^+^) that resulted in a significantly higher TON of up to 19 000. The catalyst recyclability was also demonstrated with a TON of up to 100 000 at the end of 10 cycles. The high activity was attributed to the enhanced rate of the hydrogenation of CO_2_ to HCOOH which was accelerated due to the presence of ammonium moiety.

**Scheme 53:**
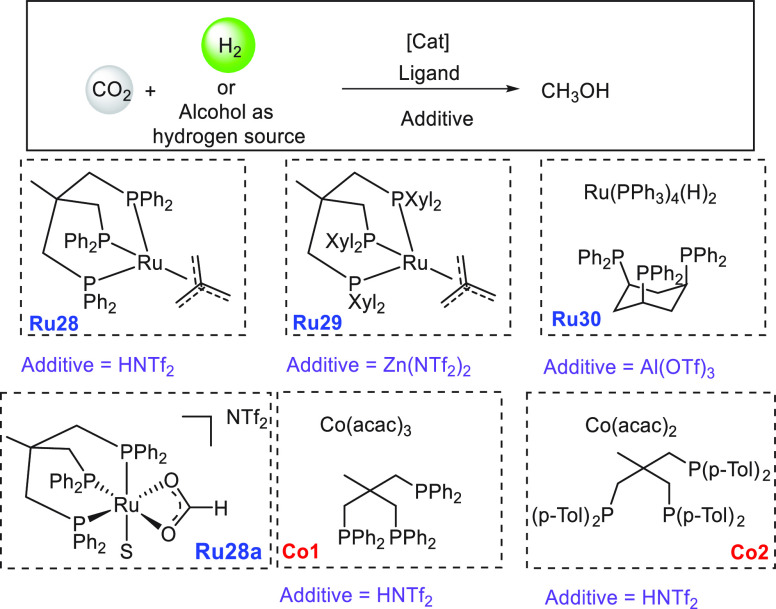
Hydrogenation of CO_2_ to CH_3_OH Using Ruthenium- and Cobalt-Triphos Catalysts

In addition to pincer catalysts, systems based on triphos ligands have also been studied for the conversion of CO_2_ to CH_3_OH in the presence of alcohol additives. A key feature of such systems is that they operate under acidic conditions, making them distinct from most of the pincer catalysts that operate under basic or neutral conditions. Klankermayer, Leitner, and co-workers, in 2012, reported the ruthenium-triphos catalyst **Ru28** in combination with the acid cocatalyst bis(trifluoromethane)sulfonimide (HNTf_2_) for the efficient hydrogenation of CO_2_ to CH_3_OH (Scheme 53).^360^ Ethanol was used as an additive to trap the formic acid as ethyl formate that could be easily hydrogenated. Mechanistic studies reported later by the same group revealed that HNTf_2_ reacts with **Ru28** to form a cationic species **Ru28a** that was proposed to be an active species in the catalytic process.^361^

In addition to direct hydrogenation, transfer hydrogenation has also been reported recently by Klankermayer using a combination of [Ru(triphosXyl)(tmm)] (**Ru29**, Scheme 53) and the Lewis acid Zn(NTf_2_)_2_.^362^ Linear alcohols such as ethanol were used as the hydrogen source as well as to stabilize formic acid by forming ethyl formate and a TON of up to 121 was achieved under relatively mild conditions. Very recently the same group also demonstrated a highly active cyclohexyltriphosphine ligand based ruthenium catalyst (**Ru30**, Scheme 53) system for the hydrogenation of CO_2_ to methanol using aluminum tristriflate (Al(OTf)_3_) as a Lewis acid additive.^363^ Under the optimized conditions of H_2_/CO_2_ = 90/30 bar pressure, 120 °C, and 20 h reaction time, a TON of up to 2100 was obtained in ethanol solvent. Moreover, the reduction of CO_2_ to methanol was also performed in a biphasic mixture consisting of *n*-decanol and water with the same system under the optimized reaction condition with maximum TONs up to 1087.

In addition to ruthenium-triphos systems, base-metal triphos catalysts were also utilized for the conversion of CO_2_ to methanol. Beller’s group utilized the Co(acac)_3_/triphos-based system (**Co1**, Scheme 53) in the presence of HNTf_2_ as an additive for the hydrogenation of CO_2_ to methanol.^364^ A TON of up to 50 was obtained under the condition of 70 bar H_2_ and 20 bar CO_2_ at 100 °C. Later the same group also reported a more effective cobalt catalyst for the hydrogenation of CO_2_ to CH_3_OH using a cobalt complex based on a modified triphos ligand (**Co2**, Scheme 53).^365^ The *p*-toluene substituted triphos ligand system with Co(acac)_2_ and HNTf_2_ as an additive led to TONs up to 125. Remarkably, the system also worked efficiently under additive-free conditions by replacing the metal precursor with Co(NTf_2_)_2_.

In addition to methanol and ethanol, alkali metal hydroxide solutions have also been utilized for the integrated capture and transformation of CO_2_ to methanol. Alkali metal hydroxide solutions have several advantages such as higher abundance, higher stability, and higher efficiency for CO_2_ capture from the air in comparison to amines.^366^

**Scheme 54:**
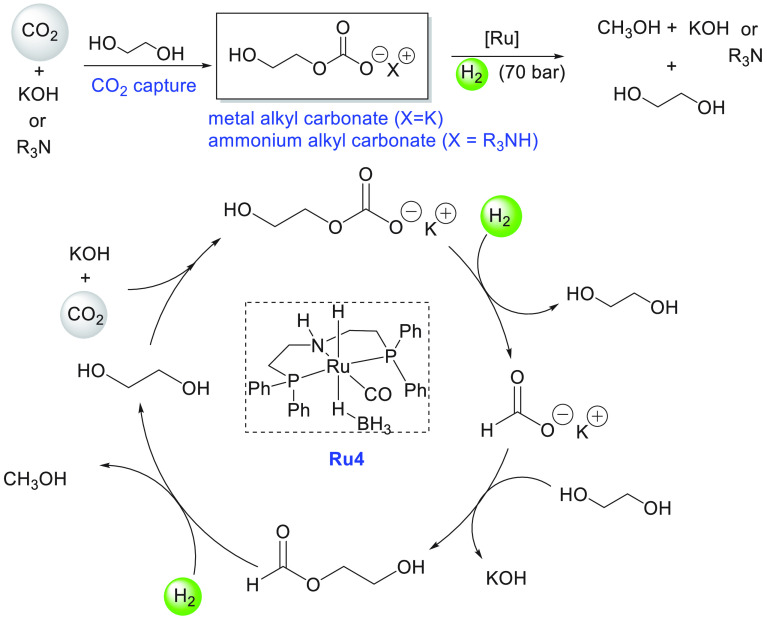
CO_2_-to-Methanol Conversion Using Alkali Metal Hydroxide or Tertiary Amine as a CO_2_ Scrubbing Agent

Recently, Prakash reported the use of a solution of an alkali metal hydroxide such as KOH for CO_2_ capture, and ethylene glycol as formate ester stabilizer, to convert CO_2_ to CH_3_OH catalyzed by a ruthenium pincer catalyst, **Ru4**.^367^ CO_2_ was captured as carbonate and alkyl carbonate salt by bubbling air through the KOH solution. This was treated with 0.5 mol % (**Ru4**) catalyst and 70 bar H_2_ at 140 °C, which resulted in a methanol yield of 25% within 20 h and a quantitative yield after 72 h. A mechanistic pathway was speculated based on catalytic experiments (Scheme 54). The metal alkyl carbonate salt produced from the reaction of CO_2_, ethylene glycol, and KOH reacts with H_2_ to form ethylene glycol and potassium formate salt. HCOOK reacts with the generated ethylene glycol to form 2-hydroxyethyl formate that is subsequently hydrogenated to methanol, regenerating ethylene glycol.

Recently, Prakash has expanded this concept and reported a tertiary amine–ethylene glycol based integrated CO_2_ capture and hydrogenation process.^368^ The combination of a tertiary amine and ethylene glycol was found to efficiently capture CO_2_ to form an ammonium alkyl carbonate intermediate that subsequently is hydrogenated in the presence of **Ru4** catalyst to produce methanol. Of several screened tertiary amines in combination with ethylene glycol, tetramethylethylenediamine and tetramethylbutanediamine were found to afford the best yields of methanol (up to 94% using 0.5 mol % **Ru4**) upon CO_2_ capture/hydrogenation from a gas mixture containing 10% CO_2_, similar to that found in the flue gas. Although the proof of concept was successfully demonstrated, the TON was similar to that of the earlier case where ethylene glycol in combination with KOH was used and CO_2_ could be directly captured from air (Scheme 54).

#### 4.1.3 CO_2_ to Methanol Using Silanes/Boranes

**Scheme 55:**
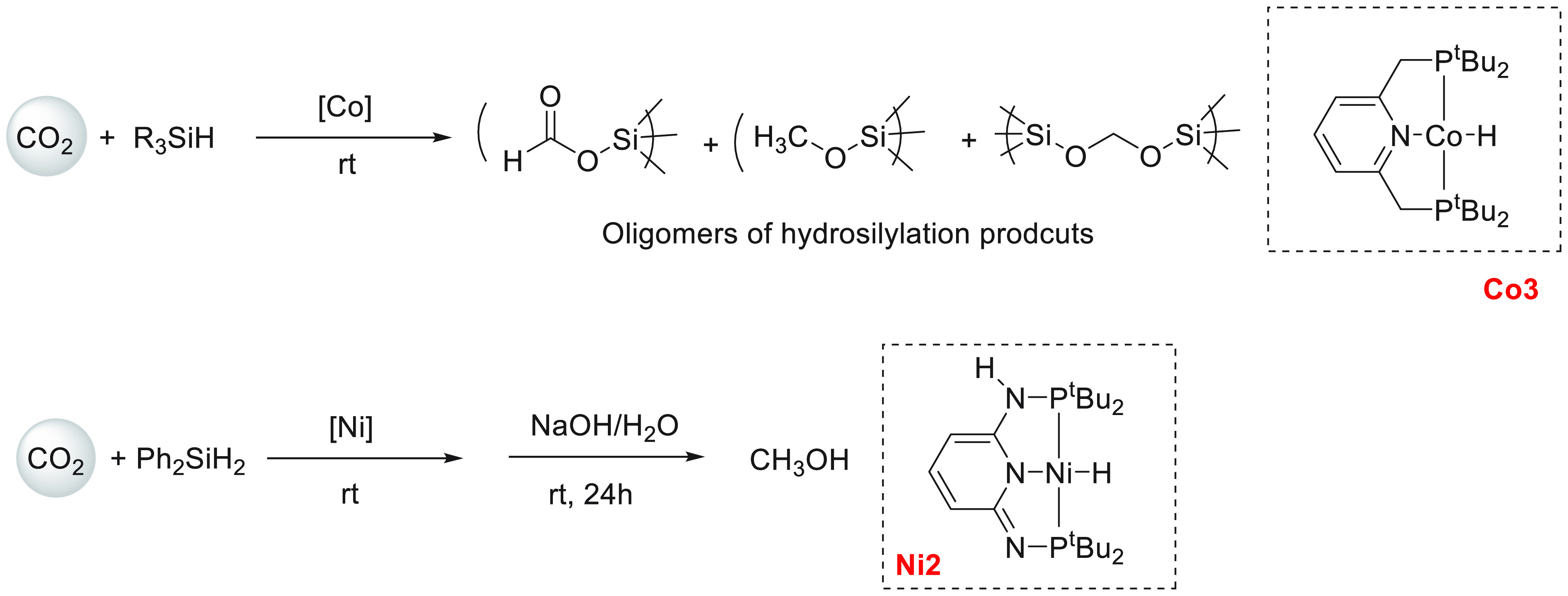
Hydrosilylation of CO_2_ Using Co and Ni Pincer Complexes

In addition to molecular hydrogen, reducing agents such as silanes and boranes have also been employed for the transformation of CO_2_ to CH_3_OH. The silylated or boranated products, formed from the reaction of CO_2_ with silanes or boranes, respectively, can be easily hydrolyzed to form methanol. Their advantages compared to hydrogenation reactions are that (a) they are easy to handle, (b) reactions can be performed under mild conditions, and (c) the reaction is thermodynamically more favorable because of the formation of strong Si–O or B–O bonds. Catalysts based on main group^369−371^ and transition metals^372−377^ have been studied in the past for the conversion of CO_2_ to methoxysilyl derivatives. Regarding transition metal pincer complexes, the Chirik group reported a cobalt pincer catalyst, **Co3**, for the hydrosilylation of CO_2_ using PhSiH_3_ that produced a mixture of oligomers (Scheme 55).^378^ Along this line, the Huang group reported the reduction of CO_2_ (1 atm) in DMF using Ph_2_SiH_2_ as a reductant and the dearomatized PN3P*-Ni hydride complex **Ni2** as a catalyst (Scheme 55).^379^ Methanol was obtained in excellent yield (e.g., 91%) from the hydrolysis of hydrosilylation product.

**Scheme 56:**
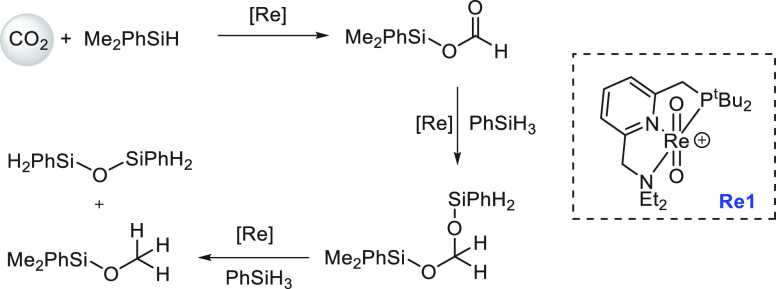
Hydrosilylation of CO_2_ to Silyl Methanol Using a Re Pincer Catalyst

Similarly, Abu-Omar reported a rhenium pincer catalyst **Re1** for the reduction of CO_2_ (100 psi) using Me_2_PhSiH (Scheme 56).^380^ The reaction initially produced silyl formate HC(O)(OSiMe_2_Ph), which was further reduced by the addition of a primary silane, PhSiH_3_. Methoxysilane was obtained in 53% yield from silyl formate, via the silyl formal intermediate, by treatment of excess PhSiH_3_ for 24 h using catalyst **Re1** (Scheme 56).

**Scheme 57:**
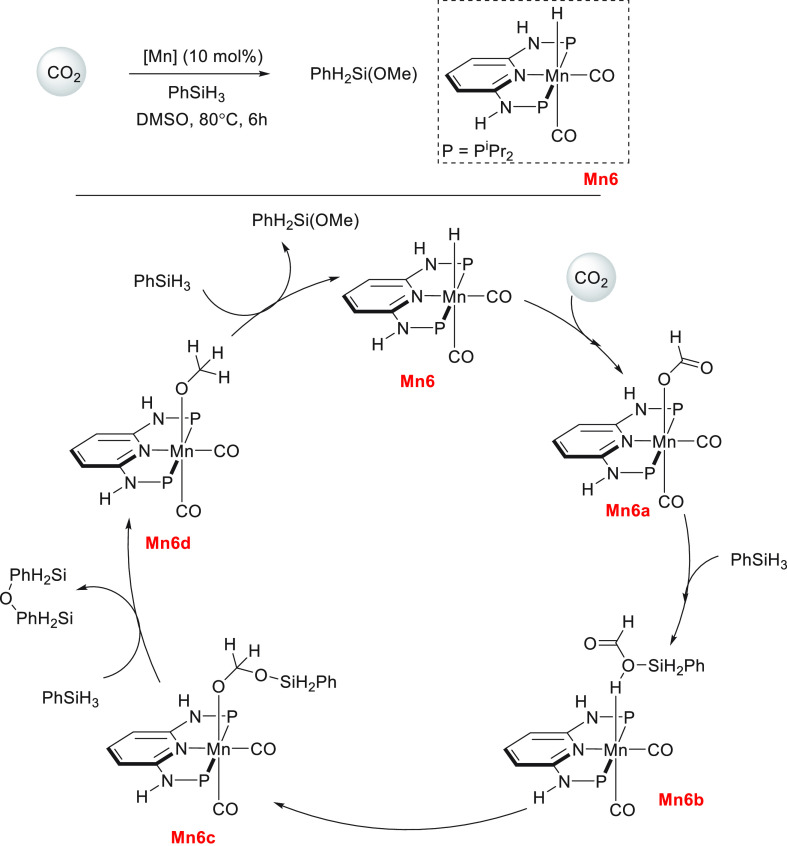
Manganese Pincer Complex Catalyzed Hydrosilylation of CO_2_ to Methoxysilane and a Proposed Mechanism

Along the direction of base-metal catalysis, Kirchner and Gonsalvi reported manganese catalyzed hydrosilylation of CO_2_ to methoxysilane under mild conditions (80 °C, 1 bar CO_2_).^381^ Catalysis was performed with [Mn(^^*i*^Pr^PN3P)(CO)_2_H] (**Mn6**) at room temperature, resulting in the rapid formation of silylformates, which convert to methoxysilyl products over time (93% in 46 h), whereas performing the reaction at a higher temperature, e.g., 80 °C, shifts the selectivity to methoxysilyl products (99% in 6 h). A mechanism of this reaction as elucidated experimentally and by DFT calculations is outlined in Scheme 57. First, Mn–H attacks CO_2_ to form a metal-coordinated formate intermediate, **Mn6a**, which undergoes a reaction with silane to form the silyl formate Mn hydride complex **Mn6b**. The formation of **Mn6b** from **Mn6a** has the highest barrier in the entire cycle. Attack of the hydride ligand to the C=O bond of the attached formate moiety in complex **Mn6b** forms a coordinated silyl hemiacetal, **Mn6c**. This is followed by the attack of silicon from a second PhSiH_3_ molecule to the OSiH_2_Ph moiety of complex **Mn6c** resulting in the release of (PhSiH_2_)_2_O, forming the methoxy complex **Mn6d**. Finally, attack of the third silane molecule to the coordinated methoxy ligand results in the formation of the final product, methoxyphenylsilane ((CH_3_O)SiH_2_Ph), with the concomitant regeneration of the manganese hydride complex **Mn6** (Scheme 57).

**Scheme 58:**
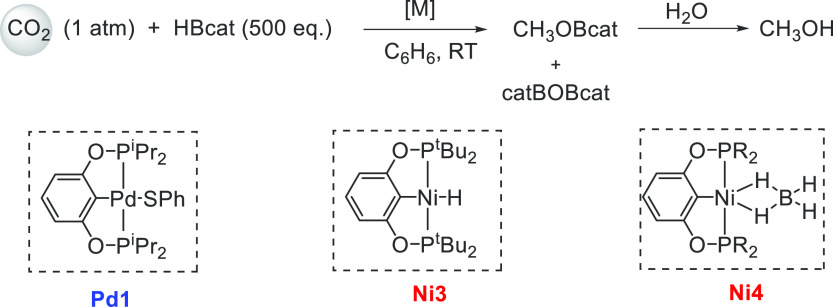
CO_2_ Hydroboration to Methanol Derivative Catalyzed by Pd and Ni Pincer Complexes

In addition to silanes, boranes were also used for CO_2_ capture via catalytic hydroboration followed by hydrolysis to methanol. Guan used Pd^382^ and Ni^383−385^ complexes of the POCOP pincer ligand, which catalyzed hydroboration of CO_2_ under atmospheric pressure to form a hydroborated product which was subsequently hydrolyzed to form methanol (Scheme 58). The Pd-based catalyst **Pd1** was found to be air-stable and hydroborated CO_2_ with catecholborane (HBcat) to form CH_3_OBcat and catBOBcat at room temperature with TOFs up to 1780 h^–1^.^382^ The nickel-based pincer complex **Ni3** catalyzed the same transformation at room temperature and an atmospheric pressure of CO_2_, although with a lower TOF of 495 h^–1^.^383^ The catalytic activity of analogous pincer complexes containing different PR_2_ (R = cyclopentyl, ^*i*^Pr, and ^*t*^Bu) groups at the **Ni3** complex were also studied, revealing that a more bulky substituent at the phosphine moiety favors the catalytic hydroboration reaction.^384^ Moreover, the same group also reported a nickel borohydride complex, **Ni4**, that catalyzed the hydroboration of CO_2_ at 1 atm pressure and 60 °C using 9-BBN (Scheme 58).^385^

### 4.2 Methanol Production from CO

**Scheme 59:**
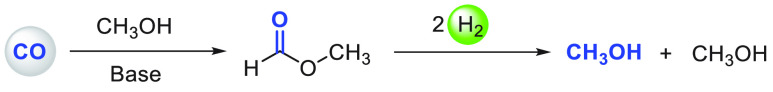
Hydrogenation of CO to CH_3_OH Using CH_3_OH as an Additive via Methyl Formate

Although synthesis of CH_3_OH directly from CO_2_ is more sustainable and attractive, several studies have also been carried out on the hydrogenation of CO to CH_3_OH. Hydrogenation of CO to CH_3_OH is more exothermic (Δ*H*_298 K_ = −90.7 kJ/mol) than that of CO_2_ to CH_3_OH (Δ*H*_298 K_ = −49.5 kJ/mol), which could allow the process to occur under relatively mild conditions. Seminal studies on the direct hydrogenation of CO to CH_3_OH using homogeneous catalysts were reported at harsh conditions.^386,387^ Recently, homogeneous catalysts have been employed for the indirect hydrogenation of CO to CH_3_OH in the presence of an additive such as alcohol or amine. This allows the process to occur under relatively mild conditions. For example, Mahajan^388^ and Jens^389,390^ independently demonstrated hydrogenation of CO to CH_3_OH via methyl formate (at 90–140 °C by Mahajan and at 60–120 °C by Jens). The reaction proceeds by insertion of CO into CH_3_OH to form methyl formate followed by hydrogenation to form CH_3_OH (Scheme 59).

**Scheme 60:**
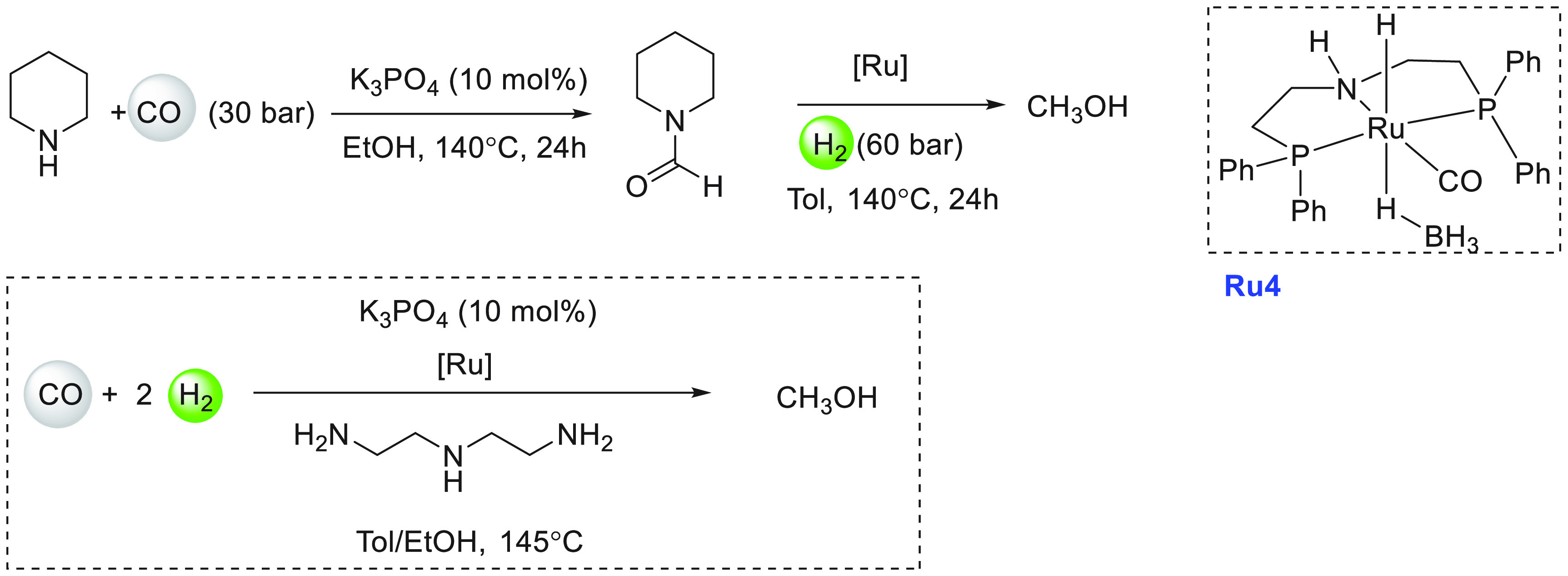
Methanol Synthesis from CO and H_2_ Using Amines and a Ru Pincer Complex

Along this line, amines were also used to capture CO to facilitate the hydrogenation process. Prakash recently reported the hydrogenation of CO to CH_3_OH by a two-step process: (1) trapping of CO with an amine such as piperidine to form formamide using K_3_PO_4_ catalyst and (2) hydrogenation of the formamide intermediate to CH_3_OH and piperidine catalyzed by the ruthenium pincer complex **Ru4** (Scheme 60).^391^ The yield of methanol increased when a polyamine diethylenetriamine was used instead of piperidine. Furthermore, direct hydrogenation of CO to CH_3_OH using diethylenetriamine and a catalytic combination of K_3_PO_4_ and the ruthenium catalyst **Ru4** was also demonstrated, exhibiting a TON up to 570 (Scheme 60). This process has several advantages, such as relatively low reaction temperature, absence of corrosive alkali metal alkoxide bases, inexpensive K_3_PO_4_, and inexpensive high-boiling polyamines.

**Scheme 61:**
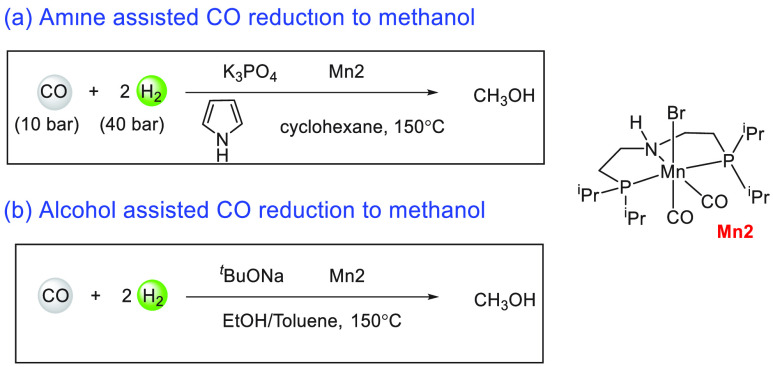
Amine- or Alcohol-Assisted Hydrogenation of CO to Methanol Catalyzed by a Mn Pincer Complex

An analogous transformation was also reported by the Beller group using a base-metal catalyst, **Mn2**, in combination with K_3_PO_4_ and an amine promoter (Scheme 61a).^392^ The type of amine was found to affect the yield of methanol. For example, TON > 500 was used in the case of indole, whereas scatole and pyrrole resulted in relatively high TONs of >3000 and 2500, respectively. Recently the Leitner group reported the alcohol assisted CO reduction to methanol using the Mn pincer complex (**Mn2**) resulting in a TON of 4023 and a TOF of 857 h^–1^ in EtOH/toluene as solvent under *p*(CO/H_2_) = 5/50 bar at 150 °C (Scheme 61b).^393^

### 4.3 Methanol Production from Formic Acid

**Scheme 62:**
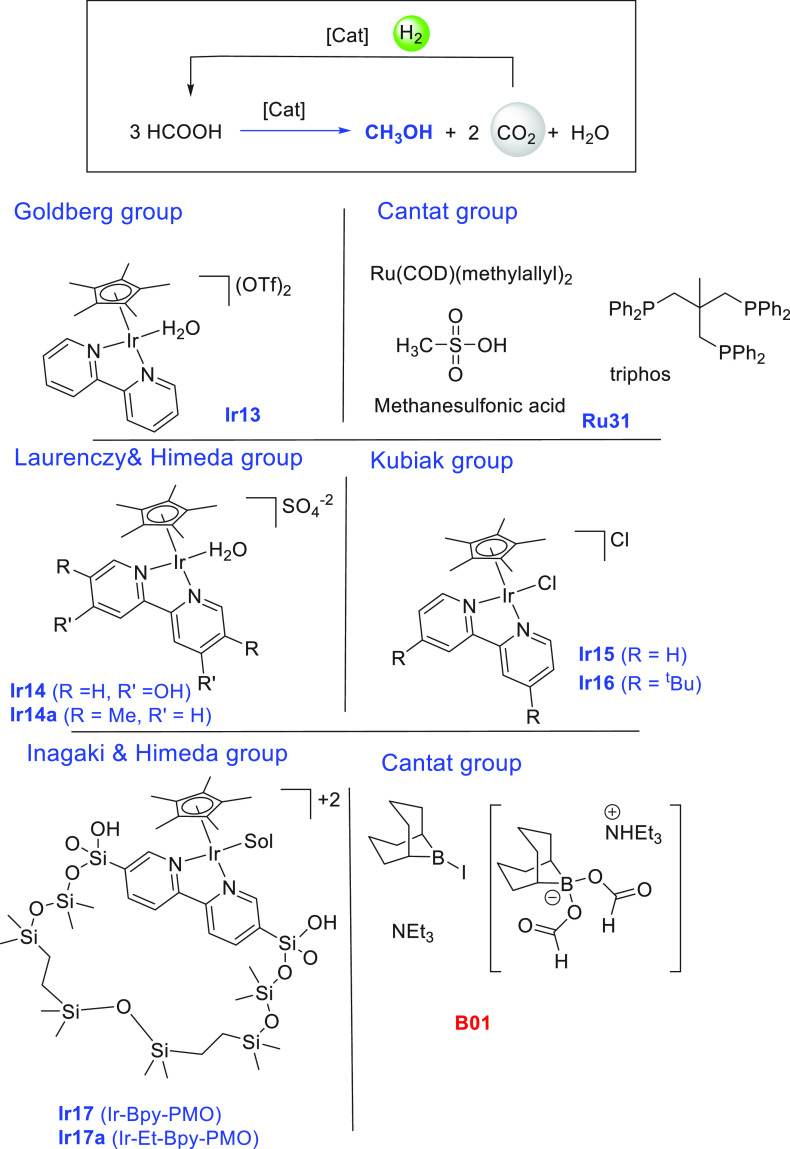
Formic Acid Decomposition to Methanol PMO = periodic mesoporous organosilica. Sol = solvent, e.g., acetonitrile.

The disproportionation of formic acid (FA) to MeOH and CO_2_ is also a promising method for the indirect conversion of CO_2_ to MeOH, especially as the area of hydrogenation of CO_2_ to formic acid/formate has been significantly developed in the past two decades (Scheme 62). The disproportionation reaction was first reported by Goldberg and co-workers in 2013 using the iridium catalyst [Cp*Ir(bpy)(H_2_O)](OTf)_2_ (**Ir13**).^394^ A TON up to 156 was observed; however, a low methanol selectivity (12%) was obtained. This was followed by a report from the Cantat group that utilized the ruthenium-based catalyst **Ru31** to produce methanol in 50.2% yield from the disproportionation reaction of FA.^395^ Laurenczy and Himeda demonstrated the same transformation with yields of up to 75% in D_2_O catalyzed by an iridium complex (**Ir14**), resulting in a TON of 240 (TON up to 1260 at the end of five cycles).^396^ Moreover, the same groups also reported a selective disproportionation of formic acid to methanol with a selectivity of 96% using the same catalyst (**Ir14**) under isochoric and acidic conditions.^397^ In 2016, the Kubiak group demonstrated the electronic effects on the catalytic disproportionation of HCOOH to CH_3_OH using cationic iridium bipyridine complexes (**Ir15**, **Ir16**) and noticed that the unsubstituted bipyridine complex and the 4-^*t*^Bu substituted complex exhibited the highest selectivity toward methanol.^398^ Soon after, Laurenczy and Himeda reported their study on various iridium catalysts with substituted 2,2′-bipyridine derivatives for disproportionation of formic acid. Their report revealed that the iridium catalyst bearing 5,5′-dimethyl-2,2′- bipyridine (**Ir14a**) showed high TON and selectivity toward methanol.^399^ Detailed DFT studies were also performed by Yang on this system.^400^ Furthermore, Himeda and Inagaki reported the catalytic disproportionation of HCOOH catalyzed by heterogeneous catalyst **Ir17** and homogeneous Ir-bipyridine catalysts. The analogous heterogeneous catalyst **Ir17a** was found to be more selective (up to 8.3%) for methanol production in comparison to the homogeneous Ir-bipyridine catalysts (1.4–4.3%).^401^ Neary and Parkin reported that the molybdenum complex Cp^R^Mo(PMe_3_)_3–*x*_(CO)_*x*_H afforded 21% selectivity of MeOH and methyl formate formation in benzene at 100 °C.^402^ In 2016, the Cantat group also reported a metal-free system for the formic acid disproportionation reaction using stoichiometric quantities of dialkylborane reagents (**B01**), where boroformate and borohydride intermediates were formed via the decarboxylation of formate and followed by undergoing disproportionation of formates to formaldehyde and methanol.^403^

### 4.4 Methanol Production from Methane

**Scheme 63:**
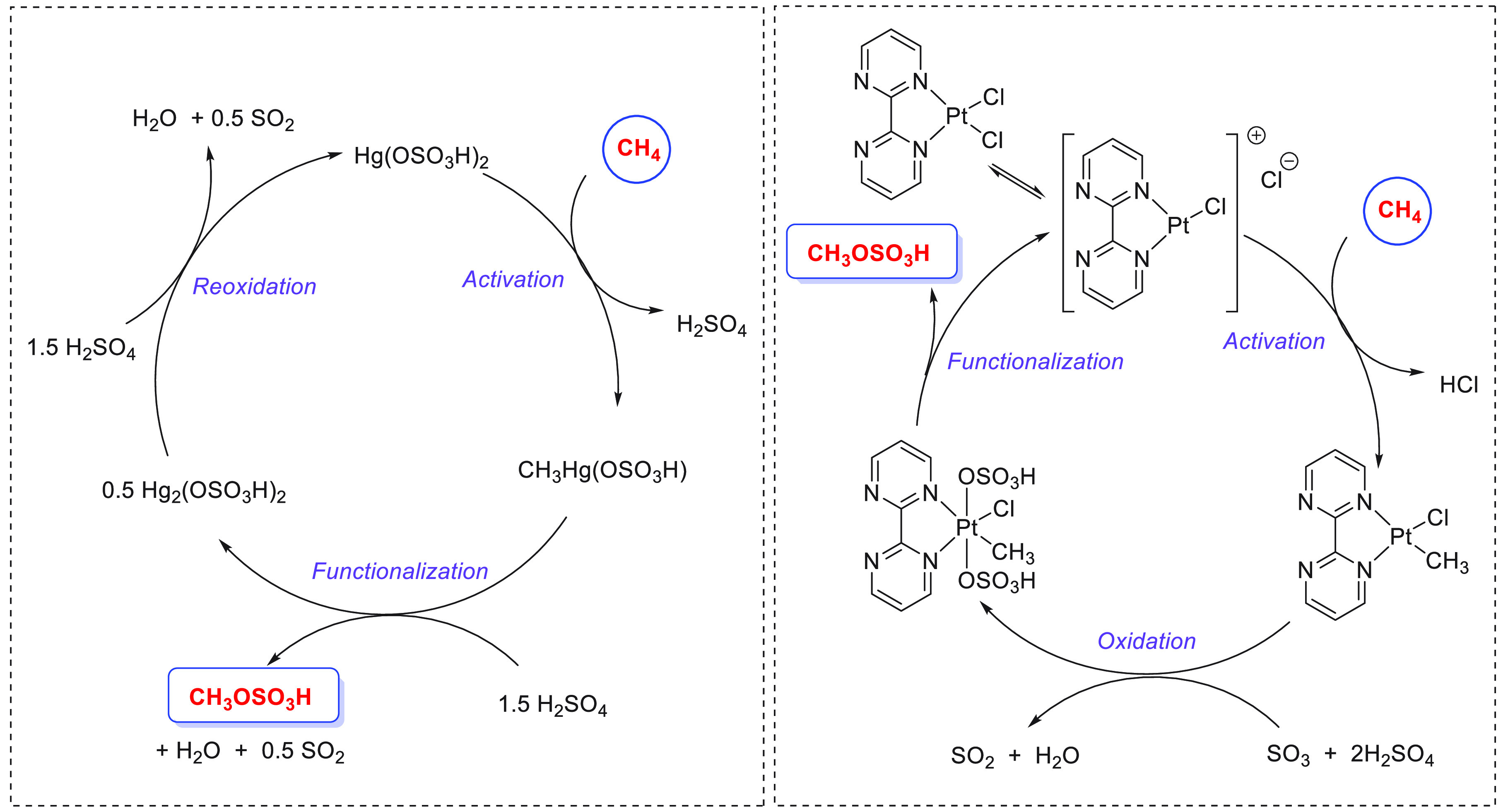
Proposed Mechanisms for the Formation of Methanol from Methane Using Mercuric Bisulfate and Platinum Bipyrimidine Catalysts

Methane, which constitutes 70–90% of natural gas,^26^ is a greenhouse gas having a 25 times larger impact than CO_2_ on global warming.^404^ As long-distance transport of natural gas (in gaseous form) is challenging, methane is flared to CO_2_ in oil fields, converting a potential resource to waste. Thus, sustainable methods for the conversion of CH_4_ to liquid fuels can play an important role in meeting the energy demands of the future. Current approaches for the transformation of CH_4_ to liquid fuels involve the transformation of CH_4_ to gasoline or syngas using the steam reforming process.^405^ However, both processes require harsh reaction conditions. Another approach that has been of high interest is partial oxidation of methane to methanol (CH_4_ + 1/2O_2_ → CH_3_OH), benefiting the methanol economy. Although the reaction is exothermic in nature (Δ*H*°_298 K_ = −126.2 kJ/mol), the process is highly challenging due to the associated kinetic barriers: (a) activation of the C–H bond in CH_4_ requires harsh reaction conditions due to the high dissociation energy of the C–H bond (440 kJ/mol) and (b) methanol C–H bond dissociation is 47 kJ/mol lower than that of CH_4_, making methanol susceptible for further oxidation under the reaction conditions. Thus, a suitable catalyst is required that can activate the methane C–H bond toward oxidation to methanol without overoxidation. Several heterogeneous catalysts have been reported for the partial oxidation of CH_4_ to CH_3_OH, although most of them work under high temperatures.^406^ A few homogeneous catalysts have also been reported for the oxidation of CH_4_ to CH_3_OH. A common challenge in the direct oxidation of CH_4_ to CH_3_OH is low yield and selectivity due to the kinetic limitations of this reaction. To address this issue, homogeneous catalysts have been employed to convert methane to methyl esters that can be hydrolyzed to produce methanol. A breakthrough in this direction was reported by Periana, who reported the conversion of methane to methanol via methyl bisulfate using a mercuric bisulfate catalyst.^407^ The catalysis starts with the C–H bond activation of methane by mercuric bisulfate (Hg(OSO_3_H)_2_) catalyst to produce an observable species, CH_3_HgOSO_3_H, eliminating H_2_SO_4_. This is followed by the decomposition of CH_3_HgOSO_3_H to CH_3_OSO_3_H and Hg_2_^2+^. Hg_2_^2+^ subsequently is oxidized by sulfuric acid to regenerate the active species Hg(OSO_3_H)_2_ and produce byproducts SO_2_ and H_2_O (Scheme 63). The same group later reported a catalyst involving a platinum bipyrimidine complex in H_2_SO_4_ that afforded a higher yield of methyl bisulfate (72%) compared to mercuric bisulfate catalyst that produced methyl bisulfate in 45% yield. The mechanism is similar to that of mercuric bisulfate catalyst as outlined in Scheme 63. After these seminal discoveries, several other groups have reported the oxidation of CH_4_ to methanol derivatives using homogeneous catalysts as reviewed recently.^408−410^ However, despite a good effort in the development of both heterogeneous and homogeneous catalysts for the direct oxidation of methane to methanol, there has been no commercial process in this direction. This is mainly because of the higher reactivity of methanol than methane and the thermodynamic formation of CO_2_ that leads to low yield (concentration) and poor selectivity of methanol, creating a need for the use of a stoichiometric additive.

## 5 Alkane Upgrading to Liquid Fuels

Lower alkanes can be derived from several sources, e.g., from biomass^411^ or by the reduction of CO_2_.^412^ Thus, alkane upgradation presents new opportunities to produce conventional petroleum-based fuel such as diesel and jet fuel (C9–C16) from renewable feedstocks (lower alkanes). However, the inertness of C–H bonds in unactivated alkanes makes this transformation challenging. Recent discoveries in catalysis have led to two new approaches for alkane upgradation: alkane metathesis and alkane–alkene coupling, as discussed below.

### 5.1 Alkane Metathesis

Due to the requirement of high temperature to activate the inert C–H bonds of alkanes, alkane metathesis was first reported using heterogeneous catalysis. Burnett and Hughes in 1973 reported the disproportionation of alkanes over a catalytic combination of platinum on alumina mixed with tungsten oxide on silica.^413^ This was followed by a series of papers from the Basset group describing the metathesis reaction of linear or branched alkanes producing the next higher and lower alkanes catalyzed by the silica-supported transition metal hydrides (based on tantalum, chromium, or tungsten) at moderate temperatures of 25–200 °C. An informative review of the earlier work on alkane metathesis was reported by Basset.^414^

**Scheme 64:**
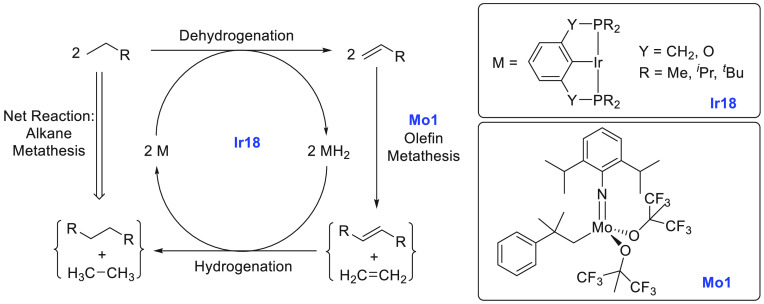
Cross Alkane Metathesis Involving (De)hydrogenation and Olefin Metathesis^416^ Reproduced from ref (416). Copyright 2017 American Chemical Society.

The first homogeneous catalytic system for alkane metathesis was discovered by Goldman, Brookhart, and co-workers in 2006.^415^ Using a tandem combination of two independent catalysts, an iridium pincer catalyst for alkane (de)hydrogenation and Schrock’s **Mo1** catalyst for olefin metathesis, efficient and selective metathesis of linear alkanes was achieved at a moderate temperature. The alkane metathesis proceeds by first alkane dehydrogenation to olefins followed by olefin metathesis to form one higher-chain and another lower-chain olefin molecules. The generated olefins are then hydrogenated by H_2_ formed in the dehydrogenation step (Scheme 64). For example, heating an *n*-hexane solution containing **Ir18** and **Mo1** at 125 °C in a sealed system under argon resulted in the formation of a range of C2–C15 *n*-alkanes in 24 h. Interestingly, no branched or cyclic alkanes were detected, unlike that reported by Basset, Copéret, and co-workers.^414^ A combination of the iridium pincer catalyst (MeO-^*i*^PrPCP)IrH_4_ with the metathesis catalyst Re_2_O_7_/Al_2_O_3_ showed a significant improvement in turnover numbers.

**Scheme 65:**
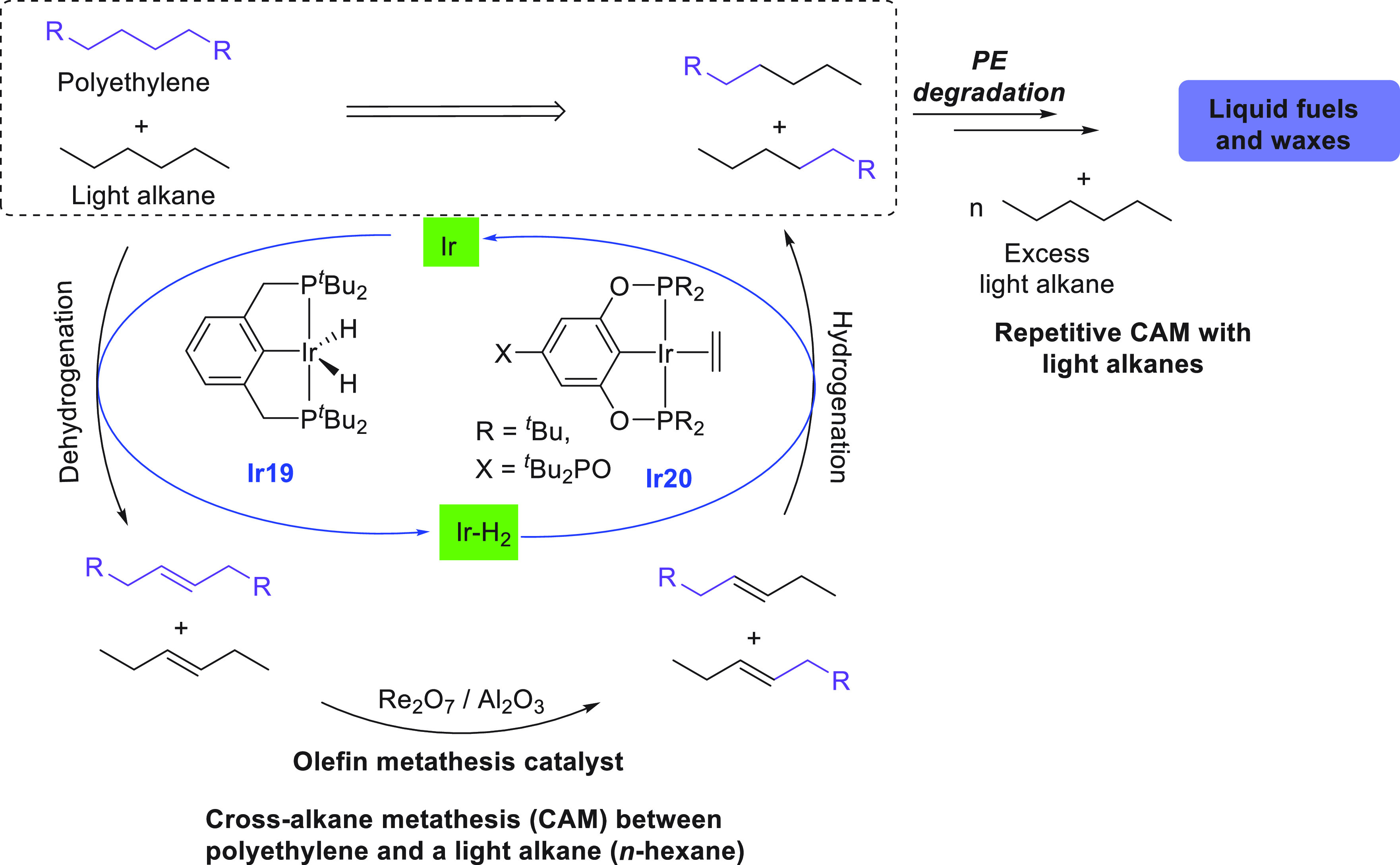
Proposed Pathway for the Degradation of PE Using Cross Alkane Metathesis (CAM) via Alkane Dehydrogenation Catalyzed by Iridium Pincer Complexes

Guan and Huang utilized the concept of tandem, catalytic cross alkane metathesis (CAM) for the degradation of polyethylenes (PEs) into liquid fuels and waxes.^417^ A mixture of polyethylene and a light alkane was heated under argon in the presence of a combination of the iridium pincer complex **Ir15** used for alkane dehydrogenation and Re_2_O_7_/Al_2_O_3_ catalyst for the purpose of olefin metathesis. As depicted in Scheme 65, the overall process occurs in three steps. In the first step, the iridium pincer complex dehydrogenates both the PE and the light alkane to form olefins and an Ir–H_2_ complex. This is followed by the scrambling of olefins catalyzed by the olefin metathesis catalyst, resulting in the breaking down of PE. Finally, the IrH_2_ complex generated in the first step transfers H_2_ to olefins, resulting in the formation of alkanes. Thus, the metathesis of polyethylene with light alkanes reduces the chain length of polyethylene and eventually leads to the formation of shorter hydrocarbons suitable for transportation oils. To demonstrate the proof of concept, 120 mg of high density polyethylene [powder; MW = 3350; polydispersity index (PDI) = 1.6] and 3 mL of *n*-hexane were heated at 150 °C in a sealed vessel in the presence of the iridium catalyst **Ir19** (20.1 μmol), 40.2 μmol of *tert*-butylethylene as a hydrogen acceptor, and Re_2_O_7_/γ-Al_2_O_3_ (57 μmol of Re_2_O_7_). Analysis of the reaction mixture upon completion showed the formation of a significant amount of C_22–40_*n*-alkanes (oil, 56% PE degradation to oils). Remarkably, no aromatic hydrocarbon or olefins were detected by GC, although the formation of a high molecular weight wax hydrocarbon (53 mg) insoluble in *n*-alkanes was obtained. Although the overall catalytic process was successfully demonstrated, the yield of oil products was modest (56%).

According to the predicted mechanism, a significant reduction of PE chain length requires the metathesis of the internal olefins generated from PE with an internal olefin of a light alkane. Hence, the complex (*^t^*Bu_2_PO-*^t^*Bu_2_POCOP^*t*^Bu_2_)Ir(C_2_H_4_) (**Ir20**) was chosen as a catalyst as it was previously reported to produce significant amounts of internal alkenes.^418^ Remarkably, this catalyst (supported on γ-Al_2_O_3_) resulted in the formation of oil products in 98% yield. Moreover, the recyclability of the catalyst and PE degradation at reduced catalytic loading was also demonstrated. For practical purposes, degradation of common plastic wastes such as postconsumer polyethylene bottles, bags, and films was also demonstrated without any pretreatment.

### 5.2 Alkane–Alkene Coupling

**Scheme 66:**
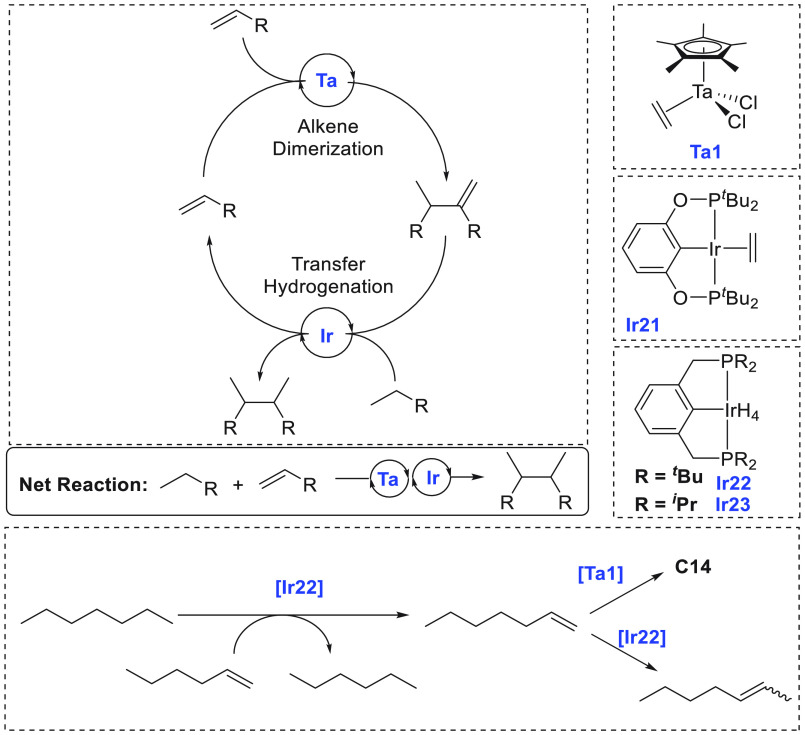
Proposed Mechanism for Alkane–Alkene Coupling Using Ir and Ta Catalysts

Although alkane metathesis is a major breakthrough in the direction of alkane upgrading, it produces a statistical distribution of products with limited selectivity of the desired weight fraction. A complementary approach for alkane upgrading was reported by Bercaw and Labinger, based on the coupling of an alkane and alkene having the same number of carbons.^419,420^ The hypothesis for the tandem catalytic system involves an alkene dimerization catalyst performing alkene-C*n* upgrading via dimerization and a transfer hydrogenation catalyst transforming upgraded alkene-C2*n* to alkane-C2*n*. Concomitantly, an equivalent amount of alkene-C*n* will be formed during transfer hydrogenation that will continue the catalytic cycle (Scheme 66).

For initial experiments, Cp*TaCl_2_(alkene) **Ta1**, originally reported by Schrock and demonstrated to be a highly active catalyst for selective dimerization of 1-alkenes,^421^ and [POCOP]Ir catalyst **Ir21**, were employed for the coupling of 1-hexene and *n*-heptane (Scheme 66). Monitoring the reaction by GC showed almost complete consumption of 1-hexene. *n*-Hexane and C12 hexene dimers were observed as the major products. However, no C13 or C14 products were detected by GC, suggesting that catalyst **Ir21** might not be suitable for the formation of 1-heptene. Interestingly, using a catalytic combination of **Ta1** (16 mM) and the iridium pincer complex **Ir22** (10 mM), known for the selective formation of terminal alkenes, C13 and C14 alkenes were formed in a combined yield of 22%. The formation of C12/C13/C14 alkanes was not observed. Several catalytic conditions were screened to improve the yield of the product. A better yield of C13/C14 alkene products was achieved by maintaining a low and steady concentration of 1-hexene. Mechanistic investigations also suggest that both catalysts perform independently without inhibiting each other’s catalytic activity.

In a similar direction, Goldman and co-workers reported (in a patent) a new strategy for alkane upgradation where an alkane is dehydrogenated by an iridium pincer catalyst and then undergoes oligomerization producing higher-chain olefins which subsequently are transfer hydrogenated to form higher-chain alkanes.^422^

## 6 Ethylene Glycol Production from CO and H_2_

**Scheme 67:**
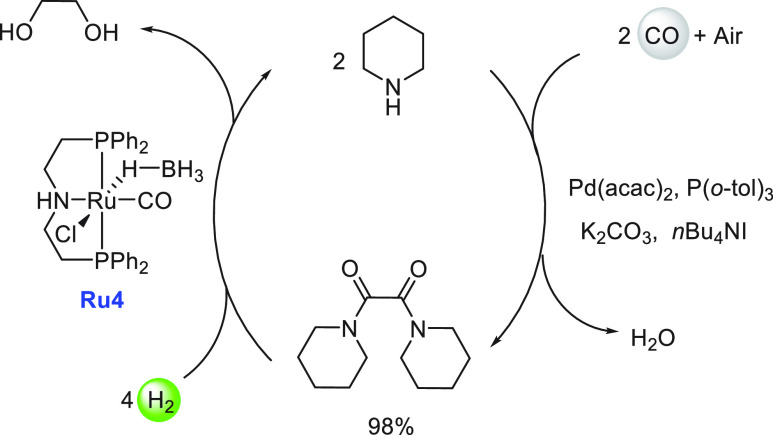
Formation of Ethylene Glycol from CO and H_2_ Using Piperidine

The use of ethylene glycol as a fuel in fuel cells has attracted significant interest recently.^423,424^ In addition to potential application in the energy sector, ethylene glycol is a valuable chemical feedstock with applications in the manufacture of polyesters, e.g., PET resins (polyethylene terephthalate), and as a solvent and antifreeze agent.^423^ Current industrial production of ethylene glycol involves oxidation of ethylene to ethylene oxide under harsh reaction conditions followed by hydrogenation. With the constant demand for ethylene glycol, an improved and sustainable synthesis of ethylene glycol from inexpensive and renewable feedstock is desirable. In 2016, Beller reported a new two-step method based on oxidative coupling of an amine with CO followed by hydrogenation to produce ethylene glycol (Scheme 67).^425^ The first step was based on an earlier report on Pd-catalyzed oxycarbonylation of amines to oxamides by Pri-Bar and Alper.^426^ After screening of several conditions, carbonylation of piperidine to 1,1-oxalyl dipiperidine was performed under 25 bar CO and 25 bar air, catalyzed by Pd(acac)_2_ (0.01 mol %), P(*o*-tol)_3_ (0.4 mol %), K_2_CO_3_ (10 mol %), and ^*n*^Bu_4_NI (2 mol %), at 120 °C leading to a TOF up to 750 h^–1^ and 98% yield. The second step was catalyzed by a combination of Ru-MACHO-BH (**Ru4**, 0.1 mol %) and KO^*t*^Bu (2 mol %), resulting in the complete conversion of 1,1′-oxalyl dipiperidine forming ethylene glycol in 94% yield in 24 h (in toluene). Remarkably, an iron-MACHO complex (2 mol %) and KOH (5 mol %) also catalyzed the hydrogenation of 1,1′-oxalyl dipiperidine to ethylene glycol in 77% yield. The reaction tolerated the addition of water with no change in the catalytic activity. Moreover, separation of the desired ethylene glycol from the reaction mixture (toluene and piperidine) was straightforward due to the formation of a biphasic mixture, and ethylene glycol was isolated in ∼95% yield.

**Scheme 68:**
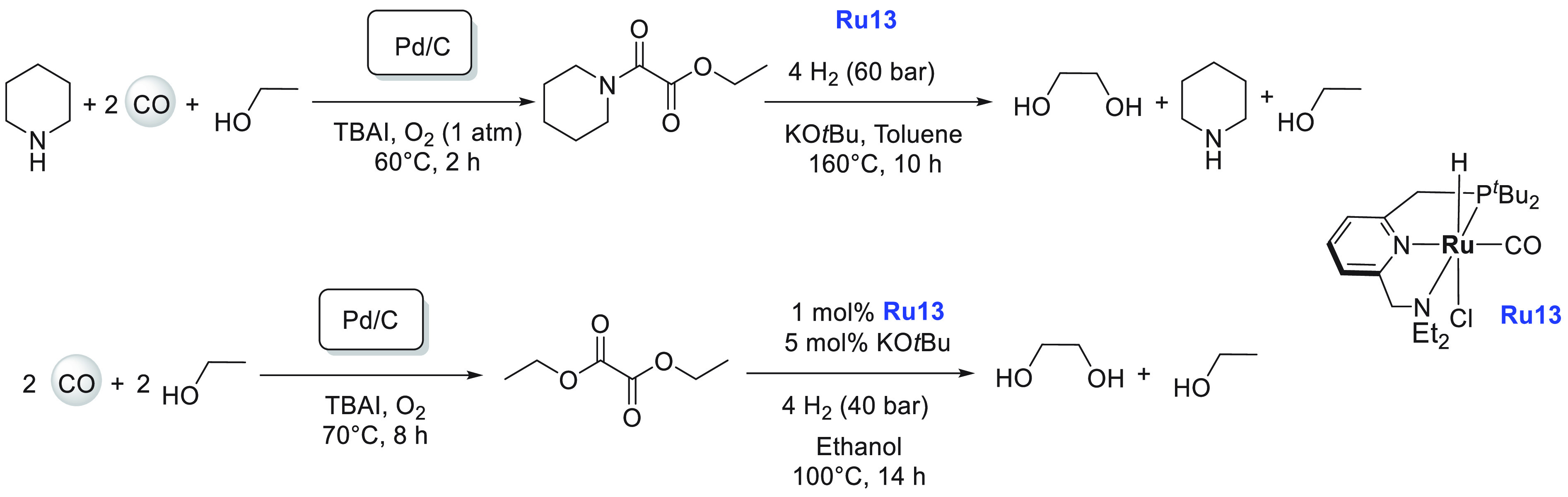
Synthesis of Ethylene Glycol from CO and H_2_ Assisted by Piperidine and Ethanol

Soon after, Bhanage reported a modified two-step synthesis of ethylene glycol using oxidative carbonylation of piperidine and ethanol followed by subsequent hydrogenation of the product oxamates using the Milstein catalyst **Ru13** (Scheme 68).^427^ The first step, oxidative carbonylation of piperidine and ethanol, was performed by an earlier established method reported by the same group.^428^ Conditions for the second step, hydrogenation of oxamides, were optimized by using several ruthenium catalysts and varying the temperature, solvent, and base. The best results were obtained using Milstein’s RuPNN catalyst **Ru13** (1 mol %) that produced ethylene glycol in 92% yield under the conditions of 60 bar H_2_, 160 °C, and 10 h in toluene solvent.

Followed by this work, Bhanage recently reported a similar two-step synthesis of ethylene glycol with oxidative carbonylation of ethanol using CO/O_2_ (25:6 atm) forming diethyl oxalate followed by subsequent hydrogenation (Scheme 68). The first step was catalyzed by Pd/C (10 mol %) in the presence of Bu_4_NI (TBAI, 0.2 mmol) at 70 °C.^429^ Several ruthenium catalysts were screened for the second step: hydrogenation of diethyl oxalate to ethylene glycol and ethanol. The best results were obtained using Milstein’s RuPNN catalyst **Ru13** (1 mol %) that afforded a 92% yield of ethylene glycol using 40 bar H_2_ at 100 °C in 14 h. Recyclability of the catalyst was also demonstrated for up to four cycles without any noticeable difference in performance.

## 7 Summary and Outlook

As discussed above, many well-defined transition metal complexes have been developed in recent years for their catalytic applications in the production and storage of several energy carriers. Catalytic (de)hydrogenative transformations to enable efficient storage of H_2_ have been an important focus of this review. Production of H_2_ from methanol via aqueous methanol reforming reaction has been studied well in the past using both heterogeneous and homogeneous catalysts (section 2.1). Well-defined transition metal complexes such as ruthenium pincer complexes represent the state-of-the-art catalysts for the low-temperature reforming of methanol (MeOH + H_2_O → CO_2_ + 3H_2_, hydrogen storage capacity 12.0 wt %). As the direct hydrogenation of CO_2_ to methanol has been possible industrially,^330^ methanol reforming/CO_2_ hydrogenation is a promising approach for a reversible hydrogen storage system.

Whereas a plethora of studies has been dedicated to exploring the dehydrogenation of aqueous methanol, dehydrogenation of formaldehyde has received only scant attention (section 2.2). Dehydrogenation of aqueous formaldehyde (H_2_CO + H_2_O → CO_2_ + 2H_2_, hydrogen storage capacity 8.4 wt %) is highly exothermic (Δ*H*_r_ = −35.8 kJ mol^–1^) compared to the dehydrogenation of aqueous methanol, which is highly endothermic (Δ*H*_r_ = 53.3 kJ mol^–1^), and thus provides thermodynamic advantages. However, the reaction also poses a challenge due to catalyst poisoning from the coordination of CO gas resulting from the decarbonylation of HCHO. New catalyst design could facilitate the application of aqueous HCHO for the purpose of reversible hydrogen storage.

Another potential hydrogen storage material that has been extensively reported in the literature and briefly discussed in this review is amine-borane (section 2.3). Dehydrogenation of amine-boranes is thermodynamically downhill, and a large number of homogeneous catalysts have been studied for their dehydrogenation. However, its practical application as a hydrogen storage material is still far away. This is because of the difficulty in the regeneration of “charged amine-borane fuel” from the “spent amine-borane fuel”. To overcome this, cyclic amine-boranes as a hybrid of inorganic (BN fragment) and organic (C–C fragment) components were synthesized and dehydrogenated. However, the hydrogenation of “spent fuel” in those cases using molecular hydrogen has still not been demonstrated. Regeneration of a “charged fuel” such as H_3_B·NH_3_ from direct hydrogenation of a “spent-fuel” such as borazine is still an unsolved problem in front of the catalysis community.

In the direction of developing reversible hydrogen storage materials based on organic liquids, also called liquid organic hydrogen carriers (LOHCs), several organic compounds have been investigated (section 2.4). Dehydrogenation of HCOOH (HCOOH → CO_2_ + H_2_) has been well investigated; however, its reverse reaction, i.e., hydrogenation of CO_2_ to HCOOH, often requires a basic medium to drive the reaction forward by forming a formate salt. This poses a hindrance in using HCOOH as a hydrogen storage material for commercial purposes as the regeneration of HCOOH from a formate salt requires a stoichiometric amount of acid. A few examples have been reported for base-free hydrogenation of CO_2_ to HCOOH that uses either an acidic buffer or a polar protic solvent such as DMSO or an ionic liquid containing an anion that stabilizes HCOOH through H-bonding interactions. The area of hydrogenation of CO_2_ to HCOOH under additive-free conditions is still in its preliminary stage. New catalyst designs could facilitate this transformation that could potentially make CO_2_/HCOOH a commercial hydrogen storage material. Several LOHCs involving alcohols and amines have also been developed in the past few years. Ru pincer complexes are the state-of-the-art catalysts for such (de)hydrogenative transformations. The advantages of using alcohols/amines as LOHCs are that they are inexpensive and, in some cases, renewable, whose dehydrogenation to form esters/amides, as well as the reverse hydrogenation reactions, is facile and occurs under relatively mild conditions. A common limitation for most of such catalytic (de)hydrogenative systems is the use of a solvent in catalysis, thus significantly reducing the hydrogen storage capacity. This presents opportunities to explore organic compounds that can be dehydrogenated under neat conditions and can be utilized as hydrogen storage materials. Renewable diols such as ethylene glycol/polyester have also been demonstrated for a potential LOHC (section 2.4.3). Along this line, utilization of a glycerol/polyester couple (up to 6.52 wt %) as an LOHC can be a significant breakthrough benefiting both the hydrogen economy and the circular economy simultaneously. It is noteworthy that glycerol is currently being produced in a surplus quantity as a byproduct of the biodiesel industry and therefore utilization of glycerol to make a renewable and recyclable polyester can significantly benefit the circular economy.^430^ In Table 5 we have outlined a comparative summary of the developments and limitations of various hydrogen carriers mediated by homogeneous catalysts discussed in this review.

**Table 5:** Comparative Summary of Hydrogen Carriers (Catalyst Development and Limitations) Discussed in This Review

hydrogen carrier	dehydrogenation reaction	max capacity	highlights
CH_3_OH + H_2_O	CH_3_OH + H_2_O → 3H_2_ + CO_2_	12.0 wt %	Heterogeneous catalysts operate under high temperature (>200 °C) and pressure (25–50 bar).
			Homogeneous catalysts can enable the transformation under mild conditions (e.g., temperature 70–100 °C) with TON up to 353 409 (24 days) but suffer from limitations such as the use of base and solvents.
HCHO + H_2_O	HCHO + H_2_O → 2H_2_ + CO_2_	8.4 wt %	Only a handful number of catalysts have been reported.
			Catalysts reported to operate under basic, neutral, and acidic conditions.
			No report using a base-metal homogeneous catalyst.
RH_2_B·NH_2_R (amine-boranes)	RH_2_B·NH_2_R → “BN” + *n*H_2_	up to 12.96 wt %	Several catalysts reported for the facile dehydrogenation process.
			Limitations due to its solid state and difficulty in the regeneration of the charged fuel (amine-boranes) from the spent “BN” fuel.
N-heterocycles	N-heterocycle (saturated) → N-heterocycle (unsaturated) + *n*H_2_	up to 7.2 wt %	Most of the systems reported using heterogeneous catalysts.
			Only a few examples using homogeneous catalysts have been reported with limitations of using solvents or exhibiting relatively lower hydrogen storage capacity.
diols	ethylene glycol → oligoesters + *n*H_2_	up to 6.5 wt %	Only one catalyst reported for ethylene glycol/oligoesters hydrogen storage couple. Dehydrogenation can be performed under neat (solvent-free) condition under static vacuum.
	1,4-butanediol → lactone + 2H_2_	up to 4.4 wt %	
alcohols + amines	alcohol + amine → amide + *n*H_2_	up to 6.7 wt %	Pincer catalysts represent the state-of-the-art catalysts for these transformations.
			Processes suffer from the limitation of using solvents with the scope of improvement for TON and catalyst recyclability.
HCOOH	HCOONa + H_2_O → HOCOONa + H_2_	2.32 wt %	Low hydrogen storage capacity.
	HCOOH → CO_2_ + H_2_	4.4 wt %	Most of the catalysts require an additive such as a base.

The approach of producing conventional fuels (hydrocarbons) from biomass such as ethanol, vegetable oil, and lignin has also been discussed here (section 3). Several homogeneous catalysts for upgradation of ethanol to butanol have been reported, but selective upgradation of ethanol to higher alcohols such as octanol and decanol has not been accomplished yet. The area of lignin depolymerization to produce useful fuel has been mostly studied by using heterogeneous catalysts, presenting opportunities for new homogeneous catalyst design that could be effective for depolymerization of lignin via C–O cleavage.

The role of homogeneous catalysts for the methanol economy has been discussed in detail in terms of the production of CH_3_OH by the hydrogenation of CO_2_ or CO (section 4). Direct hydrogenation of CO_2_ to methanol has been mostly investigated by heterogeneous catalysts. In fact, 100% renewable methanol is produced from the hydrogenation of CO_2_ to methanol using the “emission to liquid technology” by Carbon Recycling International. Homogenous catalysts have been utilized for the indirect conversion of CO_2_ to CH_3_OH, where CO_2_ is trapped with reagents such as alcohol, amines, silanes or boranes, and then subsequently hydrogenated or hydrolyzed to form methanol and regenerate the trapping agent. The development of new homogeneous catalysts for the direct hydrogenation of CO_2_ to CH_3_OH under mild conditions is still a challenge in the area of molecular chemistry. An interesting development in this direction has been made recently by Himeda and Onishi, who have utilized the approach of a gas–solid phase reaction for the hydrogenation of CO_2_ to methanol under mild conditions (e.g., 60 °C, 40 bar, 3:1 H_2_/CO_2_, TON up to 113).^431^ The reaction is catalyzed by a multinuclear iridium complex capable of performing intramolecular multiple hydride transfer to CO_2_. The success of the catalytic methodology was partly attributed to the solid–gas phase reaction approach that suppresses the liberation of formic acid which was found to inhibit this transformation in the aqueous phase due to its decomposition to H_2_ and CO_2_. Another challenging topic where homogeneous catalysts can make a significant impact is the direct partial oxidation of methane to CH_3_OH as discussed above (section 4.4).

Alkane upgradation through the approaches of cross alkane metathesis and alkane–alkene coupling presents attractive opportunities to produce fuels from biomass such as CH_4_ (section 5). However, methane upgradation has not been demonstrated yet due to a high barrier associated with C–H activation of CH_4_, and further coupling reactions. Moreover, the development of alkene/alkane metathesis catalysts involving complexes of earth-abundant metals will also be a breakthrough and enhance the sustainability of various processes based on the metathesis reaction.

Based on the above discussions, we present here significant challenges in homogeneous catalysis to be achieved under relatively mild conditions, for the development of sustainable energy carriers:

(1) direct hydrogenation of CO_2_ to CH_3_OH without using an additive

(2) direct hydrogenation of “spent” B–N fuel (e.g., aminoborane, borazine, polyaminoborane) to “charged” B–N fuel (amine-boranes)

(3) development of glycerol/polyester as a hydrogen storage couple to benefit both hydrogen economy and circular economy simultaneously

(4) direct hydrogenation of CO_2_ to HCOOH without using an additive or special solvent

(5) ethanol upgrading to higher alcohols (e.g., octanol–cetyl alcohol)

(6) C–O cleavage of aryl ethers (model lignin compounds) using H_2_ without using a stoichiometric additive

(7) methane upgradation and direct partial oxidation of methane to methanol (CH_4_ + 1/2O_2_ → CH_3_OH) with high yield and selectivity

(8) alkane dehydrogenation/metathesis reaction using earth-abundant metal catalysts

Although a few advances have been made on some of these topics, we believe that further development in homogeneous catalysis can lead to a paradigm shift in the advent of new sustainable technologies for the production and storage of energy carriers.
